# Supplementary material for: How does agonistic behaviour differ in albino and pigmented fish?
Source: PeerJ. 2016 Apr 18;4:e1937. doi: 10.7717/peerj.1937 (PMC4841223; doi:10.7717/peerj.1937)
Supplement: Supplemental Information 1 [file peerj-04-1937-s001.pdf]

treatment	trial	weight1	weight2	no	time_grid	prob	behaviour
AxA	1_1	8	10	1	5	0	latdisplay
AxA	1_1	8	10	1	10	0	latdisplay
AxA	1_1	8	10	1	15	0	latdisplay
AxA	1_1	8	10	1	20	0	latdisplay
AxA	1_1	8	10	1	25	0	latdisplay
AxA	1_1	8	10	1	30	0	latdisplay
AxA	1_1	8	10	1	35	1	latdisplay
AxA	1_1	8	10	1	40	1	latdisplay
AxA	1_1	8	10	1	45	0	latdisplay
AxA	1_1	8	10	1	50	1	latdisplay
AxA	1_1	8	10	1	55	0	latdisplay
AxA	1_1	8	10	1	60	0	latdisplay
AxA	1_1	8	10	1	65	0	latdisplay
AxA	1_1	8	10	1	70	0	latdisplay
AxA	1_1	8	10	1	75	0	latdisplay
AxA	1_1	8	10	1	80	1	latdisplay
AxA	1_1	8	10	1	85	0	latdisplay
AxA	1_1	8	10	1	90	1	latdisplay
AxA	1_1	8	10	1	95	0	latdisplay
AxA	1_1	8	10	1	100	0	latdisplay
AxA	1_1	8	10	1	105	0	latdisplay
AxA	1_1	8	10	1	110	1	latdisplay
AxA	1_1	8	10	1	115	0	latdisplay
AxA	1_1	8	10	1	120	0	latdisplay
AxA	1_1	8	10	1	125	0	latdisplay
AxA	1_1	8	10	1	130	0	latdisplay
AxA	1_1	8	10	1	135	0	latdisplay
AxA	1_1	8	10	1	140	0	latdisplay
AxA	1_1	8	10	1	145	0	latdisplay
AxA	1_1	8	10	1	150	0	latdisplay
AxA	1_1	8	10	1	155	1	latdisplay
AxA	1_1	8	10	1	160	1	latdisplay
AxA	1_1	8	10	1	165	0	latdisplay
AxA	1_1	8	10	1	170	1	latdisplay
AxA	1_1	8	10	1	175	0	latdisplay
AxA	1_1	8	10	1	180	0	latdisplay
AxA	1_1	8	10	1	185	0	latdisplay
AxA	1_1	8	10	1	190	0	latdisplay
AxA	1_1	8	10	1	195	0	latdisplay
AxA	1_1	8	10	1	200	0	latdisplay
AxA	1_1	8	10	1	205	1	latdisplay
AxA	1_1	8	10	1	210	0	latdisplay
AxA	1_1	8	10	1	215	1	latdisplay
AxA	1_1	8	10	1	220	0	latdisplay
AxA	1_1	8	10	1	225	0	latdisplay
AxA	1_1	8	10	1	230	0	latdisplay
AxA	1_1	8	10	1	235	0	latdisplay

AxA	1_1	8	10	1	240	1	latdisplay
AxA	1_1	8	10	1	245	0	latdisplay
AxA	1_1	8	10	1	250	0	latdisplay
AxA	1_1	8	10	1	255	0	latdisplay
AxA	1_1	8	10	1	260	0	latdisplay
AxA	1_1	8	10	1	265	0	latdisplay
AxA	1_1	8	10	1	270	0	latdisplay
AxA	1_1	8	10	1	275	0	latdisplay
AxA	1_1	8	10	1	280	1	latdisplay
AxA	1_1	8	10	1	285	0	latdisplay
AxA	1_1	8	10	1	290	1	latdisplay
AxA	1_1	8	10	1	295	1	latdisplay
AxA	1_1	8	10	1	300	0	latdisplay
AxA	2_1	9	7	2	5	0	latdisplay
AxA	2_1	9	7	2	10	0	latdisplay
AxA	2_1	9	7	2	15	0	latdisplay
AxA	2_1	9	7	2	20	0	latdisplay
AxA	2_1	9	7	2	25	0	latdisplay
AxA	2_1	9	7	2	30	0	latdisplay
AxA	2_1	9	7	2	35	1	latdisplay
AxA	2_1	9	7	2	40	0	latdisplay
AxA	2_1	9	7	2	45	1	latdisplay
AxA	2_1	9	7	2	50	0	latdisplay
AxA	2_1	9	7	2	55	1	latdisplay
AxA	2_1	9	7	2	60	0	latdisplay
AxA	2_1	9	7	2	65	0	latdisplay
AxA	2_1	9	7	2	70	0	latdisplay
AxA	2_1	9	7	2	75	0	latdisplay
AxA	2_1	9	7	2	80	0	latdisplay
AxA	2_1	9	7	2	85	0	latdisplay
AxA	2_1	9	7	2	90	1	latdisplay
AxA	2_1	9	7	2	95	0	latdisplay
AxA	2_1	9	7	2	100	0	latdisplay
AxA	2_1	9	7	2	105	0	latdisplay
AxA	2_1	9	7	2	110	0	latdisplay
AxA	2_1	9	7	2	115	0	latdisplay
AxA	2_1	9	7	2	120	0	latdisplay
AxA	2_1	9	7	2	125	0	latdisplay
AxA	2_1	9	7	2	130	0	latdisplay
AxA	2_1	9	7	2	135	0	latdisplay
AxA	2_1	9	7	2	140	0	latdisplay
AxA	2_1	9	7	2	145	0	latdisplay
AxA	2_1	9	7	2	150	0	latdisplay
AxA	2_1	9	7	2	155	0	latdisplay
AxA	2_1	9	7	2	160	0	latdisplay
AxA	2_1	9	7	2	165	0	latdisplay
AxA	2_1	9	7	2	170	0	latdisplay
AxA	2_1	9	7	2	175	0	latdisplay

AxA	2_1	9	7	2	180	0	latdisplay
AxA	2_1	9	7	2	185	0	latdisplay
AxA	2_1	9	7	2	190	0	latdisplay
AxA	2_1	9	7	2	195	0	latdisplay
AxA	2_1	9	7	2	200	0	latdisplay
AxA	2_1	9	7	2	205	0	latdisplay
AxA	2_1	9	7	2	210	0	latdisplay
AxA	2_1	9	7	2	215	1	latdisplay
AxA	2_1	9	7	2	220	0	latdisplay
AxA	2_1	9	7	2	225	0	latdisplay
AxA	2_1	9	7	2	230	0	latdisplay
AxA	2_1	9	7	2	235	0	latdisplay
AxA	2_1	9	7	2	240	0	latdisplay
AxA	2_1	9	7	2	245	0	latdisplay
AxA	2_1	9	7	2	250	0	latdisplay
AxA	2_1	9	7	2	255	0	latdisplay
AxA	2_1	9	7	2	260	0	latdisplay
AxA	2_1	9	7	2	265	0	latdisplay
AxA	2_1	9	7	2	270	0	latdisplay
AxA	2_1	9	7	2	275	0	latdisplay
AxA	2_1	9	7	2	280	0	latdisplay
AxA	2_1	9	7	2	285	0	latdisplay
AxA	2_1	9	7	2	290	0	latdisplay
AxA	2_1	9	7	2	295	0	latdisplay
AxA	2_1	9	7	2	300	0	latdisplay
AxA	3_1	8	13	3	5	0	latdisplay
AxA	3_1	8	13	3	10	0	latdisplay
AxA	3_1	8	13	3	15	0	latdisplay
AxA	3_1	8	13	3	20	0	latdisplay
AxA	3_1	8	13	3	25	0	latdisplay
AxA	3_1	8	13	3	30	0	latdisplay
AxA	3_1	8	13	3	35	0	latdisplay
AxA	3_1	8	13	3	40	0	latdisplay
AxA	3_1	8	13	3	45	0	latdisplay
AxA	3_1	8	13	3	50	0	latdisplay
AxA	3_1	8	13	3	55	0	latdisplay
AxA	3_1	8	13	3	60	0	latdisplay
AxA	3_1	8	13	3	65	0	latdisplay
AxA	3_1	8	13	3	70	0	latdisplay
AxA	3_1	8	13	3	75	1	latdisplay
AxA	3_1	8	13	3	80	0	latdisplay
AxA	3_1	8	13	3	85	0	latdisplay
AxA	3_1	8	13	3	90	0	latdisplay
AxA	3_1	8	13	3	95	1	latdisplay
AxA	3_1	8	13	3	100	0	latdisplay
AxA	3_1	8	13	3	105	0	latdisplay
AxA	3_1	8	13	3	110	0	latdisplay
AxA	3_1	8	13	3	115	0	latdisplay

AxA	3_1	8	13	3	120	1	latdisplay
AxA	3_1	8	13	3	125	0	latdisplay
AxA	3_1	8	13	3	130	0	latdisplay
AxA	3_1	8	13	3	135	0	latdisplay
AxA	3_1	8	13	3	140	1	latdisplay
AxA	3_1	8	13	3	145	0	latdisplay
AxA	3_1	8	13	3	150	0	latdisplay
AxA	3_1	8	13	3	155	0	latdisplay
AxA	3_1	8	13	3	160	1	latdisplay
AxA	3_1	8	13	3	165	1	latdisplay
AxA	3_1	8	13	3	170	0	latdisplay
AxA	3_1	8	13	3	175	0	latdisplay
AxA	3_1	8	13	3	180	1	latdisplay
AxA	3_1	8	13	3	185	1	latdisplay
AxA	3_1	8	13	3	190	0	latdisplay
AxA	3_1	8	13	3	195	1	latdisplay
AxA	3_1	8	13	3	200	1	latdisplay
AxA	3_1	8	13	3	205	1	latdisplay
AxA	3_1	8	13	3	210	0	latdisplay
AxA	3_1	8	13	3	215	1	latdisplay
AxA	3_1	8	13	3	220	1	latdisplay
AxA	3_1	8	13	3	225	0	latdisplay
AxA	3_1	8	13	3	230	0	latdisplay
AxA	3_1	8	13	3	235	0	latdisplay
AxA	3_1	8	13	3	240	0	latdisplay
AxA	3_1	8	13	3	245	0	latdisplay
AxA	3_1	8	13	3	250	0	latdisplay
AxA	3_1	8	13	3	255	0	latdisplay
AxA	3_1	8	13	3	260	1	latdisplay
AxA	3_1	8	13	3	265	0	latdisplay
AxA	3_1	8	13	3	270	1	latdisplay
AxA	3_1	8	13	3	275	0	latdisplay
AxA	3_1	8	13	3	280	1	latdisplay
AxA	3_1	8	13	3	285	0	latdisplay
AxA	3_1	8	13	3	290	0	latdisplay
AxA	3_1	8	13	3	295	0	latdisplay
AxA	3_1	8	13	3	300	0	latdisplay
AxA	4_1	11	10	4	5	0	latdisplay
AxA	4_1	11	10	4	10	0	latdisplay
AxA	4_1	11	10	4	15	0	latdisplay
AxA	4_1	11	10	4	20	0	latdisplay
AxA	4_1	11	10	4	25	0	latdisplay
AxA	4_1	11	10	4	30	0	latdisplay
AxA	4_1	11	10	4	35	0	latdisplay
AxA	4_1	11	10	4	40	0	latdisplay
AxA	4_1	11	10	4	45	0	latdisplay
AxA	4_1	11	10	4	50	0	latdisplay
AxA	4_1	11	10	4	55	0	latdisplay

AxA	4_1	11	10	4	60	0	latdisplay
AxA	4_1	11	10	4	65	0	latdisplay
AxA	4_1	11	10	4	70	1	latdisplay
AxA	4_1	11	10	4	75	0	latdisplay
AxA	4_1	11	10	4	80	0	latdisplay
AxA	4_1	11	10	4	85	0	latdisplay
AxA	4_1	11	10	4	90	0	latdisplay
AxA	4_1	11	10	4	95	0	latdisplay
AxA	4_1	11	10	4	100	0	latdisplay
AxA	4_1	11	10	4	105	0	latdisplay
AxA	4_1	11	10	4	110	0	latdisplay
AxA	4_1	11	10	4	115	1	latdisplay
AxA	4_1	11	10	4	120	0	latdisplay
AxA	4_1	11	10	4	125	1	latdisplay
AxA	4_1	11	10	4	130	0	latdisplay
AxA	4_1	11	10	4	135	1	latdisplay
AxA	4_1	11	10	4	140	0	latdisplay
AxA	4_1	11	10	4	145	1	latdisplay
AxA	4_1	11	10	4	150	1	latdisplay
AxA	4_1	11	10	4	155	1	latdisplay
AxA	4_1	11	10	4	160	0	latdisplay
AxA	4_1	11	10	4	165	1	latdisplay
AxA	4_1	11	10	4	170	1	latdisplay
AxA	4_1	11	10	4	175	1	latdisplay
AxA	4_1	11	10	4	180	0	latdisplay
AxA	4_1	11	10	4	185	1	latdisplay
AxA	4_1	11	10	4	190	0	latdisplay
AxA	4_1	11	10	4	195	1	latdisplay
AxA	4_1	11	10	4	200	1	latdisplay
AxA	4_1	11	10	4	205	0	latdisplay
AxA	4_1	11	10	4	210	0	latdisplay
AxA	4_1	11	10	4	215	0	latdisplay
AxA	4_1	11	10	4	220	0	latdisplay
AxA	4_1	11	10	4	225	0	latdisplay
AxA	4_1	11	10	4	230	1	latdisplay
AxA	4_1	11	10	4	235	0	latdisplay
AxA	4_1	11	10	4	240	1	latdisplay
AxA	4_1	11	10	4	245	1	latdisplay
AxA	4_1	11	10	4	250	0	latdisplay
AxA	4_1	11	10	4	255	0	latdisplay
AxA	4_1	11	10	4	260	0	latdisplay
AxA	4_1	11	10	4	265	1	latdisplay
AxA	4_1	11	10	4	270	1	latdisplay
AxA	4_1	11	10	4	275	0	latdisplay
AxA	4_1	11	10	4	280	0	latdisplay
AxA	4_1	11	10	4	285	1	latdisplay
AxA	4_1	11	10	4	290	0	latdisplay
AxA	4_1	11	10	4	295	0	latdisplay

AxA	4_1	11	10	4	300	0	latdisplay
AxA	5_1	11	12	5	5	0	latdisplay
AxA	5_1	11	12	5	10	0	latdisplay
AxA	5_1	11	12	5	15	0	latdisplay
AxA	5_1	11	12	5	20	0	latdisplay
AxA	5_1	11	12	5	25	0	latdisplay
AxA	5_1	11	12	5	30	0	latdisplay
AxA	5_1	11	12	5	35	0	latdisplay
AxA	5_1	11	12	5	40	0	latdisplay
AxA	5_1	11	12	5	45	0	latdisplay
AxA	5_1	11	12	5	50	1	latdisplay
AxA	5_1	11	12	5	55	0	latdisplay
AxA	5_1	11	12	5	60	1	latdisplay
AxA	5_1	11	12	5	65	0	latdisplay
AxA	5_1	11	12	5	70	0	latdisplay
AxA	5_1	11	12	5	75	0	latdisplay
AxA	5_1	11	12	5	80	0	latdisplay
AxA	5_1	11	12	5	85	0	latdisplay
AxA	5_1	11	12	5	90	0	latdisplay
AxA	5_1	11	12	5	95	0	latdisplay
AxA	5_1	11	12	5	100	0	latdisplay
AxA	5_1	11	12	5	105	0	latdisplay
AxA	5_1	11	12	5	110	0	latdisplay
AxA	5_1	11	12	5	115	0	latdisplay
AxA	5_1	11	12	5	120	0	latdisplay
AxA	5_1	11	12	5	125	0	latdisplay
AxA	5_1	11	12	5	130	0	latdisplay
AxA	5_1	11	12	5	135	0	latdisplay
AxA	5_1	11	12	5	140	1	latdisplay
AxA	5_1	11	12	5	145	0	latdisplay
AxA	5_1	11	12	5	150	1	latdisplay
AxA	5_1	11	12	5	155	0	latdisplay
AxA	5_1	11	12	5	160	0	latdisplay
AxA	5_1	11	12	5	165	1	latdisplay
AxA	5_1	11	12	5	170	1	latdisplay
AxA	5_1	11	12	5	175	1	latdisplay
AxA	5_1	11	12	5	180	0	latdisplay
AxA	5_1	11	12	5	185	0	latdisplay
AxA	5_1	11	12	5	190	0	latdisplay
AxA	5_1	11	12	5	195	0	latdisplay
AxA	5_1	11	12	5	200	0	latdisplay
AxA	5_1	11	12	5	205	0	latdisplay
AxA	5_1	11	12	5	210	0	latdisplay
AxA	5_1	11	12	5	215	0	latdisplay
AxA	5_1	11	12	5	220	0	latdisplay
AxA	5_1	11	12	5	225	0	latdisplay
AxA	5_1	11	12	5	230	0	latdisplay
AxA	5_1	11	12	5	235	0	latdisplay

AxA	5_1	11	12	5	240	0	latdisplay
AxA	5_1	11	12	5	245	0	latdisplay
AxA	5_1	11	12	5	250	0	latdisplay
AxA	5_1	11	12	5	255	0	latdisplay
AxA	5_1	11	12	5	260	0	latdisplay
AxA	5_1	11	12	5	265	0	latdisplay
AxA	5_1	11	12	5	270	0	latdisplay
AxA	5_1	11	12	5	275	1	latdisplay
AxA	5_1	11	12	5	280	0	latdisplay
AxA	5_1	11	12	5	285	0	latdisplay
AxA	5_1	11	12	5	290	1	latdisplay
AxA	5_1	11	12	5	295	0	latdisplay
AxA	5_1	11	12	5	300	1	latdisplay
AxA	6_1	13	11	6	5	0	latdisplay
AxA	6_1	13	11	6	10	0	latdisplay
AxA	6_1	13	11	6	15	0	latdisplay
AxA	6_1	13	11	6	20	0	latdisplay
AxA	6_1	13	11	6	25	0	latdisplay
AxA	6_1	13	11	6	30	0	latdisplay
AxA	6_1	13	11	6	35	0	latdisplay
AxA	6_1	13	11	6	40	0	latdisplay
AxA	6_1	13	11	6	45	0	latdisplay
AxA	6_1	13	11	6	50	0	latdisplay
AxA	6_1	13	11	6	55	0	latdisplay
AxA	6_1	13	11	6	60	0	latdisplay
AxA	6_1	13	11	6	65	0	latdisplay
AxA	6_1	13	11	6	70	0	latdisplay
AxA	6_1	13	11	6	75	0	latdisplay
AxA	6_1	13	11	6	80	0	latdisplay
AxA	6_1	13	11	6	85	0	latdisplay
AxA	6_1	13	11	6	90	1	latdisplay
AxA	6_1	13	11	6	95	0	latdisplay
AxA	6_1	13	11	6	100	0	latdisplay
AxA	6_1	13	11	6	105	0	latdisplay
AxA	6_1	13	11	6	110	1	latdisplay
AxA	6_1	13	11	6	115	0	latdisplay
AxA	6_1	13	11	6	120	0	latdisplay
AxA	6_1	13	11	6	125	1	latdisplay
AxA	6_1	13	11	6	130	1	latdisplay
AxA	6_1	13	11	6	135	0	latdisplay
AxA	6_1	13	11	6	140	1	latdisplay
AxA	6_1	13	11	6	145	0	latdisplay
AxA	6_1	13	11	6	150	1	latdisplay
AxA	6_1	13	11	6	155	0	latdisplay
AxA	6_1	13	11	6	160	0	latdisplay
AxA	6_1	13	11	6	165	0	latdisplay
AxA	6_1	13	11	6	170	0	latdisplay
AxA	6_1	13	11	6	175	0	latdisplay

AxA	6_1	13	11	6	180	0	latdisplay
AxA	6_1	13	11	6	185	0	latdisplay
AxA	6_1	13	11	6	190	0	latdisplay
AxA	6_1	13	11	6	195	0	latdisplay
AxA	6_1	13	11	6	200	0	latdisplay
AxA	6_1	13	11	6	205	0	latdisplay
AxA	6_1	13	11	6	210	0	latdisplay
AxA	6_1	13	11	6	215	0	latdisplay
AxA	6_1	13	11	6	220	0	latdisplay
AxA	6_1	13	11	6	225	0	latdisplay
AxA	6_1	13	11	6	230	0	latdisplay
AxA	6_1	13	11	6	235	0	latdisplay
AxA	6_1	13	11	6	240	0	latdisplay
AxA	6_1	13	11	6	245	0	latdisplay
AxA	6_1	13	11	6	250	0	latdisplay
AxA	6_1	13	11	6	255	0	latdisplay
AxA	6_1	13	11	6	260	0	latdisplay
AxA	6_1	13	11	6	265	0	latdisplay
AxA	6_1	13	11	6	270	0	latdisplay
AxA	6_1	13	11	6	275	0	latdisplay
AxA	6_1	13	11	6	280	0	latdisplay
AxA	6_1	13	11	6	285	0	latdisplay
AxA	6_1	13	11	6	290	0	latdisplay
AxA	6_1	13	11	6	295	0	latdisplay
AxA	6_1	13	11	6	300	1	latdisplay
AxA	7_1	10	12	7	5	0	latdisplay
AxA	7_1	10	12	7	10	0	latdisplay
AxA	7_1	10	12	7	15	0	latdisplay
AxA	7_1	10	12	7	20	0	latdisplay
AxA	7_1	10	12	7	25	0	latdisplay
AxA	7_1	10	12	7	30	0	latdisplay
AxA	7_1	10	12	7	35	0	latdisplay
AxA	7_1	10	12	7	40	0	latdisplay
AxA	7_1	10	12	7	45	0	latdisplay
AxA	7_1	10	12	7	50	0	latdisplay
AxA	7_1	10	12	7	55	0	latdisplay
AxA	7_1	10	12	7	60	0	latdisplay
AxA	7_1	10	12	7	65	0	latdisplay
AxA	7_1	10	12	7	70	0	latdisplay
AxA	7_1	10	12	7	75	0	latdisplay
AxA	7_1	10	12	7	80	0	latdisplay
AxA	7_1	10	12	7	85	0	latdisplay
AxA	7_1	10	12	7	90	0	latdisplay
AxA	7_1	10	12	7	95	0	latdisplay
AxA	7_1	10	12	7	100	0	latdisplay
AxA	7_1	10	12	7	105	0	latdisplay
AxA	7_1	10	12	7	110	0	latdisplay
AxA	7_1	10	12	7	115	0	latdisplay

AxA	7_1	10	12	7	120	0	latdisplay
AxA	7_1	10	12	7	125	0	latdisplay
AxA	7_1	10	12	7	130	0	latdisplay
AxA	7_1	10	12	7	135	0	latdisplay
AxA	7_1	10	12	7	140	1	latdisplay
AxA	7_1	10	12	7	145	0	latdisplay
AxA	7_1	10	12	7	150	0	latdisplay
AxA	7_1	10	12	7	155	0	latdisplay
AxA	7_1	10	12	7	160	0	latdisplay
AxA	7_1	10	12	7	165	0	latdisplay
AxA	7_1	10	12	7	170	1	latdisplay
AxA	7_1	10	12	7	175	0	latdisplay
AxA	7_1	10	12	7	180	0	latdisplay
AxA	7_1	10	12	7	185	0	latdisplay
AxA	7_1	10	12	7	190	0	latdisplay
AxA	7_1	10	12	7	195	0	latdisplay
AxA	7_1	10	12	7	200	0	latdisplay
AxA	7_1	10	12	7	205	0	latdisplay
AxA	7_1	10	12	7	210	0	latdisplay
AxA	7_1	10	12	7	215	1	latdisplay
AxA	7_1	10	12	7	220	0	latdisplay
AxA	7_1	10	12	7	225	0	latdisplay
AxA	7_1	10	12	7	230	0	latdisplay
AxA	7_1	10	12	7	235	0	latdisplay
AxA	7_1	10	12	7	240	0	latdisplay
AxA	7_1	10	12	7	245	0	latdisplay
AxA	7_1	10	12	7	250	0	latdisplay
AxA	7_1	10	12	7	255	1	latdisplay
AxA	7_1	10	12	7	260	1	latdisplay
AxA	7_1	10	12	7	265	0	latdisplay
AxA	7_1	10	12	7	270	0	latdisplay
AxA	7_1	10	12	7	275	0	latdisplay
AxA	7_1	10	12	7	280	0	latdisplay
AxA	7_1	10	12	7	285	0	latdisplay
AxA	7_1	10	12	7	290	0	latdisplay
AxA	7_1	10	12	7	295	0	latdisplay
AxA	7_1	10	12	7	300	0	latdisplay
AxA	8_1	12	11	8	5	0	latdisplay
AxA	8_1	12	11	8	10	0	latdisplay
AxA	8_1	12	11	8	15	0	latdisplay
AxA	8_1	12	11	8	20	0	latdisplay
AxA	8_1	12	11	8	25	0	latdisplay
AxA	8_1	12	11	8	30	0	latdisplay
AxA	8_1	12	11	8	35	0	latdisplay
AxA	8_1	12	11	8	40	0	latdisplay
AxA	8_1	12	11	8	45	0	latdisplay
AxA	8_1	12	11	8	50	0	latdisplay
AxA	8_1	12	11	8	55	0	latdisplay

AxA	8_1	12	11	8	60	0	latdisplay
AxA	8_1	12	11	8	65	0	latdisplay
AxA	8_1	12	11	8	70	0	latdisplay
AxA	8_1	12	11	8	75	0	latdisplay
AxA	8_1	12	11	8	80	0	latdisplay
AxA	8_1	12	11	8	85	0	latdisplay
AxA	8_1	12	11	8	90	0	latdisplay
AxA	8_1	12	11	8	95	0	latdisplay
AxA	8_1	12	11	8	100	0	latdisplay
AxA	8_1	12	11	8	105	0	latdisplay
AxA	8_1	12	11	8	110	0	latdisplay
AxA	8_1	12	11	8	115	0	latdisplay
AxA	8_1	12	11	8	120	0	latdisplay
AxA	8_1	12	11	8	125	0	latdisplay
AxA	8_1	12	11	8	130	0	latdisplay
AxA	8_1	12	11	8	135	0	latdisplay
AxA	8_1	12	11	8	140	0	latdisplay
AxA	8_1	12	11	8	145	0	latdisplay
AxA	8_1	12	11	8	150	0	latdisplay
AxA	8_1	12	11	8	155	0	latdisplay
AxA	8_1	12	11	8	160	0	latdisplay
AxA	8_1	12	11	8	165	1	latdisplay
AxA	8_1	12	11	8	170	0	latdisplay
AxA	8_1	12	11	8	175	0	latdisplay
AxA	8_1	12	11	8	180	0	latdisplay
AxA	8_1	12	11	8	185	0	latdisplay
AxA	8_1	12	11	8	190	0	latdisplay
AxA	8_1	12	11	8	195	1	latdisplay
AxA	8_1	12	11	8	200	0	latdisplay
AxA	8_1	12	11	8	205	0	latdisplay
AxA	8_1	12	11	8	210	1	latdisplay
AxA	8_1	12	11	8	215	0	latdisplay
AxA	8_1	12	11	8	220	0	latdisplay
AxA	8_1	12	11	8	225	0	latdisplay
AxA	8_1	12	11	8	230	0	latdisplay
AxA	8_1	12	11	8	235	0	latdisplay
AxA	8_1	12	11	8	240	0	latdisplay
AxA	8_1	12	11	8	245	0	latdisplay
AxA	8_1	12	11	8	250	0	latdisplay
AxA	8_1	12	11	8	255	0	latdisplay
AxA	8_1	12	11	8	260	0	latdisplay
AxA	8_1	12	11	8	265	0	latdisplay
AxA	8_1	12	11	8	270	0	latdisplay
AxA	8_1	12	11	8	275	0	latdisplay
AxA	8_1	12	11	8	280	0	latdisplay
AxA	8_1	12	11	8	285	0	latdisplay
AxA	8_1	12	11	8	290	0	latdisplay
AxA	8_1	12	11	8	295	0	latdisplay

AxA	8_1	12	11	8	300	0	latdisplay
AxA	9_1	9	11	9	5	0	latdisplay
AxA	9_1	9	11	9	10	0	latdisplay
AxA	9_1	9	11	9	15	0	latdisplay
AxA	9_1	9	11	9	20	0	latdisplay
AxA	9_1	9	11	9	25	0	latdisplay
AxA	9_1	9	11	9	30	0	latdisplay
AxA	9_1	9	11	9	35	0	latdisplay
AxA	9_1	9	11	9	40	0	latdisplay
AxA	9_1	9	11	9	45	0	latdisplay
AxA	9_1	9	11	9	50	0	latdisplay
AxA	9_1	9	11	9	55	0	latdisplay
AxA	9_1	9	11	9	60	0	latdisplay
AxA	9_1	9	11	9	65	0	latdisplay
AxA	9_1	9	11	9	70	0	latdisplay
AxA	9_1	9	11	9	75	0	latdisplay
AxA	9_1	9	11	9	80	0	latdisplay
AxA	9_1	9	11	9	85	0	latdisplay
AxA	9_1	9	11	9	90	0	latdisplay
AxA	9_1	9	11	9	95	0	latdisplay
AxA	9_1	9	11	9	100	0	latdisplay
AxA	9_1	9	11	9	105	0	latdisplay
AxA	9_1	9	11	9	110	0	latdisplay
AxA	9_1	9	11	9	115	0	latdisplay
AxA	9_1	9	11	9	120	0	latdisplay
AxA	9_1	9	11	9	125	0	latdisplay
AxA	9_1	9	11	9	130	1	latdisplay
AxA	9_1	9	11	9	135	0	latdisplay
AxA	9_1	9	11	9	140	0	latdisplay
AxA	9_1	9	11	9	145	0	latdisplay
AxA	9_1	9	11	9	150	0	latdisplay
AxA	9_1	9	11	9	155	0	latdisplay
AxA	9_1	9	11	9	160	0	latdisplay
AxA	9_1	9	11	9	165	0	latdisplay
AxA	9_1	9	11	9	170	0	latdisplay
AxA	9_1	9	11	9	175	0	latdisplay
AxA	9_1	9	11	9	180	0	latdisplay
AxA	9_1	9	11	9	185	0	latdisplay
AxA	9_1	9	11	9	190	0	latdisplay
AxA	9_1	9	11	9	195	0	latdisplay
AxA	9_1	9	11	9	200	0	latdisplay
AxA	9_1	9	11	9	205	0	latdisplay
AxA	9_1	9	11	9	210	1	latdisplay
AxA	9_1	9	11	9	215	0	latdisplay
AxA	9_1	9	11	9	220	0	latdisplay
AxA	9_1	9	11	9	225	0	latdisplay
AxA	9_1	9	11	9	230	0	latdisplay
AxA	9_1	9	11	9	235	0	latdisplay

AxA	9_1	9	11	9	240	0	latdisplay
AxA	9_1	9	11	9	245	1	latdisplay
AxA	9_1	9	11	9	250	0	latdisplay
AxA	9_1	9	11	9	255	0	latdisplay
AxA	9_1	9	11	9	260	0	latdisplay
AxA	9_1	9	11	9	265	0	latdisplay
AxA	9_1	9	11	9	270	0	latdisplay
AxA	9_1	9	11	9	275	0	latdisplay
AxA	9_1	9	11	9	280	0	latdisplay
AxA	9_1	9	11	9	285	0	latdisplay
AxA	9_1	9	11	9	290	0	latdisplay
AxA	9_1	9	11	9	295	0	latdisplay
AxA	9_1	9	11	9	300	0	latdisplay
AxA	10_1	13	12	10	5	0	latdisplay
AxA	10_1	13	12	10	10	0	latdisplay
AxA	10_1	13	12	10	15	0	latdisplay
AxA	10_1	13	12	10	20	0	latdisplay
AxA	10_1	13	12	10	25	0	latdisplay
AxA	10_1	13	12	10	30	0	latdisplay
AxA	10_1	13	12	10	35	1	latdisplay
AxA	10_1	13	12	10	40	1	latdisplay
AxA	10_1	13	12	10	45	1	latdisplay
AxA	10_1	13	12	10	50	1	latdisplay
AxA	10_1	13	12	10	55	1	latdisplay
AxA	10_1	13	12	10	60	0	latdisplay
AxA	10_1	13	12	10	65	1	latdisplay
AxA	10_1	13	12	10	70	1	latdisplay
AxA	10_1	13	12	10	75	0	latdisplay
AxA	10_1	13	12	10	80	1	latdisplay
AxA	10_1	13	12	10	85	0	latdisplay
AxA	10_1	13	12	10	90	0	latdisplay
AxA	10_1	13	12	10	95	0	latdisplay
AxA	10_1	13	12	10	100	0	latdisplay
AxA	10_1	13	12	10	105	1	latdisplay
AxA	10_1	13	12	10	110	1	latdisplay
AxA	10_1	13	12	10	115	0	latdisplay
AxA	10_1	13	12	10	120	0	latdisplay
AxA	10_1	13	12	10	125	1	latdisplay
AxA	10_1	13	12	10	130	0	latdisplay
AxA	10_1	13	12	10	135	0	latdisplay
AxA	10_1	13	12	10	140	0	latdisplay
AxA	10_1	13	12	10	145	1	latdisplay
AxA	10_1	13	12	10	150	0	latdisplay
AxA	10_1	13	12	10	155	0	latdisplay
AxA	10_1	13	12	10	160	0	latdisplay
AxA	10_1	13	12	10	165	0	latdisplay
AxA	10_1	13	12	10	170	1	latdisplay
AxA	10_1	13	12	10	175	0	latdisplay

AxA	10_1	13	12	10	180	0	latdisplay
AxA	10_1	13	12	10	185	0	latdisplay
AxA	10_1	13	12	10	190	1	latdisplay
AxA	10_1	13	12	10	195	1	latdisplay
AxA	10_1	13	12	10	200	1	latdisplay
AxA	10_1	13	12	10	205	1	latdisplay
AxA	10_1	13	12	10	210	1	latdisplay
AxA	10_1	13	12	10	215	0	latdisplay
AxA	10_1	13	12	10	220	0	latdisplay
AxA	10_1	13	12	10	225	0	latdisplay
AxA	10_1	13	12	10	230	0	latdisplay
AxA	10_1	13	12	10	235	0	latdisplay
AxA	10_1	13	12	10	240	0	latdisplay
AxA	10_1	13	12	10	245	0	latdisplay
AxA	10_1	13	12	10	250	1	latdisplay
AxA	10_1	13	12	10	255	1	latdisplay
AxA	10_1	13	12	10	260	1	latdisplay
AxA	10_1	13	12	10	265	0	latdisplay
AxA	10_1	13	12	10	270	0	latdisplay
AxA	10_1	13	12	10	275	1	latdisplay
AxA	10_1	13	12	10	280	0	latdisplay
AxA	10_1	13	12	10	285	0	latdisplay
AxA	10_1	13	12	10	290	0	latdisplay
AxA	10_1	13	12	10	295	0	latdisplay
AxA	10_1	13	12	10	300	1	latdisplay
AxA	11_1	9	10	11	5	0	latdisplay
AxA	11_1	9	10	11	10	0	latdisplay
AxA	11_1	9	10	11	15	0	latdisplay
AxA	11_1	9	10	11	20	0	latdisplay
AxA	11_1	9	10	11	25	0	latdisplay
AxA	11_1	9	10	11	30	0	latdisplay
AxA	11_1	9	10	11	35	0	latdisplay
AxA	11_1	9	10	11	40	0	latdisplay
AxA	11_1	9	10	11	45	0	latdisplay
AxA	11_1	9	10	11	50	0	latdisplay
AxA	11_1	9	10	11	55	1	latdisplay
AxA	11_1	9	10	11	60	1	latdisplay
AxA	11_1	9	10	11	65	1	latdisplay
AxA	11_1	9	10	11	70	0	latdisplay
AxA	11_1	9	10	11	75	0	latdisplay
AxA	11_1	9	10	11	80	0	latdisplay
AxA	11_1	9	10	11	85	0	latdisplay
AxA	11_1	9	10	11	90	0	latdisplay
AxA	11_1	9	10	11	95	0	latdisplay
AxA	11_1	9	10	11	100	1	latdisplay
AxA	11_1	9	10	11	105	1	latdisplay
AxA	11_1	9	10	11	110	0	latdisplay
AxA	11_1	9	10	11	115	0	latdisplay

AxA	11_1	9	10	11	120	0	latdisplay
AxA	11_1	9	10	11	125	1	latdisplay
AxA	11_1	9	10	11	130	0	latdisplay
AxA	11_1	9	10	11	135	0	latdisplay
AxA	11_1	9	10	11	140	0	latdisplay
AxA	11_1	9	10	11	145	0	latdisplay
AxA	11_1	9	10	11	150	0	latdisplay
AxA	11_1	9	10	11	155	0	latdisplay
AxA	11_1	9	10	11	160	0	latdisplay
AxA	11_1	9	10	11	165	0	latdisplay
AxA	11_1	9	10	11	170	0	latdisplay
AxA	11_1	9	10	11	175	0	latdisplay
AxA	11_1	9	10	11	180	1	latdisplay
AxA	11_1	9	10	11	185	0	latdisplay
AxA	11_1	9	10	11	190	0	latdisplay
AxA	11_1	9	10	11	195	0	latdisplay
AxA	11_1	9	10	11	200	1	latdisplay
AxA	11_1	9	10	11	205	1	latdisplay
AxA	11_1	9	10	11	210	1	latdisplay
AxA	11_1	9	10	11	215	0	latdisplay
AxA	11_1	9	10	11	220	0	latdisplay
AxA	11_1	9	10	11	225	0	latdisplay
AxA	11_1	9	10	11	230	1	latdisplay
AxA	11_1	9	10	11	235	0	latdisplay
AxA	11_1	9	10	11	240	0	latdisplay
AxA	11_1	9	10	11	245	0	latdisplay
AxA	11_1	9	10	11	250	0	latdisplay
AxA	11_1	9	10	11	255	0	latdisplay
AxA	11_1	9	10	11	260	0	latdisplay
AxA	11_1	9	10	11	265	0	latdisplay
AxA	11_1	9	10	11	270	0	latdisplay
AxA	11_1	9	10	11	275	0	latdisplay
AxA	11_1	9	10	11	280	0	latdisplay
AxA	11_1	9	10	11	285	0	latdisplay
AxA	11_1	9	10	11	290	0	latdisplay
AxA	11_1	9	10	11	295	0	latdisplay
AxA	11_1	9	10	11	300	0	latdisplay
AxA	12_1	13	13	12	5	0	latdisplay
AxA	12_1	13	13	12	10	0	latdisplay
AxA	12_1	13	13	12	15	0	latdisplay
AxA	12_1	13	13	12	20	0	latdisplay
AxA	12_1	13	13	12	25	0	latdisplay
AxA	12_1	13	13	12	30	0	latdisplay
AxA	12_1	13	13	12	35	0	latdisplay
AxA	12_1	13	13	12	40	1	latdisplay
AxA	12_1	13	13	12	45	0	latdisplay
AxA	12_1	13	13	12	50	0	latdisplay
AxA	12_1	13	13	12	55	0	latdisplay

AxA	12_1	13	13	12	60	0	latdisplay
AxA	12_1	13	13	12	65	0	latdisplay
AxA	12_1	13	13	12	70	0	latdisplay
AxA	12_1	13	13	12	75	1	latdisplay
AxA	12_1	13	13	12	80	0	latdisplay
AxA	12_1	13	13	12	85	0	latdisplay
AxA	12_1	13	13	12	90	0	latdisplay
AxA	12_1	13	13	12	95	0	latdisplay
AxA	12_1	13	13	12	100	1	latdisplay
AxA	12_1	13	13	12	105	0	latdisplay
AxA	12_1	13	13	12	110	0	latdisplay
AxA	12_1	13	13	12	115	1	latdisplay
AxA	12_1	13	13	12	120	0	latdisplay
AxA	12_1	13	13	12	125	0	latdisplay
AxA	12_1	13	13	12	130	0	latdisplay
AxA	12_1	13	13	12	135	0	latdisplay
AxA	12_1	13	13	12	140	0	latdisplay
AxA	12_1	13	13	12	145	0	latdisplay
AxA	12_1	13	13	12	150	0	latdisplay
AxA	12_1	13	13	12	155	0	latdisplay
AxA	12_1	13	13	12	160	0	latdisplay
AxA	12_1	13	13	12	165	0	latdisplay
AxA	12_1	13	13	12	170	0	latdisplay
AxA	12_1	13	13	12	175	0	latdisplay
AxA	12_1	13	13	12	180	0	latdisplay
AxA	12_1	13	13	12	185	0	latdisplay
AxA	12_1	13	13	12	190	1	latdisplay
AxA	12_1	13	13	12	195	0	latdisplay
AxA	12_1	13	13	12	200	0	latdisplay
AxA	12_1	13	13	12	205	0	latdisplay
AxA	12_1	13	13	12	210	0	latdisplay
AxA	12_1	13	13	12	215	0	latdisplay
AxA	12_1	13	13	12	220	0	latdisplay
AxA	12_1	13	13	12	225	0	latdisplay
AxA	12_1	13	13	12	230	0	latdisplay
AxA	12_1	13	13	12	235	0	latdisplay
AxA	12_1	13	13	12	240	0	latdisplay
AxA	12_1	13	13	12	245	0	latdisplay
AxA	12_1	13	13	12	250	0	latdisplay
AxA	12_1	13	13	12	255	0	latdisplay
AxA	12_1	13	13	12	260	0	latdisplay
AxA	12_1	13	13	12	265	0	latdisplay
AxA	12_1	13	13	12	270	0	latdisplay
AxA	12_1	13	13	12	275	0	latdisplay
AxA	12_1	13	13	12	280	0	latdisplay
AxA	12_1	13	13	12	285	0	latdisplay
AxA	12_1	13	13	12	290	0	latdisplay
AxA	12_1	13	13	12	295	0	latdisplay

AxA	12_1	13	13	12	300	0	latdisplay
AxA	13_1	15	13	13	5	0	latdisplay
AxA	13_1	15	13	13	10	0	latdisplay
AxA	13_1	15	13	13	15	0	latdisplay
AxA	13_1	15	13	13	20	0	latdisplay
AxA	13_1	15	13	13	25	0	latdisplay
AxA	13_1	15	13	13	30	0	latdisplay
AxA	13_1	15	13	13	35	0	latdisplay
AxA	13_1	15	13	13	40	0	latdisplay
AxA	13_1	15	13	13	45	0	latdisplay
AxA	13_1	15	13	13	50	0	latdisplay
AxA	13_1	15	13	13	55	0	latdisplay
AxA	13_1	15	13	13	60	0	latdisplay
AxA	13_1	15	13	13	65	0	latdisplay
AxA	13_1	15	13	13	70	0	latdisplay
AxA	13_1	15	13	13	75	0	latdisplay
AxA	13_1	15	13	13	80	0	latdisplay
AxA	13_1	15	13	13	85	0	latdisplay
AxA	13_1	15	13	13	90	0	latdisplay
AxA	13_1	15	13	13	95	0	latdisplay
AxA	13_1	15	13	13	100	0	latdisplay
AxA	13_1	15	13	13	105	0	latdisplay
AxA	13_1	15	13	13	110	0	latdisplay
AxA	13_1	15	13	13	115	0	latdisplay
AxA	13_1	15	13	13	120	0	latdisplay
AxA	13_1	15	13	13	125	0	latdisplay
AxA	13_1	15	13	13	130	0	latdisplay
AxA	13_1	15	13	13	135	0	latdisplay
AxA	13_1	15	13	13	140	0	latdisplay
AxA	13_1	15	13	13	145	0	latdisplay
AxA	13_1	15	13	13	150	0	latdisplay
AxA	13_1	15	13	13	155	0	latdisplay
AxA	13_1	15	13	13	160	0	latdisplay
AxA	13_1	15	13	13	165	0	latdisplay
AxA	13_1	15	13	13	170	0	latdisplay
AxA	13_1	15	13	13	175	0	latdisplay
AxA	13_1	15	13	13	180	0	latdisplay
AxA	13_1	15	13	13	185	0	latdisplay
AxA	13_1	15	13	13	190	0	latdisplay
AxA	13_1	15	13	13	195	0	latdisplay
AxA	13_1	15	13	13	200	0	latdisplay
AxA	13_1	15	13	13	205	0	latdisplay
AxA	13_1	15	13	13	210	0	latdisplay
AxA	13_1	15	13	13	215	0	latdisplay
AxA	13_1	15	13	13	220	0	latdisplay
AxA	13_1	15	13	13	225	0	latdisplay
AxA	13_1	15	13	13	230	0	latdisplay
AxA	13_1	15	13	13	235	0	latdisplay

AxA	13_1	15	13	13	240	0	latdisplay
AxA	13_1	15	13	13	245	0	latdisplay
AxA	13_1	15	13	13	250	0	latdisplay
AxA	13_1	15	13	13	255	0	latdisplay
AxA	13_1	15	13	13	260	0	latdisplay
AxA	13_1	15	13	13	265	0	latdisplay
AxA	13_1	15	13	13	270	0	latdisplay
AxA	13_1	15	13	13	275	0	latdisplay
AxA	13_1	15	13	13	280	0	latdisplay
AxA	13_1	15	13	13	285	0	latdisplay
AxA	13_1	15	13	13	290	0	latdisplay
AxA	13_1	15	13	13	295	0	latdisplay
AxA	13_1	15	13	13	300	0	latdisplay
AxA	14_1	10	11	14	5	0	latdisplay
AxA	14_1	10	11	14	10	0	latdisplay
AxA	14_1	10	11	14	15	0	latdisplay
AxA	14_1	10	11	14	20	0	latdisplay
AxA	14_1	10	11	14	25	0	latdisplay
AxA	14_1	10	11	14	30	0	latdisplay
AxA	14_1	10	11	14	35	0	latdisplay
AxA	14_1	10	11	14	40	0	latdisplay
AxA	14_1	10	11	14	45	0	latdisplay
AxA	14_1	10	11	14	50	0	latdisplay
AxA	14_1	10	11	14	55	0	latdisplay
AxA	14_1	10	11	14	60	0	latdisplay
AxA	14_1	10	11	14	65	0	latdisplay
AxA	14_1	10	11	14	70	0	latdisplay
AxA	14_1	10	11	14	75	0	latdisplay
AxA	14_1	10	11	14	80	0	latdisplay
AxA	14_1	10	11	14	85	0	latdisplay
AxA	14_1	10	11	14	90	0	latdisplay
AxA	14_1	10	11	14	95	0	latdisplay
AxA	14_1	10	11	14	100	0	latdisplay
AxA	14_1	10	11	14	105	0	latdisplay
AxA	14_1	10	11	14	110	0	latdisplay
AxA	14_1	10	11	14	115	0	latdisplay
AxA	14_1	10	11	14	120	0	latdisplay
AxA	14_1	10	11	14	125	0	latdisplay
AxA	14_1	10	11	14	130	0	latdisplay
AxA	14_1	10	11	14	135	0	latdisplay
AxA	14_1	10	11	14	140	0	latdisplay
AxA	14_1	10	11	14	145	0	latdisplay
AxA	14_1	10	11	14	150	0	latdisplay
AxA	14_1	10	11	14	155	0	latdisplay
AxA	14_1	10	11	14	160	0	latdisplay
AxA	14_1	10	11	14	165	0	latdisplay
AxA	14_1	10	11	14	170	0	latdisplay
AxA	14_1	10	11	14	175	0	latdisplay

AxA	14_1	10	11	14	180	0	latdisplay
AxA	14_1	10	11	14	185	0	latdisplay
AxA	14_1	10	11	14	190	0	latdisplay
AxA	14_1	10	11	14	195	0	latdisplay
AxA	14_1	10	11	14	200	0	latdisplay
AxA	14_1	10	11	14	205	0	latdisplay
AxA	14_1	10	11	14	210	0	latdisplay
AxA	14_1	10	11	14	215	0	latdisplay
AxA	14_1	10	11	14	220	0	latdisplay
AxA	14_1	10	11	14	225	0	latdisplay
AxA	14_1	10	11	14	230	0	latdisplay
AxA	14_1	10	11	14	235	0	latdisplay
AxA	14_1	10	11	14	240	0	latdisplay
AxA	14_1	10	11	14	245	0	latdisplay
AxA	14_1	10	11	14	250	0	latdisplay
AxA	14_1	10	11	14	255	0	latdisplay
AxA	14_1	10	11	14	260	0	latdisplay
AxA	14_1	10	11	14	265	0	latdisplay
AxA	14_1	10	11	14	270	0	latdisplay
AxA	14_1	10	11	14	275	0	latdisplay
AxA	14_1	10	11	14	280	0	latdisplay
AxA	14_1	10	11	14	285	0	latdisplay
AxA	14_1	10	11	14	290	0	latdisplay
AxA	14_1	10	11	14	295	0	latdisplay
AxA	14_1	10	11	14	300	0	latdisplay
AxA	15_1	13	13	15	5	0	latdisplay
AxA	15_1	13	13	15	10	0	latdisplay
AxA	15_1	13	13	15	15	0	latdisplay
AxA	15_1	13	13	15	20	0	latdisplay
AxA	15_1	13	13	15	25	0	latdisplay
AxA	15_1	13	13	15	30	0	latdisplay
AxA	15_1	13	13	15	35	0	latdisplay
AxA	15_1	13	13	15	40	0	latdisplay
AxA	15_1	13	13	15	45	0	latdisplay
AxA	15_1	13	13	15	50	0	latdisplay
AxA	15_1	13	13	15	55	0	latdisplay
AxA	15_1	13	13	15	60	0	latdisplay
AxA	15_1	13	13	15	65	0	latdisplay
AxA	15_1	13	13	15	70	0	latdisplay
AxA	15_1	13	13	15	75	0	latdisplay
AxA	15_1	13	13	15	80	0	latdisplay
AxA	15_1	13	13	15	85	0	latdisplay
AxA	15_1	13	13	15	90	0	latdisplay
AxA	15_1	13	13	15	95	0	latdisplay
AxA	15_1	13	13	15	100	0	latdisplay
AxA	15_1	13	13	15	105	0	latdisplay
AxA	15_1	13	13	15	110	0	latdisplay
AxA	15_1	13	13	15	115	0	latdisplay

AxA	15_1	13	13	15	120	0	latdisplay
AxA	15_1	13	13	15	125	0	latdisplay
AxA	15_1	13	13	15	130	0	latdisplay
AxA	15_1	13	13	15	135	0	latdisplay
AxA	15_1	13	13	15	140	0	latdisplay
AxA	15_1	13	13	15	145	0	latdisplay
AxA	15_1	13	13	15	150	0	latdisplay
AxA	15_1	13	13	15	155	0	latdisplay
AxA	15_1	13	13	15	160	0	latdisplay
AxA	15_1	13	13	15	165	0	latdisplay
AxA	15_1	13	13	15	170	0	latdisplay
AxA	15_1	13	13	15	175	0	latdisplay
AxA	15_1	13	13	15	180	0	latdisplay
AxA	15_1	13	13	15	185	0	latdisplay
AxA	15_1	13	13	15	190	1	latdisplay
AxA	15_1	13	13	15	195	0	latdisplay
AxA	15_1	13	13	15	200	0	latdisplay
AxA	15_1	13	13	15	205	0	latdisplay
AxA	15_1	13	13	15	210	0	latdisplay
AxA	15_1	13	13	15	215	0	latdisplay
AxA	15_1	13	13	15	220	0	latdisplay
AxA	15_1	13	13	15	225	0	latdisplay
AxA	15_1	13	13	15	230	0	latdisplay
AxA	15_1	13	13	15	235	0	latdisplay
AxA	15_1	13	13	15	240	0	latdisplay
AxA	15_1	13	13	15	245	0	latdisplay
AxA	15_1	13	13	15	250	0	latdisplay
AxA	15_1	13	13	15	255	0	latdisplay
AxA	15_1	13	13	15	260	0	latdisplay
AxA	15_1	13	13	15	265	0	latdisplay
AxA	15_1	13	13	15	270	0	latdisplay
AxA	15_1	13	13	15	275	0	latdisplay
AxA	15_1	13	13	15	280	0	latdisplay
AxA	15_1	13	13	15	285	0	latdisplay
AxA	15_1	13	13	15	290	0	latdisplay
AxA	15_1	13	13	15	295	0	latdisplay
AxA	15_1	13	13	15	300	0	latdisplay
AxA	16_1	14	15	16	5	0	latdisplay
AxA	16_1	14	15	16	10	0	latdisplay
AxA	16_1	14	15	16	15	0	latdisplay
AxA	16_1	14	15	16	20	0	latdisplay
AxA	16_1	14	15	16	25	0	latdisplay
AxA	16_1	14	15	16	30	0	latdisplay
AxA	16_1	14	15	16	35	0	latdisplay
AxA	16_1	14	15	16	40	0	latdisplay
AxA	16_1	14	15	16	45	0	latdisplay
AxA	16_1	14	15	16	50	0	latdisplay
AxA	16_1	14	15	16	55	0	latdisplay

AxA	16_1	14	15	16	60	0	latdisplay
AxA	16_1	14	15	16	65	0	latdisplay
AxA	16_1	14	15	16	70	0	latdisplay
AxA	16_1	14	15	16	75	0	latdisplay
AxA	16_1	14	15	16	80	0	latdisplay
AxA	16_1	14	15	16	85	0	latdisplay
AxA	16_1	14	15	16	90	0	latdisplay
AxA	16_1	14	15	16	95	0	latdisplay
AxA	16_1	14	15	16	100	0	latdisplay
AxA	16_1	14	15	16	105	1	latdisplay
AxA	16_1	14	15	16	110	0	latdisplay
AxA	16_1	14	15	16	115	0	latdisplay
AxA	16_1	14	15	16	120	0	latdisplay
AxA	16_1	14	15	16	125	1	latdisplay
AxA	16_1	14	15	16	130	0	latdisplay
AxA	16_1	14	15	16	135	0	latdisplay
AxA	16_1	14	15	16	140	0	latdisplay
AxA	16_1	14	15	16	145	1	latdisplay
AxA	16_1	14	15	16	150	0	latdisplay
AxA	16_1	14	15	16	155	0	latdisplay
AxA	16_1	14	15	16	160	0	latdisplay
AxA	16_1	14	15	16	165	0	latdisplay
AxA	16_1	14	15	16	170	1	latdisplay
AxA	16_1	14	15	16	175	0	latdisplay
AxA	16_1	14	15	16	180	0	latdisplay
AxA	16_1	14	15	16	185	0	latdisplay
AxA	16_1	14	15	16	190	0	latdisplay
AxA	16_1	14	15	16	195	1	latdisplay
AxA	16_1	14	15	16	200	0	latdisplay
AxA	16_1	14	15	16	205	1	latdisplay
AxA	16_1	14	15	16	210	0	latdisplay
AxA	16_1	14	15	16	215	0	latdisplay
AxA	16_1	14	15	16	220	0	latdisplay
AxA	16_1	14	15	16	225	0	latdisplay
AxA	16_1	14	15	16	230	0	latdisplay
AxA	16_1	14	15	16	235	0	latdisplay
AxA	16_1	14	15	16	240	1	latdisplay
AxA	16_1	14	15	16	245	0	latdisplay
AxA	16_1	14	15	16	250	0	latdisplay
AxA	16_1	14	15	16	255	1	latdisplay
AxA	16_1	14	15	16	260	0	latdisplay
AxA	16_1	14	15	16	265	0	latdisplay
AxA	16_1	14	15	16	270	0	latdisplay
AxA	16_1	14	15	16	275	0	latdisplay
AxA	16_1	14	15	16	280	0	latdisplay
AxA	16_1	14	15	16	285	0	latdisplay
AxA	16_1	14	15	16	290	0	latdisplay
AxA	16_1	14	15	16	295	0	latdisplay

AxA	16_1	14	15	16	300	0	latdisplay
AxA	17_1	7	9	17	5	0	latdisplay
AxA	17_1	7	9	17	10	0	latdisplay
AxA	17_1	7	9	17	15	0	latdisplay
AxA	17_1	7	9	17	20	0	latdisplay
AxA	17_1	7	9	17	25	0	latdisplay
AxA	17_1	7	9	17	30	0	latdisplay
AxA	17_1	7	9	17	35	0	latdisplay
AxA	17_1	7	9	17	40	0	latdisplay
AxA	17_1	7	9	17	45	0	latdisplay
AxA	17_1	7	9	17	50	0	latdisplay
AxA	17_1	7	9	17	55	0	latdisplay
AxA	17_1	7	9	17	60	0	latdisplay
AxA	17_1	7	9	17	65	0	latdisplay
AxA	17_1	7	9	17	70	0	latdisplay
AxA	17_1	7	9	17	75	0	latdisplay
AxA	17_1	7	9	17	80	0	latdisplay
AxA	17_1	7	9	17	85	0	latdisplay
AxA	17_1	7	9	17	90	0	latdisplay
AxA	17_1	7	9	17	95	0	latdisplay
AxA	17_1	7	9	17	100	0	latdisplay
AxA	17_1	7	9	17	105	0	latdisplay
AxA	17_1	7	9	17	110	0	latdisplay
AxA	17_1	7	9	17	115	0	latdisplay
AxA	17_1	7	9	17	120	0	latdisplay
AxA	17_1	7	9	17	125	0	latdisplay
AxA	17_1	7	9	17	130	0	latdisplay
AxA	17_1	7	9	17	135	0	latdisplay
AxA	17_1	7	9	17	140	0	latdisplay
AxA	17_1	7	9	17	145	0	latdisplay
AxA	17_1	7	9	17	150	0	latdisplay
AxA	17_1	7	9	17	155	0	latdisplay
AxA	17_1	7	9	17	160	0	latdisplay
AxA	17_1	7	9	17	165	0	latdisplay
AxA	17_1	7	9	17	170	0	latdisplay
AxA	17_1	7	9	17	175	0	latdisplay
AxA	17_1	7	9	17	180	0	latdisplay
AxA	17_1	7	9	17	185	0	latdisplay
AxA	17_1	7	9	17	190	0	latdisplay
AxA	17_1	7	9	17	195	0	latdisplay
AxA	17_1	7	9	17	200	0	latdisplay
AxA	17_1	7	9	17	205	0	latdisplay
AxA	17_1	7	9	17	210	0	latdisplay
AxA	17_1	7	9	17	215	0	latdisplay
AxA	17_1	7	9	17	220	0	latdisplay
AxA	17_1	7	9	17	225	0	latdisplay
AxA	17_1	7	9	17	230	0	latdisplay
AxA	17_1	7	9	17	235	0	latdisplay

AxA	17_1	7	9	17	240	0	latdisplay
AxA	17_1	7	9	17	245	0	latdisplay
AxA	17_1	7	9	17	250	0	latdisplay
AxA	17_1	7	9	17	255	0	latdisplay
AxA	17_1	7	9	17	260	0	latdisplay
AxA	17_1	7	9	17	265	0	latdisplay
AxA	17_1	7	9	17	270	0	latdisplay
AxA	17_1	7	9	17	275	0	latdisplay
AxA	17_1	7	9	17	280	0	latdisplay
AxA	17_1	7	9	17	285	0	latdisplay
AxA	17_1	7	9	17	290	0	latdisplay
AxA	17_1	7	9	17	295	0	latdisplay
AxA	17_1	7	9	17	300	0	latdisplay
AxA	18_1	12	9	18	5	0	latdisplay
AxA	18_1	12	9	18	10	0	latdisplay
AxA	18_1	12	9	18	15	0	latdisplay
AxA	18_1	12	9	18	20	0	latdisplay
AxA	18_1	12	9	18	25	0	latdisplay
AxA	18_1	12	9	18	30	0	latdisplay
AxA	18_1	12	9	18	35	0	latdisplay
AxA	18_1	12	9	18	40	0	latdisplay
AxA	18_1	12	9	18	45	0	latdisplay
AxA	18_1	12	9	18	50	0	latdisplay
AxA	18_1	12	9	18	55	1	latdisplay
AxA	18_1	12	9	18	60	0	latdisplay
AxA	18_1	12	9	18	65	1	latdisplay
AxA	18_1	12	9	18	70	0	latdisplay
AxA	18_1	12	9	18	75	0	latdisplay
AxA	18_1	12	9	18	80	0	latdisplay
AxA	18_1	12	9	18	85	0	latdisplay
AxA	18_1	12	9	18	90	0	latdisplay
AxA	18_1	12	9	18	95	1	latdisplay
AxA	18_1	12	9	18	100	0	latdisplay
AxA	18_1	12	9	18	105	1	latdisplay
AxA	18_1	12	9	18	110	0	latdisplay
AxA	18_1	12	9	18	115	0	latdisplay
AxA	18_1	12	9	18	120	0	latdisplay
AxA	18_1	12	9	18	125	0	latdisplay
AxA	18_1	12	9	18	130	0	latdisplay
AxA	18_1	12	9	18	135	0	latdisplay
AxA	18_1	12	9	18	140	0	latdisplay
AxA	18_1	12	9	18	145	0	latdisplay
AxA	18_1	12	9	18	150	0	latdisplay
AxA	18_1	12	9	18	155	0	latdisplay
AxA	18_1	12	9	18	160	0	latdisplay
AxA	18_1	12	9	18	165	0	latdisplay
AxA	18_1	12	9	18	170	0	latdisplay
AxA	18_1	12	9	18	175	0	latdisplay

AxA	18_1	12	9	18	180	0	latdisplay
AxA	18_1	12	9	18	185	0	latdisplay
AxA	18_1	12	9	18	190	0	latdisplay
AxA	18_1	12	9	18	195	0	latdisplay
AxA	18_1	12	9	18	200	0	latdisplay
AxA	18_1	12	9	18	205	0	latdisplay
AxA	18_1	12	9	18	210	1	latdisplay
AxA	18_1	12	9	18	215	0	latdisplay
AxA	18_1	12	9	18	220	0	latdisplay
AxA	18_1	12	9	18	225	0	latdisplay
AxA	18_1	12	9	18	230	0	latdisplay
AxA	18_1	12	9	18	235	0	latdisplay
AxA	18_1	12	9	18	240	0	latdisplay
AxA	18_1	12	9	18	245	0	latdisplay
AxA	18_1	12	9	18	250	0	latdisplay
AxA	18_1	12	9	18	255	0	latdisplay
AxA	18_1	12	9	18	260	0	latdisplay
AxA	18_1	12	9	18	265	0	latdisplay
AxA	18_1	12	9	18	270	0	latdisplay
AxA	18_1	12	9	18	275	0	latdisplay
AxA	18_1	12	9	18	280	0	latdisplay
AxA	18_1	12	9	18	285	0	latdisplay
AxA	18_1	12	9	18	290	0	latdisplay
AxA	18_1	12	9	18	295	0	latdisplay
AxA	18_1	12	9	18	300	0	latdisplay
AxA	19_1	8	13	19	5	0	latdisplay
AxA	19_1	8	13	19	10	0	latdisplay
AxA	19_1	8	13	19	15	0	latdisplay
AxA	19_1	8	13	19	20	0	latdisplay
AxA	19_1	8	13	19	25	0	latdisplay
AxA	19_1	8	13	19	30	0	latdisplay
AxA	19_1	8	13	19	35	0	latdisplay
AxA	19_1	8	13	19	40	0	latdisplay
AxA	19_1	8	13	19	45	0	latdisplay
AxA	19_1	8	13	19	50	0	latdisplay
AxA	19_1	8	13	19	55	0	latdisplay
AxA	19_1	8	13	19	60	0	latdisplay
AxA	19_1	8	13	19	65	0	latdisplay
AxA	19_1	8	13	19	70	1	latdisplay
AxA	19_1	8	13	19	75	1	latdisplay
AxA	19_1	8	13	19	80	0	latdisplay
AxA	19_1	8	13	19	85	0	latdisplay
AxA	19_1	8	13	19	90	1	latdisplay
AxA	19_1	8	13	19	95	0	latdisplay
AxA	19_1	8	13	19	100	1	latdisplay
AxA	19_1	8	13	19	105	0	latdisplay
AxA	19_1	8	13	19	110	1	latdisplay
AxA	19_1	8	13	19	115	0	latdisplay

AxA	19_1	8	13	19	120	0	latdisplay
AxA	19_1	8	13	19	125	1	latdisplay
AxA	19_1	8	13	19	130	0	latdisplay
AxA	19_1	8	13	19	135	0	latdisplay
AxA	19_1	8	13	19	140	0	latdisplay
AxA	19_1	8	13	19	145	0	latdisplay
AxA	19_1	8	13	19	150	0	latdisplay
AxA	19_1	8	13	19	155	0	latdisplay
AxA	19_1	8	13	19	160	0	latdisplay
AxA	19_1	8	13	19	165	0	latdisplay
AxA	19_1	8	13	19	170	0	latdisplay
AxA	19_1	8	13	19	175	1	latdisplay
AxA	19_1	8	13	19	180	1	latdisplay
AxA	19_1	8	13	19	185	0	latdisplay
AxA	19_1	8	13	19	190	0	latdisplay
AxA	19_1	8	13	19	195	0	latdisplay
AxA	19_1	8	13	19	200	1	latdisplay
AxA	19_1	8	13	19	205	0	latdisplay
AxA	19_1	8	13	19	210	0	latdisplay
AxA	19_1	8	13	19	215	0	latdisplay
AxA	19_1	8	13	19	220	0	latdisplay
AxA	19_1	8	13	19	225	0	latdisplay
AxA	19_1	8	13	19	230	0	latdisplay
AxA	19_1	8	13	19	235	0	latdisplay
AxA	19_1	8	13	19	240	0	latdisplay
AxA	19_1	8	13	19	245	0	latdisplay
AxA	19_1	8	13	19	250	0	latdisplay
AxA	19_1	8	13	19	255	0	latdisplay
AxA	19_1	8	13	19	260	0	latdisplay
AxA	19_1	8	13	19	265	0	latdisplay
AxA	19_1	8	13	19	270	0	latdisplay
AxA	19_1	8	13	19	275	0	latdisplay
AxA	19_1	8	13	19	280	0	latdisplay
AxA	19_1	8	13	19	285	0	latdisplay
AxA	19_1	8	13	19	290	0	latdisplay
AxA	19_1	8	13	19	295	0	latdisplay
AxA	19_1	8	13	19	300	0	latdisplay
AxA	20_1	15	12	20	5	0	latdisplay
AxA	20_1	15	12	20	10	0	latdisplay
AxA	20_1	15	12	20	15	0	latdisplay
AxA	20_1	15	12	20	20	0	latdisplay
AxA	20_1	15	12	20	25	0	latdisplay
AxA	20_1	15	12	20	30	0	latdisplay
AxA	20_1	15	12	20	35	1	latdisplay
AxA	20_1	15	12	20	40	1	latdisplay
AxA	20_1	15	12	20	45	0	latdisplay
AxA	20_1	15	12	20	50	1	latdisplay
AxA	20_1	15	12	20	55	1	latdisplay

AxA	20_1	15	12	20	60	1	latdisplay
AxA	20_1	15	12	20	65	0	latdisplay
AxA	20_1	15	12	20	70	0	latdisplay
AxA	20_1	15	12	20	75	0	latdisplay
AxA	20_1	15	12	20	80	0	latdisplay
AxA	20_1	15	12	20	85	1	latdisplay
AxA	20_1	15	12	20	90	1	latdisplay
AxA	20_1	15	12	20	95	0	latdisplay
AxA	20_1	15	12	20	100	0	latdisplay
AxA	20_1	15	12	20	105	0	latdisplay
AxA	20_1	15	12	20	110	0	latdisplay
AxA	20_1	15	12	20	115	0	latdisplay
AxA	20_1	15	12	20	120	0	latdisplay
AxA	20_1	15	12	20	125	0	latdisplay
AxA	20_1	15	12	20	130	0	latdisplay
AxA	20_1	15	12	20	135	0	latdisplay
AxA	20_1	15	12	20	140	0	latdisplay
AxA	20_1	15	12	20	145	0	latdisplay
AxA	20_1	15	12	20	150	0	latdisplay
AxA	20_1	15	12	20	155	0	latdisplay
AxA	20_1	15	12	20	160	0	latdisplay
AxA	20_1	15	12	20	165	0	latdisplay
AxA	20_1	15	12	20	170	0	latdisplay
AxA	20_1	15	12	20	175	0	latdisplay
AxA	20_1	15	12	20	180	0	latdisplay
AxA	20_1	15	12	20	185	0	latdisplay
AxA	20_1	15	12	20	190	0	latdisplay
AxA	20_1	15	12	20	195	0	latdisplay
AxA	20_1	15	12	20	200	0	latdisplay
AxA	20_1	15	12	20	205	0	latdisplay
AxA	20_1	15	12	20	210	0	latdisplay
AxA	20_1	15	12	20	215	0	latdisplay
AxA	20_1	15	12	20	220	0	latdisplay
AxA	20_1	15	12	20	225	0	latdisplay
AxA	20_1	15	12	20	230	0	latdisplay
AxA	20_1	15	12	20	235	0	latdisplay
AxA	20_1	15	12	20	240	0	latdisplay
AxA	20_1	15	12	20	245	0	latdisplay
AxA	20_1	15	12	20	250	0	latdisplay
AxA	20_1	15	12	20	255	0	latdisplay
AxA	20_1	15	12	20	260	0	latdisplay
AxA	20_1	15	12	20	265	0	latdisplay
AxA	20_1	15	12	20	270	0	latdisplay
AxA	20_1	15	12	20	275	0	latdisplay
AxA	20_1	15	12	20	280	0	latdisplay
AxA	20_1	15	12	20	285	0	latdisplay
AxA	20_1	15	12	20	290	0	latdisplay
AxA	20_1	15	12	20	295	0	latdisplay

AxA	20_1	15	12	20	300	0	latdisplay
CxC	1_1	10	7	39	5	0	latdisplay
CxC	1_1	10	7	39	10	0	latdisplay
CxC	1_1	10	7	39	15	0	latdisplay
CxC	1_1	10	7	39	20	0	latdisplay
CxC	1_1	10	7	39	25	0	latdisplay
CxC	1_1	10	7	39	30	0	latdisplay
CxC	1_1	10	7	39	35	0	latdisplay
CxC	1_1	10	7	39	40	0	latdisplay
CxC	1_1	10	7	39	45	1	latdisplay
CxC	1_1	10	7	39	50	1	latdisplay
CxC	1_1	10	7	39	55	0	latdisplay
CxC	1_1	10	7	39	60	0	latdisplay
CxC	1_1	10	7	39	65	0	latdisplay
CxC	1_1	10	7	39	70	0	latdisplay
CxC	1_1	10	7	39	75	0	latdisplay
CxC	1_1	10	7	39	80	0	latdisplay
CxC	1_1	10	7	39	85	0	latdisplay
CxC	1_1	10	7	39	90	0	latdisplay
CxC	1_1	10	7	39	95	0	latdisplay
CxC	1_1	10	7	39	100	0	latdisplay
CxC	1_1	10	7	39	105	0	latdisplay
CxC	1_1	10	7	39	110	0	latdisplay
CxC	1_1	10	7	39	115	0	latdisplay
CxC	1_1	10	7	39	120	0	latdisplay
CxC	1_1	10	7	39	125	0	latdisplay
CxC	1_1	10	7	39	130	0	latdisplay
CxC	1_1	10	7	39	135	0	latdisplay
CxC	1_1	10	7	39	140	0	latdisplay
CxC	1_1	10	7	39	145	0	latdisplay
CxC	1_1	10	7	39	150	0	latdisplay
CxC	1_1	10	7	39	155	0	latdisplay
CxC	1_1	10	7	39	160	0	latdisplay
CxC	1_1	10	7	39	165	0	latdisplay
CxC	1_1	10	7	39	170	0	latdisplay
CxC	1_1	10	7	39	175	0	latdisplay
CxC	1_1	10	7	39	180	0	latdisplay
CxC	1_1	10	7	39	185	0	latdisplay
CxC	1_1	10	7	39	190	0	latdisplay
CxC	1_1	10	7	39	195	0	latdisplay
CxC	1_1	10	7	39	200	1	latdisplay
CxC	1_1	10	7	39	205	1	latdisplay
CxC	1_1	10	7	39	210	0	latdisplay
CxC	1_1	10	7	39	215	0	latdisplay
CxC	1_1	10	7	39	220	1	latdisplay
CxC	1_1	10	7	39	225	0	latdisplay
CxC	1_1	10	7	39	230	0	latdisplay
CxC	1_1	10	7	39	235	0	latdisplay

CxC	1_1	10	7	39	240	0	latdisplay
CxC	1_1	10	7	39	245	0	latdisplay
CxC	1_1	10	7	39	250	1	latdisplay
CxC	1_1	10	7	39	255	1	latdisplay
CxC	1_1	10	7	39	260	0	latdisplay
CxC	1_1	10	7	39	265	1	latdisplay
CxC	1_1	10	7	39	270	0	latdisplay
CxC	1_1	10	7	39	275	0	latdisplay
CxC	1_1	10	7	39	280	0	latdisplay
CxC	1_1	10	7	39	285	0	latdisplay
CxC	1_1	10	7	39	290	1	latdisplay
CxC	1_1	10	7	39	295	0	latdisplay
CxC	1_1	10	7	39	300	0	latdisplay
CxC	2_1	9	10	40	5	0	latdisplay
CxC	2_1	9	10	40	10	0	latdisplay
CxC	2_1	9	10	40	15	0	latdisplay
CxC	2_1	9	10	40	20	0	latdisplay
CxC	2_1	9	10	40	25	0	latdisplay
CxC	2_1	9	10	40	30	0	latdisplay
CxC	2_1	9	10	40	35	0	latdisplay
CxC	2_1	9	10	40	40	0	latdisplay
CxC	2_1	9	10	40	45	0	latdisplay
CxC	2_1	9	10	40	50	0	latdisplay
CxC	2_1	9	10	40	55	1	latdisplay
CxC	2_1	9	10	40	60	1	latdisplay
CxC	2_1	9	10	40	65	0	latdisplay
CxC	2_1	9	10	40	70	1	latdisplay
CxC	2_1	9	10	40	75	1	latdisplay
CxC	2_1	9	10	40	80	0	latdisplay
CxC	2_1	9	10	40	85	0	latdisplay
CxC	2_1	9	10	40	90	0	latdisplay
CxC	2_1	9	10	40	95	0	latdisplay
CxC	2_1	9	10	40	100	0	latdisplay
CxC	2_1	9	10	40	105	0	latdisplay
CxC	2_1	9	10	40	110	0	latdisplay
CxC	2_1	9	10	40	115	1	latdisplay
CxC	2_1	9	10	40	120	1	latdisplay
CxC	2_1	9	10	40	125	0	latdisplay
CxC	2_1	9	10	40	130	1	latdisplay
CxC	2_1	9	10	40	135	1	latdisplay
CxC	2_1	9	10	40	140	0	latdisplay
CxC	2_1	9	10	40	145	1	latdisplay
CxC	2_1	9	10	40	150	0	latdisplay
CxC	2_1	9	10	40	155	0	latdisplay
CxC	2_1	9	10	40	160	0	latdisplay
CxC	2_1	9	10	40	165	0	latdisplay
CxC	2_1	9	10	40	170	0	latdisplay
CxC	2_1	9	10	40	175	0	latdisplay

CxC	2_1	9	10	40	180	0	latdisplay
CxC	2_1	9	10	40	185	1	latdisplay
CxC	2_1	9	10	40	190	1	latdisplay
CxC	2_1	9	10	40	195	0	latdisplay
CxC	2_1	9	10	40	200	0	latdisplay
CxC	2_1	9	10	40	205	1	latdisplay
CxC	2_1	9	10	40	210	1	latdisplay
CxC	2_1	9	10	40	215	1	latdisplay
CxC	2_1	9	10	40	220	0	latdisplay
CxC	2_1	9	10	40	225	1	latdisplay
CxC	2_1	9	10	40	230	0	latdisplay
CxC	2_1	9	10	40	235	0	latdisplay
CxC	2_1	9	10	40	240	0	latdisplay
CxC	2_1	9	10	40	245	1	latdisplay
CxC	2_1	9	10	40	250	0	latdisplay
CxC	2_1	9	10	40	255	1	latdisplay
CxC	2_1	9	10	40	260	0	latdisplay
CxC	2_1	9	10	40	265	0	latdisplay
CxC	2_1	9	10	40	270	0	latdisplay
CxC	2_1	9	10	40	275	1	latdisplay
CxC	2_1	9	10	40	280	0	latdisplay
CxC	2_1	9	10	40	285	0	latdisplay
CxC	2_1	9	10	40	290	1	latdisplay
CxC	2_1	9	10	40	295	0	latdisplay
CxC	2_1	9	10	40	300	0	latdisplay
CxC	3_1	7	8	41	5	0	latdisplay
CxC	3_1	7	8	41	10	0	latdisplay
CxC	3_1	7	8	41	15	0	latdisplay
CxC	3_1	7	8	41	20	0	latdisplay
CxC	3_1	7	8	41	25	0	latdisplay
CxC	3_1	7	8	41	30	0	latdisplay
CxC	3_1	7	8	41	35	0	latdisplay
CxC	3_1	7	8	41	40	0	latdisplay
CxC	3_1	7	8	41	45	0	latdisplay
CxC	3_1	7	8	41	50	0	latdisplay
CxC	3_1	7	8	41	55	0	latdisplay
CxC	3_1	7	8	41	60	0	latdisplay
CxC	3_1	7	8	41	65	0	latdisplay
CxC	3_1	7	8	41	70	0	latdisplay
CxC	3_1	7	8	41	75	0	latdisplay
CxC	3_1	7	8	41	80	1	latdisplay
CxC	3_1	7	8	41	85	1	latdisplay
CxC	3_1	7	8	41	90	0	latdisplay
CxC	3_1	7	8	41	95	0	latdisplay
CxC	3_1	7	8	41	100	0	latdisplay
CxC	3_1	7	8	41	105	0	latdisplay
CxC	3_1	7	8	41	110	0	latdisplay
CxC	3_1	7	8	41	115	0	latdisplay

CxC	3_1	7	8	41	120	0	latdisplay
CxC	3_1	7	8	41	125	0	latdisplay
CxC	3_1	7	8	41	130	0	latdisplay
CxC	3_1	7	8	41	135	0	latdisplay
CxC	3_1	7	8	41	140	0	latdisplay
CxC	3_1	7	8	41	145	0	latdisplay
CxC	3_1	7	8	41	150	1	latdisplay
CxC	3_1	7	8	41	155	0	latdisplay
CxC	3_1	7	8	41	160	1	latdisplay
CxC	3_1	7	8	41	165	0	latdisplay
CxC	3_1	7	8	41	170	0	latdisplay
CxC	3_1	7	8	41	175	1	latdisplay
CxC	3_1	7	8	41	180	0	latdisplay
CxC	3_1	7	8	41	185	0	latdisplay
CxC	3_1	7	8	41	190	1	latdisplay
CxC	3_1	7	8	41	195	0	latdisplay
CxC	3_1	7	8	41	200	0	latdisplay
CxC	3_1	7	8	41	205	0	latdisplay
CxC	3_1	7	8	41	210	0	latdisplay
CxC	3_1	7	8	41	215	0	latdisplay
CxC	3_1	7	8	41	220	0	latdisplay
CxC	3_1	7	8	41	225	1	latdisplay
CxC	3_1	7	8	41	230	1	latdisplay
CxC	3_1	7	8	41	235	0	latdisplay
CxC	3_1	7	8	41	240	0	latdisplay
CxC	3_1	7	8	41	245	0	latdisplay
CxC	3_1	7	8	41	250	0	latdisplay
CxC	3_1	7	8	41	255	0	latdisplay
CxC	3_1	7	8	41	260	0	latdisplay
CxC	3_1	7	8	41	265	0	latdisplay
CxC	3_1	7	8	41	270	0	latdisplay
CxC	3_1	7	8	41	275	0	latdisplay
CxC	3_1	7	8	41	280	0	latdisplay
CxC	3_1	7	8	41	285	0	latdisplay
CxC	3_1	7	8	41	290	0	latdisplay
CxC	3_1	7	8	41	295	0	latdisplay
CxC	3_1	7	8	41	300	0	latdisplay
CxC	4_1	9	8	42	5	0	latdisplay
CxC	4_1	9	8	42	10	0	latdisplay
CxC	4_1	9	8	42	15	0	latdisplay
CxC	4_1	9	8	42	20	0	latdisplay
CxC	4_1	9	8	42	25	0	latdisplay
CxC	4_1	9	8	42	30	0	latdisplay
CxC	4_1	9	8	42	35	0	latdisplay
CxC	4_1	9	8	42	40	0	latdisplay
CxC	4_1	9	8	42	45	0	latdisplay
CxC	4_1	9	8	42	50	0	latdisplay
CxC	4_1	9	8	42	55	0	latdisplay

CxC	4_1	9	8	42	60	0	latdisplay
CxC	4_1	9	8	42	65	1	latdisplay
CxC	4_1	9	8	42	70	1	latdisplay
CxC	4_1	9	8	42	75	1	latdisplay
CxC	4_1	9	8	42	80	0	latdisplay
CxC	4_1	9	8	42	85	1	latdisplay
CxC	4_1	9	8	42	90	1	latdisplay
CxC	4_1	9	8	42	95	1	latdisplay
CxC	4_1	9	8	42	100	1	latdisplay
CxC	4_1	9	8	42	105	1	latdisplay
CxC	4_1	9	8	42	110	1	latdisplay
CxC	4_1	9	8	42	115	1	latdisplay
CxC	4_1	9	8	42	120	1	latdisplay
CxC	4_1	9	8	42	125	0	latdisplay
CxC	4_1	9	8	42	130	0	latdisplay
CxC	4_1	9	8	42	135	0	latdisplay
CxC	4_1	9	8	42	140	1	latdisplay
CxC	4_1	9	8	42	145	1	latdisplay
CxC	4_1	9	8	42	150	0	latdisplay
CxC	4_1	9	8	42	155	1	latdisplay
CxC	4_1	9	8	42	160	1	latdisplay
CxC	4_1	9	8	42	165	1	latdisplay
CxC	4_1	9	8	42	170	1	latdisplay
CxC	4_1	9	8	42	175	1	latdisplay
CxC	4_1	9	8	42	180	0	latdisplay
CxC	4_1	9	8	42	185	0	latdisplay
CxC	4_1	9	8	42	190	0	latdisplay
CxC	4_1	9	8	42	195	1	latdisplay
CxC	4_1	9	8	42	200	1	latdisplay
CxC	4_1	9	8	42	205	0	latdisplay
CxC	4_1	9	8	42	210	1	latdisplay
CxC	4_1	9	8	42	215	0	latdisplay
CxC	4_1	9	8	42	220	0	latdisplay
CxC	4_1	9	8	42	225	0	latdisplay
CxC	4_1	9	8	42	230	1	latdisplay
CxC	4_1	9	8	42	235	0	latdisplay
CxC	4_1	9	8	42	240	0	latdisplay
CxC	4_1	9	8	42	245	0	latdisplay
CxC	4_1	9	8	42	250	0	latdisplay
CxC	4_1	9	8	42	255	0	latdisplay
CxC	4_1	9	8	42	260	0	latdisplay
CxC	4_1	9	8	42	265	0	latdisplay
CxC	4_1	9	8	42	270	0	latdisplay
CxC	4_1	9	8	42	275	0	latdisplay
CxC	4_1	9	8	42	280	0	latdisplay
CxC	4_1	9	8	42	285	1	latdisplay
CxC	4_1	9	8	42	290	0	latdisplay
CxC	4_1	9	8	42	295	1	latdisplay

CxC	4_1	9	8	42	300	1	latdisplay
CxC	5_1	8	8	43	5	0	latdisplay
CxC	5_1	8	8	43	10	0	latdisplay
CxC	5_1	8	8	43	15	0	latdisplay
CxC	5_1	8	8	43	20	0	latdisplay
CxC	5_1	8	8	43	25	0	latdisplay
CxC	5_1	8	8	43	30	0	latdisplay
CxC	5_1	8	8	43	35	0	latdisplay
CxC	5_1	8	8	43	40	1	latdisplay
CxC	5_1	8	8	43	45	1	latdisplay
CxC	5_1	8	8	43	50	0	latdisplay
CxC	5_1	8	8	43	55	0	latdisplay
CxC	5_1	8	8	43	60	0	latdisplay
CxC	5_1	8	8	43	65	1	latdisplay
CxC	5_1	8	8	43	70	0	latdisplay
CxC	5_1	8	8	43	75	1	latdisplay
CxC	5_1	8	8	43	80	0	latdisplay
CxC	5_1	8	8	43	85	1	latdisplay
CxC	5_1	8	8	43	90	0	latdisplay
CxC	5_1	8	8	43	95	1	latdisplay
CxC	5_1	8	8	43	100	0	latdisplay
CxC	5_1	8	8	43	105	0	latdisplay
CxC	5_1	8	8	43	110	1	latdisplay
CxC	5_1	8	8	43	115	0	latdisplay
CxC	5_1	8	8	43	120	0	latdisplay
CxC	5_1	8	8	43	125	0	latdisplay
CxC	5_1	8	8	43	130	1	latdisplay
CxC	5_1	8	8	43	135	0	latdisplay
CxC	5_1	8	8	43	140	1	latdisplay
CxC	5_1	8	8	43	145	0	latdisplay
CxC	5_1	8	8	43	150	0	latdisplay
CxC	5_1	8	8	43	155	0	latdisplay
CxC	5_1	8	8	43	160	0	latdisplay
CxC	5_1	8	8	43	165	0	latdisplay
CxC	5_1	8	8	43	170	0	latdisplay
CxC	5_1	8	8	43	175	0	latdisplay
CxC	5_1	8	8	43	180	0	latdisplay
CxC	5_1	8	8	43	185	0	latdisplay
CxC	5_1	8	8	43	190	1	latdisplay
CxC	5_1	8	8	43	195	1	latdisplay
CxC	5_1	8	8	43	200	0	latdisplay
CxC	5_1	8	8	43	205	0	latdisplay
CxC	5_1	8	8	43	210	0	latdisplay
CxC	5_1	8	8	43	215	1	latdisplay
CxC	5_1	8	8	43	220	1	latdisplay
CxC	5_1	8	8	43	225	1	latdisplay
CxC	5_1	8	8	43	230	0	latdisplay
CxC	5_1	8	8	43	235	0	latdisplay

CxC	5_1	8	8	43	240	0	latdisplay
CxC	5_1	8	8	43	245	1	latdisplay
CxC	5_1	8	8	43	250	0	latdisplay
CxC	5_1	8	8	43	255	1	latdisplay
CxC	5_1	8	8	43	260	1	latdisplay
CxC	5_1	8	8	43	265	1	latdisplay
CxC	5_1	8	8	43	270	0	latdisplay
CxC	5_1	8	8	43	275	1	latdisplay
CxC	5_1	8	8	43	280	0	latdisplay
CxC	5_1	8	8	43	285	1	latdisplay
CxC	5_1	8	8	43	290	0	latdisplay
CxC	5_1	8	8	43	295	1	latdisplay
CxC	5_1	8	8	43	300	0	latdisplay
CxC	6_1	8	9	44	5	0	latdisplay
CxC	6_1	8	9	44	10	0	latdisplay
CxC	6_1	8	9	44	15	0	latdisplay
CxC	6_1	8	9	44	20	0	latdisplay
CxC	6_1	8	9	44	25	0	latdisplay
CxC	6_1	8	9	44	30	0	latdisplay
CxC	6_1	8	9	44	35	0	latdisplay
CxC	6_1	8	9	44	40	0	latdisplay
CxC	6_1	8	9	44	45	0	latdisplay
CxC	6_1	8	9	44	50	0	latdisplay
CxC	6_1	8	9	44	55	0	latdisplay
CxC	6_1	8	9	44	60	0	latdisplay
CxC	6_1	8	9	44	65	0	latdisplay
CxC	6_1	8	9	44	70	0	latdisplay
CxC	6_1	8	9	44	75	0	latdisplay
CxC	6_1	8	9	44	80	0	latdisplay
CxC	6_1	8	9	44	85	0	latdisplay
CxC	6_1	8	9	44	90	0	latdisplay
CxC	6_1	8	9	44	95	0	latdisplay
CxC	6_1	8	9	44	100	0	latdisplay
CxC	6_1	8	9	44	105	1	latdisplay
CxC	6_1	8	9	44	110	0	latdisplay
CxC	6_1	8	9	44	115	1	latdisplay
CxC	6_1	8	9	44	120	0	latdisplay
CxC	6_1	8	9	44	125	0	latdisplay
CxC	6_1	8	9	44	130	0	latdisplay
CxC	6_1	8	9	44	135	0	latdisplay
CxC	6_1	8	9	44	140	1	latdisplay
CxC	6_1	8	9	44	145	0	latdisplay
CxC	6_1	8	9	44	150	0	latdisplay
CxC	6_1	8	9	44	155	0	latdisplay
CxC	6_1	8	9	44	160	0	latdisplay
CxC	6_1	8	9	44	165	0	latdisplay
CxC	6_1	8	9	44	170	0	latdisplay
CxC	6_1	8	9	44	175	0	latdisplay

CxC	6_1	8	9	44	180	0	latdisplay
CxC	6_1	8	9	44	185	0	latdisplay
CxC	6_1	8	9	44	190	0	latdisplay
CxC	6_1	8	9	44	195	1	latdisplay
CxC	6_1	8	9	44	200	0	latdisplay
CxC	6_1	8	9	44	205	0	latdisplay
CxC	6_1	8	9	44	210	1	latdisplay
CxC	6_1	8	9	44	215	0	latdisplay
CxC	6_1	8	9	44	220	1	latdisplay
CxC	6_1	8	9	44	225	1	latdisplay
CxC	6_1	8	9	44	230	0	latdisplay
CxC	6_1	8	9	44	235	0	latdisplay
CxC	6_1	8	9	44	240	0	latdisplay
CxC	6_1	8	9	44	245	0	latdisplay
CxC	6_1	8	9	44	250	0	latdisplay
CxC	6_1	8	9	44	255	0	latdisplay
CxC	6_1	8	9	44	260	0	latdisplay
CxC	6_1	8	9	44	265	0	latdisplay
CxC	6_1	8	9	44	270	0	latdisplay
CxC	6_1	8	9	44	275	0	latdisplay
CxC	6_1	8	9	44	280	0	latdisplay
CxC	6_1	8	9	44	285	0	latdisplay
CxC	6_1	8	9	44	290	0	latdisplay
CxC	6_1	8	9	44	295	0	latdisplay
CxC	6_1	8	9	44	300	0	latdisplay
CxC	7_1	7	6	45	5	0	latdisplay
CxC	7_1	7	6	45	10	0	latdisplay
CxC	7_1	7	6	45	15	0	latdisplay
CxC	7_1	7	6	45	20	0	latdisplay
CxC	7_1	7	6	45	25	0	latdisplay
CxC	7_1	7	6	45	30	0	latdisplay
CxC	7_1	7	6	45	35	0	latdisplay
CxC	7_1	7	6	45	40	0	latdisplay
CxC	7_1	7	6	45	45	0	latdisplay
CxC	7_1	7	6	45	50	0	latdisplay
CxC	7_1	7	6	45	55	0	latdisplay
CxC	7_1	7	6	45	60	0	latdisplay
CxC	7_1	7	6	45	65	1	latdisplay
CxC	7_1	7	6	45	70	1	latdisplay
CxC	7_1	7	6	45	75	0	latdisplay
CxC	7_1	7	6	45	80	0	latdisplay
CxC	7_1	7	6	45	85	1	latdisplay
CxC	7_1	7	6	45	90	1	latdisplay
CxC	7_1	7	6	45	95	0	latdisplay
CxC	7_1	7	6	45	100	1	latdisplay
CxC	7_1	7	6	45	105	0	latdisplay
CxC	7_1	7	6	45	110	0	latdisplay
CxC	7_1	7	6	45	115	1	latdisplay

CxC	7_1	7	6	45	120	1	latdisplay
CxC	7_1	7	6	45	125	1	latdisplay
CxC	7_1	7	6	45	130	0	latdisplay
CxC	7_1	7	6	45	135	0	latdisplay
CxC	7_1	7	6	45	140	0	latdisplay
CxC	7_1	7	6	45	145	1	latdisplay
CxC	7_1	7	6	45	150	1	latdisplay
CxC	7_1	7	6	45	155	0	latdisplay
CxC	7_1	7	6	45	160	0	latdisplay
CxC	7_1	7	6	45	165	1	latdisplay
CxC	7_1	7	6	45	170	1	latdisplay
CxC	7_1	7	6	45	175	1	latdisplay
CxC	7_1	7	6	45	180	1	latdisplay
CxC	7_1	7	6	45	185	1	latdisplay
CxC	7_1	7	6	45	190	0	latdisplay
CxC	7_1	7	6	45	195	1	latdisplay
CxC	7_1	7	6	45	200	0	latdisplay
CxC	7_1	7	6	45	205	0	latdisplay
CxC	7_1	7	6	45	210	0	latdisplay
CxC	7_1	7	6	45	215	0	latdisplay
CxC	7_1	7	6	45	220	0	latdisplay
CxC	7_1	7	6	45	225	0	latdisplay
CxC	7_1	7	6	45	230	0	latdisplay
CxC	7_1	7	6	45	235	0	latdisplay
CxC	7_1	7	6	45	240	0	latdisplay
CxC	7_1	7	6	45	245	0	latdisplay
CxC	7_1	7	6	45	250	0	latdisplay
CxC	7_1	7	6	45	255	0	latdisplay
CxC	7_1	7	6	45	260	0	latdisplay
CxC	7_1	7	6	45	265	0	latdisplay
CxC	7_1	7	6	45	270	0	latdisplay
CxC	7_1	7	6	45	275	0	latdisplay
CxC	7_1	7	6	45	280	0	latdisplay
CxC	7_1	7	6	45	285	0	latdisplay
CxC	7_1	7	6	45	290	0	latdisplay
CxC	7_1	7	6	45	295	0	latdisplay
CxC	7_1	7	6	45	300	0	latdisplay
CxC	8_1	7	8	46	5	0	latdisplay
CxC	8_1	7	8	46	10	0	latdisplay
CxC	8_1	7	8	46	15	1	latdisplay
CxC	8_1	7	8	46	20	1	latdisplay
CxC	8_1	7	8	46	25	0	latdisplay
CxC	8_1	7	8	46	30	1	latdisplay
CxC	8_1	7	8	46	35	1	latdisplay
CxC	8_1	7	8	46	40	0	latdisplay
CxC	8_1	7	8	46	45	1	latdisplay
CxC	8_1	7	8	46	50	1	latdisplay
CxC	8_1	7	8	46	55	0	latdisplay

CxC	8_1	7	8	46	60	1	latdisplay
CxC	8_1	7	8	46	65	0	latdisplay
CxC	8_1	7	8	46	70	1	latdisplay
CxC	8_1	7	8	46	75	1	latdisplay
CxC	8_1	7	8	46	80	0	latdisplay
CxC	8_1	7	8	46	85	1	latdisplay
CxC	8_1	7	8	46	90	0	latdisplay
CxC	8_1	7	8	46	95	0	latdisplay
CxC	8_1	7	8	46	100	0	latdisplay
CxC	8_1	7	8	46	105	0	latdisplay
CxC	8_1	7	8	46	110	0	latdisplay
CxC	8_1	7	8	46	115	0	latdisplay
CxC	8_1	7	8	46	120	0	latdisplay
CxC	8_1	7	8	46	125	0	latdisplay
CxC	8_1	7	8	46	130	0	latdisplay
CxC	8_1	7	8	46	135	0	latdisplay
CxC	8_1	7	8	46	140	0	latdisplay
CxC	8_1	7	8	46	145	0	latdisplay
CxC	8_1	7	8	46	150	0	latdisplay
CxC	8_1	7	8	46	155	0	latdisplay
CxC	8_1	7	8	46	160	1	latdisplay
CxC	8_1	7	8	46	165	0	latdisplay
CxC	8_1	7	8	46	170	0	latdisplay
CxC	8_1	7	8	46	175	0	latdisplay
CxC	8_1	7	8	46	180	0	latdisplay
CxC	8_1	7	8	46	185	0	latdisplay
CxC	8_1	7	8	46	190	1	latdisplay
CxC	8_1	7	8	46	195	0	latdisplay
CxC	8_1	7	8	46	200	0	latdisplay
CxC	8_1	7	8	46	205	0	latdisplay
CxC	8_1	7	8	46	210	0	latdisplay
CxC	8_1	7	8	46	215	0	latdisplay
CxC	8_1	7	8	46	220	1	latdisplay
CxC	8_1	7	8	46	225	1	latdisplay
CxC	8_1	7	8	46	230	0	latdisplay
CxC	8_1	7	8	46	235	0	latdisplay
CxC	8_1	7	8	46	240	0	latdisplay
CxC	8_1	7	8	46	245	0	latdisplay
CxC	8_1	7	8	46	250	0	latdisplay
CxC	8_1	7	8	46	255	0	latdisplay
CxC	8_1	7	8	46	260	0	latdisplay
CxC	8_1	7	8	46	265	1	latdisplay
CxC	8_1	7	8	46	270	1	latdisplay
CxC	8_1	7	8	46	275	0	latdisplay
CxC	8_1	7	8	46	280	1	latdisplay
CxC	8_1	7	8	46	285	0	latdisplay
CxC	8_1	7	8	46	290	1	latdisplay
CxC	8_1	7	8	46	295	1	latdisplay

CxC	8_1	7	8	46	300	0	latdisplay
CxC	9_1	9	7	47	5	0	latdisplay
CxC	9_1	9	7	47	10	0	latdisplay
CxC	9_1	9	7	47	15	0	latdisplay
CxC	9_1	9	7	47	20	0	latdisplay
CxC	9_1	9	7	47	25	0	latdisplay
CxC	9_1	9	7	47	30	0	latdisplay
CxC	9_1	9	7	47	35	0	latdisplay
CxC	9_1	9	7	47	40	0	latdisplay
CxC	9_1	9	7	47	45	0	latdisplay
CxC	9_1	9	7	47	50	0	latdisplay
CxC	9_1	9	7	47	55	0	latdisplay
CxC	9_1	9	7	47	60	0	latdisplay
CxC	9_1	9	7	47	65	0	latdisplay
CxC	9_1	9	7	47	70	0	latdisplay
CxC	9_1	9	7	47	75	0	latdisplay
CxC	9_1	9	7	47	80	0	latdisplay
CxC	9_1	9	7	47	85	0	latdisplay
CxC	9_1	9	7	47	90	0	latdisplay
CxC	9_1	9	7	47	95	1	latdisplay
CxC	9_1	9	7	47	100	0	latdisplay
CxC	9_1	9	7	47	105	0	latdisplay
CxC	9_1	9	7	47	110	0	latdisplay
CxC	9_1	9	7	47	115	0	latdisplay
CxC	9_1	9	7	47	120	0	latdisplay
CxC	9_1	9	7	47	125	0	latdisplay
CxC	9_1	9	7	47	130	0	latdisplay
CxC	9_1	9	7	47	135	0	latdisplay
CxC	9_1	9	7	47	140	0	latdisplay
CxC	9_1	9	7	47	145	0	latdisplay
CxC	9_1	9	7	47	150	0	latdisplay
CxC	9_1	9	7	47	155	0	latdisplay
CxC	9_1	9	7	47	160	0	latdisplay
CxC	9_1	9	7	47	165	0	latdisplay
CxC	9_1	9	7	47	170	0	latdisplay
CxC	9_1	9	7	47	175	0	latdisplay
CxC	9_1	9	7	47	180	0	latdisplay
CxC	9_1	9	7	47	185	0	latdisplay
CxC	9_1	9	7	47	190	0	latdisplay
CxC	9_1	9	7	47	195	0	latdisplay
CxC	9_1	9	7	47	200	0	latdisplay
CxC	9_1	9	7	47	205	0	latdisplay
CxC	9_1	9	7	47	210	0	latdisplay
CxC	9_1	9	7	47	215	0	latdisplay
CxC	9_1	9	7	47	220	0	latdisplay
CxC	9_1	9	7	47	225	0	latdisplay
CxC	9_1	9	7	47	230	0	latdisplay
CxC	9_1	9	7	47	235	0	latdisplay

CxC	9_1	9	7	47	240	0	latdisplay
CxC	9_1	9	7	47	245	0	latdisplay
CxC	9_1	9	7	47	250	0	latdisplay
CxC	9_1	9	7	47	255	0	latdisplay
CxC	9_1	9	7	47	260	0	latdisplay
CxC	9_1	9	7	47	265	0	latdisplay
CxC	9_1	9	7	47	270	0	latdisplay
CxC	9_1	9	7	47	275	0	latdisplay
CxC	9_1	9	7	47	280	0	latdisplay
CxC	9_1	9	7	47	285	0	latdisplay
CxC	9_1	9	7	47	290	0	latdisplay
CxC	9_1	9	7	47	295	0	latdisplay
CxC	9_1	9	7	47	300	0	latdisplay
CxC	10_1	8	8	48	5	0	latdisplay
CxC	10_1	8	8	48	10	0	latdisplay
CxC	10_1	8	8	48	15	0	latdisplay
CxC	10_1	8	8	48	20	0	latdisplay
CxC	10_1	8	8	48	25	0	latdisplay
CxC	10_1	8	8	48	30	0	latdisplay
CxC	10_1	8	8	48	35	0	latdisplay
CxC	10_1	8	8	48	40	0	latdisplay
CxC	10_1	8	8	48	45	0	latdisplay
CxC	10_1	8	8	48	50	0	latdisplay
CxC	10_1	8	8	48	55	0	latdisplay
CxC	10_1	8	8	48	60	0	latdisplay
CxC	10_1	8	8	48	65	0	latdisplay
CxC	10_1	8	8	48	70	0	latdisplay
CxC	10_1	8	8	48	75	0	latdisplay
CxC	10_1	8	8	48	80	0	latdisplay
CxC	10_1	8	8	48	85	0	latdisplay
CxC	10_1	8	8	48	90	0	latdisplay
CxC	10_1	8	8	48	95	0	latdisplay
CxC	10_1	8	8	48	100	0	latdisplay
CxC	10_1	8	8	48	105	0	latdisplay
CxC	10_1	8	8	48	110	0	latdisplay
CxC	10_1	8	8	48	115	0	latdisplay
CxC	10_1	8	8	48	120	0	latdisplay
CxC	10_1	8	8	48	125	0	latdisplay
CxC	10_1	8	8	48	130	0	latdisplay
CxC	10_1	8	8	48	135	0	latdisplay
CxC	10_1	8	8	48	140	0	latdisplay
CxC	10_1	8	8	48	145	0	latdisplay
CxC	10_1	8	8	48	150	0	latdisplay
CxC	10_1	8	8	48	155	0	latdisplay
CxC	10_1	8	8	48	160	0	latdisplay
CxC	10_1	8	8	48	165	0	latdisplay
CxC	10_1	8	8	48	170	0	latdisplay
CxC	10_1	8	8	48	175	0	latdisplay

CxC	10_1	8	8	48	180	0	latdisplay
CxC	10_1	8	8	48	185	0	latdisplay
CxC	10_1	8	8	48	190	0	latdisplay
CxC	10_1	8	8	48	195	0	latdisplay
CxC	10_1	8	8	48	200	0	latdisplay
CxC	10_1	8	8	48	205	0	latdisplay
CxC	10_1	8	8	48	210	0	latdisplay
CxC	10_1	8	8	48	215	0	latdisplay
CxC	10_1	8	8	48	220	0	latdisplay
CxC	10_1	8	8	48	225	0	latdisplay
CxC	10_1	8	8	48	230	0	latdisplay
CxC	10_1	8	8	48	235	0	latdisplay
CxC	10_1	8	8	48	240	0	latdisplay
CxC	10_1	8	8	48	245	0	latdisplay
CxC	10_1	8	8	48	250	1	latdisplay
CxC	10_1	8	8	48	255	0	latdisplay
CxC	10_1	8	8	48	260	0	latdisplay
CxC	10_1	8	8	48	265	0	latdisplay
CxC	10_1	8	8	48	270	0	latdisplay
CxC	10_1	8	8	48	275	0	latdisplay
CxC	10_1	8	8	48	280	0	latdisplay
CxC	10_1	8	8	48	285	0	latdisplay
CxC	10_1	8	8	48	290	0	latdisplay
CxC	10_1	8	8	48	295	0	latdisplay
CxC	10_1	8	8	48	300	0	latdisplay
CxC	11_1	6	6	49	5	0	latdisplay
CxC	11_1	6	6	49	10	0	latdisplay
CxC	11_1	6	6	49	15	0	latdisplay
CxC	11_1	6	6	49	20	0	latdisplay
CxC	11_1	6	6	49	25	0	latdisplay
CxC	11_1	6	6	49	30	0	latdisplay
CxC	11_1	6	6	49	35	0	latdisplay
CxC	11_1	6	6	49	40	0	latdisplay
CxC	11_1	6	6	49	45	0	latdisplay
CxC	11_1	6	6	49	50	0	latdisplay
CxC	11_1	6	6	49	55	0	latdisplay
CxC	11_1	6	6	49	60	0	latdisplay
CxC	11_1	6	6	49	65	1	latdisplay
CxC	11_1	6	6	49	70	0	latdisplay
CxC	11_1	6	6	49	75	0	latdisplay
CxC	11_1	6	6	49	80	1	latdisplay
CxC	11_1	6	6	49	85	0	latdisplay
CxC	11_1	6	6	49	90	0	latdisplay
CxC	11_1	6	6	49	95	0	latdisplay
CxC	11_1	6	6	49	100	1	latdisplay
CxC	11_1	6	6	49	105	0	latdisplay
CxC	11_1	6	6	49	110	0	latdisplay
CxC	11_1	6	6	49	115	0	latdisplay

CxC	11_1	6	6	49	120	0	latdisplay
CxC	11_1	6	6	49	125	1	latdisplay
CxC	11_1	6	6	49	130	0	latdisplay
CxC	11_1	6	6	49	135	0	latdisplay
CxC	11_1	6	6	49	140	0	latdisplay
CxC	11_1	6	6	49	145	0	latdisplay
CxC	11_1	6	6	49	150	0	latdisplay
CxC	11_1	6	6	49	155	0	latdisplay
CxC	11_1	6	6	49	160	1	latdisplay
CxC	11_1	6	6	49	165	0	latdisplay
CxC	11_1	6	6	49	170	1	latdisplay
CxC	11_1	6	6	49	175	0	latdisplay
CxC	11_1	6	6	49	180	0	latdisplay
CxC	11_1	6	6	49	185	0	latdisplay
CxC	11_1	6	6	49	190	1	latdisplay
CxC	11_1	6	6	49	195	0	latdisplay
CxC	11_1	6	6	49	200	0	latdisplay
CxC	11_1	6	6	49	205	0	latdisplay
CxC	11_1	6	6	49	210	0	latdisplay
CxC	11_1	6	6	49	215	0	latdisplay
CxC	11_1	6	6	49	220	0	latdisplay
CxC	11_1	6	6	49	225	0	latdisplay
CxC	11_1	6	6	49	230	0	latdisplay
CxC	11_1	6	6	49	235	0	latdisplay
CxC	11_1	6	6	49	240	0	latdisplay
CxC	11_1	6	6	49	245	0	latdisplay
CxC	11_1	6	6	49	250	0	latdisplay
CxC	11_1	6	6	49	255	0	latdisplay
CxC	11_1	6	6	49	260	0	latdisplay
CxC	11_1	6	6	49	265	0	latdisplay
CxC	11_1	6	6	49	270	0	latdisplay
CxC	11_1	6	6	49	275	0	latdisplay
CxC	11_1	6	6	49	280	0	latdisplay
CxC	11_1	6	6	49	285	0	latdisplay
CxC	11_1	6	6	49	290	0	latdisplay
CxC	11_1	6	6	49	295	0	latdisplay
CxC	11_1	6	6	49	300	0	latdisplay
CxC	12_1	7	7	50	5	0	latdisplay
CxC	12_1	7	7	50	10	0	latdisplay
CxC	12_1	7	7	50	15	0	latdisplay
CxC	12_1	7	7	50	20	0	latdisplay
CxC	12_1	7	7	50	25	0	latdisplay
CxC	12_1	7	7	50	30	0	latdisplay
CxC	12_1	7	7	50	35	0	latdisplay
CxC	12_1	7	7	50	40	0	latdisplay
CxC	12_1	7	7	50	45	0	latdisplay
CxC	12_1	7	7	50	50	1	latdisplay
CxC	12_1	7	7	50	55	1	latdisplay

CxC	12_1	7	7	50	60	0	latdisplay
CxC	12_1	7	7	50	65	0	latdisplay
CxC	12_1	7	7	50	70	0	latdisplay
CxC	12_1	7	7	50	75	0	latdisplay
CxC	12_1	7	7	50	80	1	latdisplay
CxC	12_1	7	7	50	85	0	latdisplay
CxC	12_1	7	7	50	90	0	latdisplay
CxC	12_1	7	7	50	95	0	latdisplay
CxC	12_1	7	7	50	100	0	latdisplay
CxC	12_1	7	7	50	105	0	latdisplay
CxC	12_1	7	7	50	110	1	latdisplay
CxC	12_1	7	7	50	115	0	latdisplay
CxC	12_1	7	7	50	120	1	latdisplay
CxC	12_1	7	7	50	125	0	latdisplay
CxC	12_1	7	7	50	130	0	latdisplay
CxC	12_1	7	7	50	135	0	latdisplay
CxC	12_1	7	7	50	140	0	latdisplay
CxC	12_1	7	7	50	145	0	latdisplay
CxC	12_1	7	7	50	150	0	latdisplay
CxC	12_1	7	7	50	155	0	latdisplay
CxC	12_1	7	7	50	160	0	latdisplay
CxC	12_1	7	7	50	165	1	latdisplay
CxC	12_1	7	7	50	170	1	latdisplay
CxC	12_1	7	7	50	175	0	latdisplay
CxC	12_1	7	7	50	180	1	latdisplay
CxC	12_1	7	7	50	185	1	latdisplay
CxC	12_1	7	7	50	190	0	latdisplay
CxC	12_1	7	7	50	195	1	latdisplay
CxC	12_1	7	7	50	200	0	latdisplay
CxC	12_1	7	7	50	205	0	latdisplay
CxC	12_1	7	7	50	210	0	latdisplay
CxC	12_1	7	7	50	215	0	latdisplay
CxC	12_1	7	7	50	220	0	latdisplay
CxC	12_1	7	7	50	225	0	latdisplay
CxC	12_1	7	7	50	230	1	latdisplay
CxC	12_1	7	7	50	235	0	latdisplay
CxC	12_1	7	7	50	240	0	latdisplay
CxC	12_1	7	7	50	245	0	latdisplay
CxC	12_1	7	7	50	250	0	latdisplay
CxC	12_1	7	7	50	255	0	latdisplay
CxC	12_1	7	7	50	260	0	latdisplay
CxC	12_1	7	7	50	265	0	latdisplay
CxC	12_1	7	7	50	270	0	latdisplay
CxC	12_1	7	7	50	275	1	latdisplay
CxC	12_1	7	7	50	280	0	latdisplay
CxC	12_1	7	7	50	285	0	latdisplay
CxC	12_1	7	7	50	290	1	latdisplay
CxC	12_1	7	7	50	295	0	latdisplay

CxC	12_1	7	7	50	300	0	latdisplay
CxC	13_1	11	16	51	5	1	latdisplay
CxC	13_1	11	16	51	10	0	latdisplay
CxC	13_1	11	16	51	15	0	latdisplay
CxC	13_1	11	16	51	20	0	latdisplay
CxC	13_1	11	16	51	25	0	latdisplay
CxC	13_1	11	16	51	30	0	latdisplay
CxC	13_1	11	16	51	35	0	latdisplay
CxC	13_1	11	16	51	40	0	latdisplay
CxC	13_1	11	16	51	45	0	latdisplay
CxC	13_1	11	16	51	50	0	latdisplay
CxC	13_1	11	16	51	55	0	latdisplay
CxC	13_1	11	16	51	60	0	latdisplay
CxC	13_1	11	16	51	65	0	latdisplay
CxC	13_1	11	16	51	70	0	latdisplay
CxC	13_1	11	16	51	75	0	latdisplay
CxC	13_1	11	16	51	80	0	latdisplay
CxC	13_1	11	16	51	85	0	latdisplay
CxC	13_1	11	16	51	90	0	latdisplay
CxC	13_1	11	16	51	95	0	latdisplay
CxC	13_1	11	16	51	100	0	latdisplay
CxC	13_1	11	16	51	105	0	latdisplay
CxC	13_1	11	16	51	110	0	latdisplay
CxC	13_1	11	16	51	115	0	latdisplay
CxC	13_1	11	16	51	120	0	latdisplay
CxC	13_1	11	16	51	125	0	latdisplay
CxC	13_1	11	16	51	130	0	latdisplay
CxC	13_1	11	16	51	135	0	latdisplay
CxC	13_1	11	16	51	140	0	latdisplay
CxC	13_1	11	16	51	145	0	latdisplay
CxC	13_1	11	16	51	150	0	latdisplay
CxC	13_1	11	16	51	155	0	latdisplay
CxC	13_1	11	16	51	160	0	latdisplay
CxC	13_1	11	16	51	165	0	latdisplay
CxC	13_1	11	16	51	170	0	latdisplay
CxC	13_1	11	16	51	175	0	latdisplay
CxC	13_1	11	16	51	180	0	latdisplay
CxC	13_1	11	16	51	185	0	latdisplay
CxC	13_1	11	16	51	190	0	latdisplay
CxC	13_1	11	16	51	195	0	latdisplay
CxC	13_1	11	16	51	200	0	latdisplay
CxC	13_1	11	16	51	205	0	latdisplay
CxC	13_1	11	16	51	210	0	latdisplay
CxC	13_1	11	16	51	215	0	latdisplay
CxC	13_1	11	16	51	220	0	latdisplay
CxC	13_1	11	16	51	225	0	latdisplay
CxC	13_1	11	16	51	230	0	latdisplay
CxC	13_1	11	16	51	235	0	latdisplay

CxC	13_1	11	16	51	240	0	latdisplay
CxC	13_1	11	16	51	245	0	latdisplay
CxC	13_1	11	16	51	250	0	latdisplay
CxC	13_1	11	16	51	255	0	latdisplay
CxC	13_1	11	16	51	260	0	latdisplay
CxC	13_1	11	16	51	265	0	latdisplay
CxC	13_1	11	16	51	270	0	latdisplay
CxC	13_1	11	16	51	275	0	latdisplay
CxC	13_1	11	16	51	280	0	latdisplay
CxC	13_1	11	16	51	285	0	latdisplay
CxC	13_1	11	16	51	290	0	latdisplay
CxC	13_1	11	16	51	295	0	latdisplay
CxC	13_1	11	16	51	300	0	latdisplay
CxC	14_1	11	15	52	5	0	latdisplay
CxC	14_1	11	15	52	10	0	latdisplay
CxC	14_1	11	15	52	15	0	latdisplay
CxC	14_1	11	15	52	20	0	latdisplay
CxC	14_1	11	15	52	25	0	latdisplay
CxC	14_1	11	15	52	30	0	latdisplay
CxC	14_1	11	15	52	35	0	latdisplay
CxC	14_1	11	15	52	40	0	latdisplay
CxC	14_1	11	15	52	45	0	latdisplay
CxC	14_1	11	15	52	50	0	latdisplay
CxC	14_1	11	15	52	55	0	latdisplay
CxC	14_1	11	15	52	60	0	latdisplay
CxC	14_1	11	15	52	65	0	latdisplay
CxC	14_1	11	15	52	70	0	latdisplay
CxC	14_1	11	15	52	75	0	latdisplay
CxC	14_1	11	15	52	80	0	latdisplay
CxC	14_1	11	15	52	85	0	latdisplay
CxC	14_1	11	15	52	90	0	latdisplay
CxC	14_1	11	15	52	95	0	latdisplay
CxC	14_1	11	15	52	100	0	latdisplay
CxC	14_1	11	15	52	105	0	latdisplay
CxC	14_1	11	15	52	110	0	latdisplay
CxC	14_1	11	15	52	115	0	latdisplay
CxC	14_1	11	15	52	120	0	latdisplay
CxC	14_1	11	15	52	125	0	latdisplay
CxC	14_1	11	15	52	130	0	latdisplay
CxC	14_1	11	15	52	135	0	latdisplay
CxC	14_1	11	15	52	140	0	latdisplay
CxC	14_1	11	15	52	145	0	latdisplay
CxC	14_1	11	15	52	150	0	latdisplay
CxC	14_1	11	15	52	155	0	latdisplay
CxC	14_1	11	15	52	160	0	latdisplay
CxC	14_1	11	15	52	165	0	latdisplay
CxC	14_1	11	15	52	170	0	latdisplay
CxC	14_1	11	15	52	175	0	latdisplay

CxC	14_1	11	15	52	180	0	latdisplay
CxC	14_1	11	15	52	185	0	latdisplay
CxC	14_1	11	15	52	190	0	latdisplay
CxC	14_1	11	15	52	195	0	latdisplay
CxC	14_1	11	15	52	200	0	latdisplay
CxC	14_1	11	15	52	205	0	latdisplay
CxC	14_1	11	15	52	210	0	latdisplay
CxC	14_1	11	15	52	215	0	latdisplay
CxC	14_1	11	15	52	220	0	latdisplay
CxC	14_1	11	15	52	225	0	latdisplay
CxC	14_1	11	15	52	230	1	latdisplay
CxC	14_1	11	15	52	235	1	latdisplay
CxC	14_1	11	15	52	240	0	latdisplay
CxC	14_1	11	15	52	245	0	latdisplay
CxC	14_1	11	15	52	250	1	latdisplay
CxC	14_1	11	15	52	255	0	latdisplay
CxC	14_1	11	15	52	260	0	latdisplay
CxC	14_1	11	15	52	265	0	latdisplay
CxC	14_1	11	15	52	270	0	latdisplay
CxC	14_1	11	15	52	275	0	latdisplay
CxC	14_1	11	15	52	280	0	latdisplay
CxC	14_1	11	15	52	285	0	latdisplay
CxC	14_1	11	15	52	290	0	latdisplay
CxC	14_1	11	15	52	295	0	latdisplay
CxC	14_1	11	15	52	300	0	latdisplay
CxC	15_1	9	13	53	5	0	latdisplay
CxC	15_1	9	13	53	10	0	latdisplay
CxC	15_1	9	13	53	15	1	latdisplay
CxC	15_1	9	13	53	20	0	latdisplay
CxC	15_1	9	13	53	25	0	latdisplay
CxC	15_1	9	13	53	30	0	latdisplay
CxC	15_1	9	13	53	35	0	latdisplay
CxC	15_1	9	13	53	40	1	latdisplay
CxC	15_1	9	13	53	45	0	latdisplay
CxC	15_1	9	13	53	50	0	latdisplay
CxC	15_1	9	13	53	55	0	latdisplay
CxC	15_1	9	13	53	60	0	latdisplay
CxC	15_1	9	13	53	65	0	latdisplay
CxC	15_1	9	13	53	70	0	latdisplay
CxC	15_1	9	13	53	75	0	latdisplay
CxC	15_1	9	13	53	80	0	latdisplay
CxC	15_1	9	13	53	85	1	latdisplay
CxC	15_1	9	13	53	90	0	latdisplay
CxC	15_1	9	13	53	95	0	latdisplay
CxC	15_1	9	13	53	100	0	latdisplay
CxC	15_1	9	13	53	105	0	latdisplay
CxC	15_1	9	13	53	110	0	latdisplay
CxC	15_1	9	13	53	115	0	latdisplay

CxC	15_1	9	13	53	120	0	latdisplay
CxC	15_1	9	13	53	125	0	latdisplay
CxC	15_1	9	13	53	130	0	latdisplay
CxC	15_1	9	13	53	135	0	latdisplay
CxC	15_1	9	13	53	140	0	latdisplay
CxC	15_1	9	13	53	145	0	latdisplay
CxC	15_1	9	13	53	150	0	latdisplay
CxC	15_1	9	13	53	155	0	latdisplay
CxC	15_1	9	13	53	160	0	latdisplay
CxC	15_1	9	13	53	165	0	latdisplay
CxC	15_1	9	13	53	170	0	latdisplay
CxC	15_1	9	13	53	175	0	latdisplay
CxC	15_1	9	13	53	180	0	latdisplay
CxC	15_1	9	13	53	185	0	latdisplay
CxC	15_1	9	13	53	190	0	latdisplay
CxC	15_1	9	13	53	195	0	latdisplay
CxC	15_1	9	13	53	200	0	latdisplay
CxC	15_1	9	13	53	205	0	latdisplay
CxC	15_1	9	13	53	210	0	latdisplay
CxC	15_1	9	13	53	215	0	latdisplay
CxC	15_1	9	13	53	220	1	latdisplay
CxC	15_1	9	13	53	225	1	latdisplay
CxC	15_1	9	13	53	230	0	latdisplay
CxC	15_1	9	13	53	235	1	latdisplay
CxC	15_1	9	13	53	240	0	latdisplay
CxC	15_1	9	13	53	245	0	latdisplay
CxC	15_1	9	13	53	250	0	latdisplay
CxC	15_1	9	13	53	255	0	latdisplay
CxC	15_1	9	13	53	260	0	latdisplay
CxC	15_1	9	13	53	265	1	latdisplay
CxC	15_1	9	13	53	270	0	latdisplay
CxC	15_1	9	13	53	275	1	latdisplay
CxC	15_1	9	13	53	280	0	latdisplay
CxC	15_1	9	13	53	285	0	latdisplay
CxC	15_1	9	13	53	290	0	latdisplay
CxC	15_1	9	13	53	295	1	latdisplay
CxC	15_1	9	13	53	300	1	latdisplay
CxC	16_1	8	11	54	5	0	latdisplay
CxC	16_1	8	11	54	10	0	latdisplay
CxC	16_1	8	11	54	15	0	latdisplay
CxC	16_1	8	11	54	20	0	latdisplay
CxC	16_1	8	11	54	25	0	latdisplay
CxC	16_1	8	11	54	30	0	latdisplay
CxC	16_1	8	11	54	35	0	latdisplay
CxC	16_1	8	11	54	40	0	latdisplay
CxC	16_1	8	11	54	45	0	latdisplay
CxC	16_1	8	11	54	50	0	latdisplay
CxC	16_1	8	11	54	55	0	latdisplay

CxC	16_1	8	11	54	60	0	latdisplay
CxC	16_1	8	11	54	65	0	latdisplay
CxC	16_1	8	11	54	70	0	latdisplay
CxC	16_1	8	11	54	75	0	latdisplay
CxC	16_1	8	11	54	80	0	latdisplay
CxC	16_1	8	11	54	85	0	latdisplay
CxC	16_1	8	11	54	90	0	latdisplay
CxC	16_1	8	11	54	95	0	latdisplay
CxC	16_1	8	11	54	100	0	latdisplay
CxC	16_1	8	11	54	105	0	latdisplay
CxC	16_1	8	11	54	110	0	latdisplay
CxC	16_1	8	11	54	115	0	latdisplay
CxC	16_1	8	11	54	120	0	latdisplay
CxC	16_1	8	11	54	125	0	latdisplay
CxC	16_1	8	11	54	130	0	latdisplay
CxC	16_1	8	11	54	135	1	latdisplay
CxC	16_1	8	11	54	140	1	latdisplay
CxC	16_1	8	11	54	145	1	latdisplay
CxC	16_1	8	11	54	150	1	latdisplay
CxC	16_1	8	11	54	155	1	latdisplay
CxC	16_1	8	11	54	160	0	latdisplay
CxC	16_1	8	11	54	165	0	latdisplay
CxC	16_1	8	11	54	170	0	latdisplay
CxC	16_1	8	11	54	175	0	latdisplay
CxC	16_1	8	11	54	180	0	latdisplay
CxC	16_1	8	11	54	185	0	latdisplay
CxC	16_1	8	11	54	190	0	latdisplay
CxC	16_1	8	11	54	195	0	latdisplay
CxC	16_1	8	11	54	200	0	latdisplay
CxC	16_1	8	11	54	205	1	latdisplay
CxC	16_1	8	11	54	210	0	latdisplay
CxC	16_1	8	11	54	215	0	latdisplay
CxC	16_1	8	11	54	220	0	latdisplay
CxC	16_1	8	11	54	225	0	latdisplay
CxC	16_1	8	11	54	230	0	latdisplay
CxC	16_1	8	11	54	235	0	latdisplay
CxC	16_1	8	11	54	240	1	latdisplay
CxC	16_1	8	11	54	245	1	latdisplay
CxC	16_1	8	11	54	250	0	latdisplay
CxC	16_1	8	11	54	255	1	latdisplay
CxC	16_1	8	11	54	260	0	latdisplay
CxC	16_1	8	11	54	265	1	latdisplay
CxC	16_1	8	11	54	270	1	latdisplay
CxC	16_1	8	11	54	275	0	latdisplay
CxC	16_1	8	11	54	280	0	latdisplay
CxC	16_1	8	11	54	285	0	latdisplay
CxC	16_1	8	11	54	290	0	latdisplay
CxC	16_1	8	11	54	295	0	latdisplay

CxC	16_1	8	11	54	300	0	latdisplay
CxC	17_1	12	10	55	5	0	latdisplay
CxC	17_1	12	10	55	10	0	latdisplay
CxC	17_1	12	10	55	15	0	latdisplay
CxC	17_1	12	10	55	20	0	latdisplay
CxC	17_1	12	10	55	25	0	latdisplay
CxC	17_1	12	10	55	30	1	latdisplay
CxC	17_1	12	10	55	35	1	latdisplay
CxC	17_1	12	10	55	40	1	latdisplay
CxC	17_1	12	10	55	45	0	latdisplay
CxC	17_1	12	10	55	50	0	latdisplay
CxC	17_1	12	10	55	55	1	latdisplay
CxC	17_1	12	10	55	60	0	latdisplay
CxC	17_1	12	10	55	65	1	latdisplay
CxC	17_1	12	10	55	70	0	latdisplay
CxC	17_1	12	10	55	75	0	latdisplay
CxC	17_1	12	10	55	80	0	latdisplay
CxC	17_1	12	10	55	85	0	latdisplay
CxC	17_1	12	10	55	90	0	latdisplay
CxC	17_1	12	10	55	95	0	latdisplay
CxC	17_1	12	10	55	100	0	latdisplay
CxC	17_1	12	10	55	105	1	latdisplay
CxC	17_1	12	10	55	110	0	latdisplay
CxC	17_1	12	10	55	115	0	latdisplay
CxC	17_1	12	10	55	120	0	latdisplay
CxC	17_1	12	10	55	125	0	latdisplay
CxC	17_1	12	10	55	130	0	latdisplay
CxC	17_1	12	10	55	135	0	latdisplay
CxC	17_1	12	10	55	140	0	latdisplay
CxC	17_1	12	10	55	145	0	latdisplay
CxC	17_1	12	10	55	150	0	latdisplay
CxC	17_1	12	10	55	155	0	latdisplay
CxC	17_1	12	10	55	160	0	latdisplay
CxC	17_1	12	10	55	165	0	latdisplay
CxC	17_1	12	10	55	170	0	latdisplay
CxC	17_1	12	10	55	175	0	latdisplay
CxC	17_1	12	10	55	180	0	latdisplay
CxC	17_1	12	10	55	185	0	latdisplay
CxC	17_1	12	10	55	190	0	latdisplay
CxC	17_1	12	10	55	195	0	latdisplay
CxC	17_1	12	10	55	200	0	latdisplay
CxC	17_1	12	10	55	205	0	latdisplay
CxC	17_1	12	10	55	210	0	latdisplay
CxC	17_1	12	10	55	215	1	latdisplay
CxC	17_1	12	10	55	220	0	latdisplay
CxC	17_1	12	10	55	225	1	latdisplay
CxC	17_1	12	10	55	230	1	latdisplay
CxC	17_1	12	10	55	235	1	latdisplay

CxC	17_1	12	10	55	240	1	latdisplay
CxC	17_1	12	10	55	245	1	latdisplay
CxC	17_1	12	10	55	250	0	latdisplay
CxC	17_1	12	10	55	255	1	latdisplay
CxC	17_1	12	10	55	260	1	latdisplay
CxC	17_1	12	10	55	265	0	latdisplay
CxC	17_1	12	10	55	270	0	latdisplay
CxC	17_1	12	10	55	275	0	latdisplay
CxC	17_1	12	10	55	280	0	latdisplay
CxC	17_1	12	10	55	285	0	latdisplay
CxC	17_1	12	10	55	290	0	latdisplay
CxC	17_1	12	10	55	295	0	latdisplay
CxC	17_1	12	10	55	300	0	latdisplay
CxC	18_1	7	9	56	5	0	latdisplay
CxC	18_1	7	9	56	10	0	latdisplay
CxC	18_1	7	9	56	15	0	latdisplay
CxC	18_1	7	9	56	20	0	latdisplay
CxC	18_1	7	9	56	25	0	latdisplay
CxC	18_1	7	9	56	30	0	latdisplay
CxC	18_1	7	9	56	35	0	latdisplay
CxC	18_1	7	9	56	40	0	latdisplay
CxC	18_1	7	9	56	45	0	latdisplay
CxC	18_1	7	9	56	50	0	latdisplay
CxC	18_1	7	9	56	55	0	latdisplay
CxC	18_1	7	9	56	60	0	latdisplay
CxC	18_1	7	9	56	65	0	latdisplay
CxC	18_1	7	9	56	70	0	latdisplay
CxC	18_1	7	9	56	75	0	latdisplay
CxC	18_1	7	9	56	80	0	latdisplay
CxC	18_1	7	9	56	85	0	latdisplay
CxC	18_1	7	9	56	90	0	latdisplay
CxC	18_1	7	9	56	95	0	latdisplay
CxC	18_1	7	9	56	100	0	latdisplay
CxC	18_1	7	9	56	105	0	latdisplay
CxC	18_1	7	9	56	110	0	latdisplay
CxC	18_1	7	9	56	115	0	latdisplay
CxC	18_1	7	9	56	120	0	latdisplay
CxC	18_1	7	9	56	125	0	latdisplay
CxC	18_1	7	9	56	130	0	latdisplay
CxC	18_1	7	9	56	135	0	latdisplay
CxC	18_1	7	9	56	140	0	latdisplay
CxC	18_1	7	9	56	145	0	latdisplay
CxC	18_1	7	9	56	150	0	latdisplay
CxC	18_1	7	9	56	155	0	latdisplay
CxC	18_1	7	9	56	160	0	latdisplay
CxC	18_1	7	9	56	165	0	latdisplay
CxC	18_1	7	9	56	170	0	latdisplay
CxC	18_1	7	9	56	175	0	latdisplay

CxC	18_1	7	9	56	180	0	latdisplay
CxC	18_1	7	9	56	185	0	latdisplay
CxC	18_1	7	9	56	190	1	latdisplay
CxC	18_1	7	9	56	195	1	latdisplay
CxC	18_1	7	9	56	200	1	latdisplay
CxC	18_1	7	9	56	205	0	latdisplay
CxC	18_1	7	9	56	210	1	latdisplay
CxC	18_1	7	9	56	215	0	latdisplay
CxC	18_1	7	9	56	220	0	latdisplay
CxC	18_1	7	9	56	225	1	latdisplay
CxC	18_1	7	9	56	230	1	latdisplay
CxC	18_1	7	9	56	235	0	latdisplay
CxC	18_1	7	9	56	240	1	latdisplay
CxC	18_1	7	9	56	245	0	latdisplay
CxC	18_1	7	9	56	250	1	latdisplay
CxC	18_1	7	9	56	255	0	latdisplay
CxC	18_1	7	9	56	260	0	latdisplay
CxC	18_1	7	9	56	265	1	latdisplay
CxC	18_1	7	9	56	270	0	latdisplay
CxC	18_1	7	9	56	275	1	latdisplay
CxC	18_1	7	9	56	280	1	latdisplay
CxC	18_1	7	9	56	285	1	latdisplay
CxC	18_1	7	9	56	290	0	latdisplay
CxC	18_1	7	9	56	295	0	latdisplay
CxC	18_1	7	9	56	300	0	latdisplay
CxC	19_1	11	8	57	5	0	latdisplay
CxC	19_1	11	8	57	10	0	latdisplay
CxC	19_1	11	8	57	15	0	latdisplay
CxC	19_1	11	8	57	20	0	latdisplay
CxC	19_1	11	8	57	25	0	latdisplay
CxC	19_1	11	8	57	30	0	latdisplay
CxC	19_1	11	8	57	35	0	latdisplay
CxC	19_1	11	8	57	40	0	latdisplay
CxC	19_1	11	8	57	45	1	latdisplay
CxC	19_1	11	8	57	50	0	latdisplay
CxC	19_1	11	8	57	55	1	latdisplay
CxC	19_1	11	8	57	60	0	latdisplay
CxC	19_1	11	8	57	65	0	latdisplay
CxC	19_1	11	8	57	70	1	latdisplay
CxC	19_1	11	8	57	75	0	latdisplay
CxC	19_1	11	8	57	80	0	latdisplay
CxC	19_1	11	8	57	85	1	latdisplay
CxC	19_1	11	8	57	90	0	latdisplay
CxC	19_1	11	8	57	95	1	latdisplay
CxC	19_1	11	8	57	100	1	latdisplay
CxC	19_1	11	8	57	105	0	latdisplay
CxC	19_1	11	8	57	110	0	latdisplay
CxC	19_1	11	8	57	115	1	latdisplay

CxC	19_1	11	8	57	120	0	latdisplay
CxC	19_1	11	8	57	125	0	latdisplay
CxC	19_1	11	8	57	130	0	latdisplay
CxC	19_1	11	8	57	135	0	latdisplay
CxC	19_1	11	8	57	140	1	latdisplay
CxC	19_1	11	8	57	145	0	latdisplay
CxC	19_1	11	8	57	150	1	latdisplay
CxC	19_1	11	8	57	155	0	latdisplay
CxC	19_1	11	8	57	160	1	latdisplay
CxC	19_1	11	8	57	165	0	latdisplay
CxC	19_1	11	8	57	170	0	latdisplay
CxC	19_1	11	8	57	175	1	latdisplay
CxC	19_1	11	8	57	180	0	latdisplay
CxC	19_1	11	8	57	185	1	latdisplay
CxC	19_1	11	8	57	190	0	latdisplay
CxC	19_1	11	8	57	195	1	latdisplay
CxC	19_1	11	8	57	200	0	latdisplay
CxC	19_1	11	8	57	205	0	latdisplay
CxC	19_1	11	8	57	210	1	latdisplay
CxC	19_1	11	8	57	215	1	latdisplay
CxC	19_1	11	8	57	220	0	latdisplay
CxC	19_1	11	8	57	225	1	latdisplay
CxC	19_1	11	8	57	230	0	latdisplay
CxC	19_1	11	8	57	235	0	latdisplay
CxC	19_1	11	8	57	240	0	latdisplay
CxC	19_1	11	8	57	245	1	latdisplay
CxC	19_1	11	8	57	250	0	latdisplay
CxC	19_1	11	8	57	255	1	latdisplay
CxC	19_1	11	8	57	260	0	latdisplay
CxC	19_1	11	8	57	265	0	latdisplay
CxC	19_1	11	8	57	270	1	latdisplay
CxC	19_1	11	8	57	275	0	latdisplay
CxC	19_1	11	8	57	280	0	latdisplay
CxC	19_1	11	8	57	285	0	latdisplay
CxC	19_1	11	8	57	290	0	latdisplay
CxC	19_1	11	8	57	295	0	latdisplay
CxC	19_1	11	8	57	300	0	latdisplay
CxC	20_1	13	7	58	5	0	latdisplay
CxC	20_1	13	7	58	10	0	latdisplay
CxC	20_1	13	7	58	15	0	latdisplay
CxC	20_1	13	7	58	20	0	latdisplay
CxC	20_1	13	7	58	25	0	latdisplay
CxC	20_1	13	7	58	30	0	latdisplay
CxC	20_1	13	7	58	35	0	latdisplay
CxC	20_1	13	7	58	40	1	latdisplay
CxC	20_1	13	7	58	45	1	latdisplay
CxC	20_1	13	7	58	50	0	latdisplay
CxC	20_1	13	7	58	55	0	latdisplay

CxC	20_1	13	7	58	60	1	latdisplay
CxC	20_1	13	7	58	65	1	latdisplay
CxC	20_1	13	7	58	70	1	latdisplay
CxC	20_1	13	7	58	75	0	latdisplay
CxC	20_1	13	7	58	80	0	latdisplay
CxC	20_1	13	7	58	85	1	latdisplay
CxC	20_1	13	7	58	90	0	latdisplay
CxC	20_1	13	7	58	95	0	latdisplay
CxC	20_1	13	7	58	100	0	latdisplay
CxC	20_1	13	7	58	105	0	latdisplay
CxC	20_1	13	7	58	110	1	latdisplay
CxC	20_1	13	7	58	115	0	latdisplay
CxC	20_1	13	7	58	120	1	latdisplay
CxC	20_1	13	7	58	125	1	latdisplay
CxC	20_1	13	7	58	130	0	latdisplay
CxC	20_1	13	7	58	135	1	latdisplay
CxC	20_1	13	7	58	140	0	latdisplay
CxC	20_1	13	7	58	145	1	latdisplay
CxC	20_1	13	7	58	150	0	latdisplay
CxC	20_1	13	7	58	155	1	latdisplay
CxC	20_1	13	7	58	160	1	latdisplay
CxC	20_1	13	7	58	165	0	latdisplay
CxC	20_1	13	7	58	170	0	latdisplay
CxC	20_1	13	7	58	175	0	latdisplay
CxC	20_1	13	7	58	180	0	latdisplay
CxC	20_1	13	7	58	185	1	latdisplay
CxC	20_1	13	7	58	190	1	latdisplay
CxC	20_1	13	7	58	195	1	latdisplay
CxC	20_1	13	7	58	200	0	latdisplay
CxC	20_1	13	7	58	205	0	latdisplay
CxC	20_1	13	7	58	210	0	latdisplay
CxC	20_1	13	7	58	215	1	latdisplay
CxC	20_1	13	7	58	220	0	latdisplay
CxC	20_1	13	7	58	225	0	latdisplay
CxC	20_1	13	7	58	230	0	latdisplay
CxC	20_1	13	7	58	235	1	latdisplay
CxC	20_1	13	7	58	240	1	latdisplay
CxC	20_1	13	7	58	245	0	latdisplay
CxC	20_1	13	7	58	250	1	latdisplay
CxC	20_1	13	7	58	255	0	latdisplay
CxC	20_1	13	7	58	260	0	latdisplay
CxC	20_1	13	7	58	265	0	latdisplay
CxC	20_1	13	7	58	270	0	latdisplay
CxC	20_1	13	7	58	275	0	latdisplay
CxC	20_1	13	7	58	280	0	latdisplay
CxC	20_1	13	7	58	285	0	latdisplay
CxC	20_1	13	7	58	290	0	latdisplay
CxC	20_1	13	7	58	295	0	latdisplay

CxC	20_1	13	7	58	300	1	latdisplay
AxA	1_1	8	10	1	5	0	chasing
AxA	1_1	8	10	1	10	0	chasing
AxA	1_1	8	10	1	15	0	chasing
AxA	1_1	8	10	1	20	1	chasing
AxA	1_1	8	10	1	25	1	chasing
AxA	1_1	8	10	1	30	1	chasing
AxA	1_1	8	10	1	35	0	chasing
AxA	1_1	8	10	1	40	0	chasing
AxA	1_1	8	10	1	45	0	chasing
AxA	1_1	8	10	1	50	0	chasing
AxA	1_1	8	10	1	55	0	chasing
AxA	1_1	8	10	1	60	0	chasing
AxA	1_1	8	10	1	65	0	chasing
AxA	1_1	8	10	1	70	0	chasing
AxA	1_1	8	10	1	75	0	chasing
AxA	1_1	8	10	1	80	0	chasing
AxA	1_1	8	10	1	85	0	chasing
AxA	1_1	8	10	1	90	0	chasing
AxA	1_1	8	10	1	95	0	chasing
AxA	1_1	8	10	1	100	0	chasing
AxA	1_1	8	10	1	105	0	chasing
AxA	1_1	8	10	1	110	0	chasing
AxA	1_1	8	10	1	115	0	chasing
AxA	1_1	8	10	1	120	0	chasing
AxA	1_1	8	10	1	125	0	chasing
AxA	1_1	8	10	1	130	0	chasing
AxA	1_1	8	10	1	135	0	chasing
AxA	1_1	8	10	1	140	0	chasing
AxA	1_1	8	10	1	145	0	chasing
AxA	1_1	8	10	1	150	0	chasing
AxA	1_1	8	10	1	155	0	chasing
AxA	1_1	8	10	1	160	1	chasing
AxA	1_1	8	10	1	165	0	chasing
AxA	1_1	8	10	1	170	0	chasing
AxA	1_1	8	10	1	175	0	chasing
AxA	1_1	8	10	1	180	1	chasing
AxA	1_1	8	10	1	185	0	chasing
AxA	1_1	8	10	1	190	0	chasing
AxA	1_1	8	10	1	195	0	chasing
AxA	1_1	8	10	1	200	0	chasing
AxA	1_1	8	10	1	205	0	chasing
AxA	1_1	8	10	1	210	0	chasing
AxA	1_1	8	10	1	215	0	chasing
AxA	1_1	8	10	1	220	0	chasing
AxA	1_1	8	10	1	225	0	chasing
AxA	1_1	8	10	1	230	0	chasing
AxA	1_1	8	10	1	235	0	chasing

AxA	1_1	8	10	1	240	0	chasing
AxA	1_1	8	10	1	245	0	chasing
AxA	1_1	8	10	1	250	0	chasing
AxA	1_1	8	10	1	255	0	chasing
AxA	1_1	8	10	1	260	0	chasing
AxA	1_1	8	10	1	265	0	chasing
AxA	1_1	8	10	1	270	0	chasing
AxA	1_1	8	10	1	275	0	chasing
AxA	1_1	8	10	1	280	0	chasing
AxA	1_1	8	10	1	285	0	chasing
AxA	1_1	8	10	1	290	0	chasing
AxA	1_1	8	10	1	295	1	chasing
AxA	1_1	8	10	1	300	0	chasing
AxA	2_1	9	7	2	5	1	chasing
AxA	2_1	9	7	2	10	0	chasing
AxA	2_1	9	7	2	15	0	chasing
AxA	2_1	9	7	2	20	0	chasing
AxA	2_1	9	7	2	25	0	chasing
AxA	2_1	9	7	2	30	0	chasing
AxA	2_1	9	7	2	35	0	chasing
AxA	2_1	9	7	2	40	1	chasing
AxA	2_1	9	7	2	45	0	chasing
AxA	2_1	9	7	2	50	1	chasing
AxA	2_1	9	7	2	55	0	chasing
AxA	2_1	9	7	2	60	0	chasing
AxA	2_1	9	7	2	65	0	chasing
AxA	2_1	9	7	2	70	0	chasing
AxA	2_1	9	7	2	75	0	chasing
AxA	2_1	9	7	2	80	0	chasing
AxA	2_1	9	7	2	85	0	chasing
AxA	2_1	9	7	2	90	0	chasing
AxA	2_1	9	7	2	95	0	chasing
AxA	2_1	9	7	2	100	0	chasing
AxA	2_1	9	7	2	105	0	chasing
AxA	2_1	9	7	2	110	0	chasing
AxA	2_1	9	7	2	115	0	chasing
AxA	2_1	9	7	2	120	0	chasing
AxA	2_1	9	7	2	125	0	chasing
AxA	2_1	9	7	2	130	0	chasing
AxA	2_1	9	7	2	135	0	chasing
AxA	2_1	9	7	2	140	0	chasing
AxA	2_1	9	7	2	145	0	chasing
AxA	2_1	9	7	2	150	0	chasing
AxA	2_1	9	7	2	155	0	chasing
AxA	2_1	9	7	2	160	0	chasing
AxA	2_1	9	7	2	165	0	chasing
AxA	2_1	9	7	2	170	0	chasing
AxA	2_1	9	7	2	175	0	chasing

AxA	2_1	9	7	2	180	0	chasing
AxA	2_1	9	7	2	185	1	chasing
AxA	2_1	9	7	2	190	1	chasing
AxA	2_1	9	7	2	195	0	chasing
AxA	2_1	9	7	2	200	0	chasing
AxA	2_1	9	7	2	205	0	chasing
AxA	2_1	9	7	2	210	0	chasing
AxA	2_1	9	7	2	215	1	chasing
AxA	2_1	9	7	2	220	0	chasing
AxA	2_1	9	7	2	225	0	chasing
AxA	2_1	9	7	2	230	0	chasing
AxA	2_1	9	7	2	235	0	chasing
AxA	2_1	9	7	2	240	0	chasing
AxA	2_1	9	7	2	245	0	chasing
AxA	2_1	9	7	2	250	0	chasing
AxA	2_1	9	7	2	255	0	chasing
AxA	2_1	9	7	2	260	0	chasing
AxA	2_1	9	7	2	265	0	chasing
AxA	2_1	9	7	2	270	0	chasing
AxA	2_1	9	7	2	275	0	chasing
AxA	2_1	9	7	2	280	0	chasing
AxA	2_1	9	7	2	285	0	chasing
AxA	2_1	9	7	2	290	0	chasing
AxA	2_1	9	7	2	295	0	chasing
AxA	2_1	9	7	2	300	0	chasing
AxA	3_1	8	13	3	5	0	chasing
AxA	3_1	8	13	3	10	0	chasing
AxA	3_1	8	13	3	15	0	chasing
AxA	3_1	8	13	3	20	0	chasing
AxA	3_1	8	13	3	25	0	chasing
AxA	3_1	8	13	3	30	0	chasing
AxA	3_1	8	13	3	35	0	chasing
AxA	3_1	8	13	3	40	0	chasing
AxA	3_1	8	13	3	45	0	chasing
AxA	3_1	8	13	3	50	0	chasing
AxA	3_1	8	13	3	55	1	chasing
AxA	3_1	8	13	3	60	0	chasing
AxA	3_1	8	13	3	65	0	chasing
AxA	3_1	8	13	3	70	0	chasing
AxA	3_1	8	13	3	75	0	chasing
AxA	3_1	8	13	3	80	0	chasing
AxA	3_1	8	13	3	85	0	chasing
AxA	3_1	8	13	3	90	0	chasing
AxA	3_1	8	13	3	95	0	chasing
AxA	3_1	8	13	3	100	0	chasing
AxA	3_1	8	13	3	105	0	chasing
AxA	3_1	8	13	3	110	0	chasing
AxA	3_1	8	13	3	115	0	chasing

AxA	3_1	8	13	3	120	0	chasing
AxA	3_1	8	13	3	125	0	chasing
AxA	3_1	8	13	3	130	0	chasing
AxA	3_1	8	13	3	135	0	chasing
AxA	3_1	8	13	3	140	0	chasing
AxA	3_1	8	13	3	145	0	chasing
AxA	3_1	8	13	3	150	1	chasing
AxA	3_1	8	13	3	155	0	chasing
AxA	3_1	8	13	3	160	0	chasing
AxA	3_1	8	13	3	165	0	chasing
AxA	3_1	8	13	3	170	0	chasing
AxA	3_1	8	13	3	175	1	chasing
AxA	3_1	8	13	3	180	0	chasing
AxA	3_1	8	13	3	185	0	chasing
AxA	3_1	8	13	3	190	0	chasing
AxA	3_1	8	13	3	195	0	chasing
AxA	3_1	8	13	3	200	0	chasing
AxA	3_1	8	13	3	205	0	chasing
AxA	3_1	8	13	3	210	0	chasing
AxA	3_1	8	13	3	215	0	chasing
AxA	3_1	8	13	3	220	0	chasing
AxA	3_1	8	13	3	225	0	chasing
AxA	3_1	8	13	3	230	0	chasing
AxA	3_1	8	13	3	235	0	chasing
AxA	3_1	8	13	3	240	0	chasing
AxA	3_1	8	13	3	245	0	chasing
AxA	3_1	8	13	3	250	0	chasing
AxA	3_1	8	13	3	255	0	chasing
AxA	3_1	8	13	3	260	0	chasing
AxA	3_1	8	13	3	265	0	chasing
AxA	3_1	8	13	3	270	0	chasing
AxA	3_1	8	13	3	275	0	chasing
AxA	3_1	8	13	3	280	0	chasing
AxA	3_1	8	13	3	285	0	chasing
AxA	3_1	8	13	3	290	0	chasing
AxA	3_1	8	13	3	295	0	chasing
AxA	3_1	8	13	3	300	0	chasing
AxA	4_1	11	10	4	5	0	chasing
AxA	4_1	11	10	4	10	0	chasing
AxA	4_1	11	10	4	15	0	chasing
AxA	4_1	11	10	4	20	0	chasing
AxA	4_1	11	10	4	25	0	chasing
AxA	4_1	11	10	4	30	0	chasing
AxA	4_1	11	10	4	35	0	chasing
AxA	4_1	11	10	4	40	0	chasing
AxA	4_1	11	10	4	45	0	chasing
AxA	4_1	11	10	4	50	0	chasing
AxA	4_1	11	10	4	55	0	chasing

AxA	4_1	11	10	4	60	0	chasing
AxA	4_1	11	10	4	65	0	chasing
AxA	4_1	11	10	4	70	0	chasing
AxA	4_1	11	10	4	75	0	chasing
AxA	4_1	11	10	4	80	0	chasing
AxA	4_1	11	10	4	85	0	chasing
AxA	4_1	11	10	4	90	0	chasing
AxA	4_1	11	10	4	95	0	chasing
AxA	4_1	11	10	4	100	0	chasing
AxA	4_1	11	10	4	105	0	chasing
AxA	4_1	11	10	4	110	0	chasing
AxA	4_1	11	10	4	115	0	chasing
AxA	4_1	11	10	4	120	0	chasing
AxA	4_1	11	10	4	125	0	chasing
AxA	4_1	11	10	4	130	0	chasing
AxA	4_1	11	10	4	135	0	chasing
AxA	4_1	11	10	4	140	1	chasing
AxA	4_1	11	10	4	145	0	chasing
AxA	4_1	11	10	4	150	0	chasing
AxA	4_1	11	10	4	155	0	chasing
AxA	4_1	11	10	4	160	0	chasing
AxA	4_1	11	10	4	165	0	chasing
AxA	4_1	11	10	4	170	0	chasing
AxA	4_1	11	10	4	175	0	chasing
AxA	4_1	11	10	4	180	0	chasing
AxA	4_1	11	10	4	185	0	chasing
AxA	4_1	11	10	4	190	0	chasing
AxA	4_1	11	10	4	195	0	chasing
AxA	4_1	11	10	4	200	0	chasing
AxA	4_1	11	10	4	205	0	chasing
AxA	4_1	11	10	4	210	0	chasing
AxA	4_1	11	10	4	215	0	chasing
AxA	4_1	11	10	4	220	0	chasing
AxA	4_1	11	10	4	225	0	chasing
AxA	4_1	11	10	4	230	0	chasing
AxA	4_1	11	10	4	235	0	chasing
AxA	4_1	11	10	4	240	0	chasing
AxA	4_1	11	10	4	245	0	chasing
AxA	4_1	11	10	4	250	0	chasing
AxA	4_1	11	10	4	255	0	chasing
AxA	4_1	11	10	4	260	0	chasing
AxA	4_1	11	10	4	265	0	chasing
AxA	4_1	11	10	4	270	0	chasing
AxA	4_1	11	10	4	275	0	chasing
AxA	4_1	11	10	4	280	0	chasing
AxA	4_1	11	10	4	285	0	chasing
AxA	4_1	11	10	4	290	0	chasing
AxA	4_1	11	10	4	295	0	chasing

AxA	4_1	11	10	4	300	0	chasing
AxA	5_1	11	12	5	5	0	chasing
AxA	5_1	11	12	5	10	0	chasing
AxA	5_1	11	12	5	15	0	chasing
AxA	5_1	11	12	5	20	1	chasing
AxA	5_1	11	12	5	25	0	chasing
AxA	5_1	11	12	5	30	0	chasing
AxA	5_1	11	12	5	35	0	chasing
AxA	5_1	11	12	5	40	0	chasing
AxA	5_1	11	12	5	45	0	chasing
AxA	5_1	11	12	5	50	0	chasing
AxA	5_1	11	12	5	55	0	chasing
AxA	5_1	11	12	5	60	0	chasing
AxA	5_1	11	12	5	65	0	chasing
AxA	5_1	11	12	5	70	0	chasing
AxA	5_1	11	12	5	75	0	chasing
AxA	5_1	11	12	5	80	0	chasing
AxA	5_1	11	12	5	85	0	chasing
AxA	5_1	11	12	5	90	0	chasing
AxA	5_1	11	12	5	95	0	chasing
AxA	5_1	11	12	5	100	0	chasing
AxA	5_1	11	12	5	105	0	chasing
AxA	5_1	11	12	5	110	0	chasing
AxA	5_1	11	12	5	115	0	chasing
AxA	5_1	11	12	5	120	0	chasing
AxA	5_1	11	12	5	125	0	chasing
AxA	5_1	11	12	5	130	0	chasing
AxA	5_1	11	12	5	135	0	chasing
AxA	5_1	11	12	5	140	0	chasing
AxA	5_1	11	12	5	145	0	chasing
AxA	5_1	11	12	5	150	1	chasing
AxA	5_1	11	12	5	155	0	chasing
AxA	5_1	11	12	5	160	0	chasing
AxA	5_1	11	12	5	165	0	chasing
AxA	5_1	11	12	5	170	0	chasing
AxA	5_1	11	12	5	175	0	chasing
AxA	5_1	11	12	5	180	0	chasing
AxA	5_1	11	12	5	185	0	chasing
AxA	5_1	11	12	5	190	0	chasing
AxA	5_1	11	12	5	195	0	chasing
AxA	5_1	11	12	5	200	0	chasing
AxA	5_1	11	12	5	205	0	chasing
AxA	5_1	11	12	5	210	0	chasing
AxA	5_1	11	12	5	215	1	chasing
AxA	5_1	11	12	5	220	0	chasing
AxA	5_1	11	12	5	225	0	chasing
AxA	5_1	11	12	5	230	0	chasing
AxA	5_1	11	12	5	235	0	chasing

AxA	5_1	11	12	5	240	0	chasing
AxA	5_1	11	12	5	245	0	chasing
AxA	5_1	11	12	5	250	0	chasing
AxA	5_1	11	12	5	255	0	chasing
AxA	5_1	11	12	5	260	0	chasing
AxA	5_1	11	12	5	265	0	chasing
AxA	5_1	11	12	5	270	0	chasing
AxA	5_1	11	12	5	275	0	chasing
AxA	5_1	11	12	5	280	0	chasing
AxA	5_1	11	12	5	285	0	chasing
AxA	5_1	11	12	5	290	0	chasing
AxA	5_1	11	12	5	295	0	chasing
AxA	5_1	11	12	5	300	0	chasing
AxA	6_1	13	11	6	5	0	chasing
AxA	6_1	13	11	6	10	0	chasing
AxA	6_1	13	11	6	15	0	chasing
AxA	6_1	13	11	6	20	0	chasing
AxA	6_1	13	11	6	25	0	chasing
AxA	6_1	13	11	6	30	0	chasing
AxA	6_1	13	11	6	35	0	chasing
AxA	6_1	13	11	6	40	0	chasing
AxA	6_1	13	11	6	45	1	chasing
AxA	6_1	13	11	6	50	0	chasing
AxA	6_1	13	11	6	55	0	chasing
AxA	6_1	13	11	6	60	0	chasing
AxA	6_1	13	11	6	65	0	chasing
AxA	6_1	13	11	6	70	0	chasing
AxA	6_1	13	11	6	75	0	chasing
AxA	6_1	13	11	6	80	0	chasing
AxA	6_1	13	11	6	85	0	chasing
AxA	6_1	13	11	6	90	0	chasing
AxA	6_1	13	11	6	95	0	chasing
AxA	6_1	13	11	6	100	0	chasing
AxA	6_1	13	11	6	105	0	chasing
AxA	6_1	13	11	6	110	0	chasing
AxA	6_1	13	11	6	115	0	chasing
AxA	6_1	13	11	6	120	0	chasing
AxA	6_1	13	11	6	125	0	chasing
AxA	6_1	13	11	6	130	0	chasing
AxA	6_1	13	11	6	135	0	chasing
AxA	6_1	13	11	6	140	0	chasing
AxA	6_1	13	11	6	145	0	chasing
AxA	6_1	13	11	6	150	0	chasing
AxA	6_1	13	11	6	155	0	chasing
AxA	6_1	13	11	6	160	0	chasing
AxA	6_1	13	11	6	165	0	chasing
AxA	6_1	13	11	6	170	0	chasing
AxA	6_1	13	11	6	175	0	chasing

AxA	6_1	13	11	6	180	0	chasing
AxA	6_1	13	11	6	185	0	chasing
AxA	6_1	13	11	6	190	0	chasing
AxA	6_1	13	11	6	195	0	chasing
AxA	6_1	13	11	6	200	0	chasing
AxA	6_1	13	11	6	205	0	chasing
AxA	6_1	13	11	6	210	0	chasing
AxA	6_1	13	11	6	215	0	chasing
AxA	6_1	13	11	6	220	0	chasing
AxA	6_1	13	11	6	225	0	chasing
AxA	6_1	13	11	6	230	1	chasing
AxA	6_1	13	11	6	235	0	chasing
AxA	6_1	13	11	6	240	0	chasing
AxA	6_1	13	11	6	245	0	chasing
AxA	6_1	13	11	6	250	0	chasing
AxA	6_1	13	11	6	255	0	chasing
AxA	6_1	13	11	6	260	0	chasing
AxA	6_1	13	11	6	265	0	chasing
AxA	6_1	13	11	6	270	0	chasing
AxA	6_1	13	11	6	275	0	chasing
AxA	6_1	13	11	6	280	0	chasing
AxA	6_1	13	11	6	285	0	chasing
AxA	6_1	13	11	6	290	0	chasing
AxA	6_1	13	11	6	295	0	chasing
AxA	6_1	13	11	6	300	0	chasing
AxA	7_1	10	12	7	5	0	chasing
AxA	7_1	10	12	7	10	0	chasing
AxA	7_1	10	12	7	15	0	chasing
AxA	7_1	10	12	7	20	0	chasing
AxA	7_1	10	12	7	25	0	chasing
AxA	7_1	10	12	7	30	0	chasing
AxA	7_1	10	12	7	35	0	chasing
AxA	7_1	10	12	7	40	1	chasing
AxA	7_1	10	12	7	45	0	chasing
AxA	7_1	10	12	7	50	0	chasing
AxA	7_1	10	12	7	55	0	chasing
AxA	7_1	10	12	7	60	0	chasing
AxA	7_1	10	12	7	65	0	chasing
AxA	7_1	10	12	7	70	0	chasing
AxA	7_1	10	12	7	75	0	chasing
AxA	7_1	10	12	7	80	0	chasing
AxA	7_1	10	12	7	85	0	chasing
AxA	7_1	10	12	7	90	0	chasing
AxA	7_1	10	12	7	95	0	chasing
AxA	7_1	10	12	7	100	0	chasing
AxA	7_1	10	12	7	105	0	chasing
AxA	7_1	10	12	7	110	0	chasing
AxA	7_1	10	12	7	115	0	chasing

AxA	7_1	10	12	7	120	0	chasing
AxA	7_1	10	12	7	125	0	chasing
AxA	7_1	10	12	7	130	0	chasing
AxA	7_1	10	12	7	135	0	chasing
AxA	7_1	10	12	7	140	0	chasing
AxA	7_1	10	12	7	145	0	chasing
AxA	7_1	10	12	7	150	0	chasing
AxA	7_1	10	12	7	155	0	chasing
AxA	7_1	10	12	7	160	0	chasing
AxA	7_1	10	12	7	165	0	chasing
AxA	7_1	10	12	7	170	0	chasing
AxA	7_1	10	12	7	175	0	chasing
AxA	7_1	10	12	7	180	0	chasing
AxA	7_1	10	12	7	185	0	chasing
AxA	7_1	10	12	7	190	0	chasing
AxA	7_1	10	12	7	195	0	chasing
AxA	7_1	10	12	7	200	0	chasing
AxA	7_1	10	12	7	205	0	chasing
AxA	7_1	10	12	7	210	0	chasing
AxA	7_1	10	12	7	215	0	chasing
AxA	7_1	10	12	7	220	0	chasing
AxA	7_1	10	12	7	225	0	chasing
AxA	7_1	10	12	7	230	0	chasing
AxA	7_1	10	12	7	235	0	chasing
AxA	7_1	10	12	7	240	0	chasing
AxA	7_1	10	12	7	245	0	chasing
AxA	7_1	10	12	7	250	0	chasing
AxA	7_1	10	12	7	255	0	chasing
AxA	7_1	10	12	7	260	0	chasing
AxA	7_1	10	12	7	265	0	chasing
AxA	7_1	10	12	7	270	0	chasing
AxA	7_1	10	12	7	275	0	chasing
AxA	7_1	10	12	7	280	1	chasing
AxA	7_1	10	12	7	285	0	chasing
AxA	7_1	10	12	7	290	0	chasing
AxA	7_1	10	12	7	295	0	chasing
AxA	7_1	10	12	7	300	0	chasing
AxA	8_1	12	11	8	5	0	chasing
AxA	8_1	12	11	8	10	0	chasing
AxA	8_1	12	11	8	15	0	chasing
AxA	8_1	12	11	8	20	0	chasing
AxA	8_1	12	11	8	25	0	chasing
AxA	8_1	12	11	8	30	0	chasing
AxA	8_1	12	11	8	35	0	chasing
AxA	8_1	12	11	8	40	0	chasing
AxA	8_1	12	11	8	45	0	chasing
AxA	8_1	12	11	8	50	0	chasing
AxA	8_1	12	11	8	55	0	chasing

AxA	8_1	12	11	8	60	0	chasing
AxA	8_1	12	11	8	65	0	chasing
AxA	8_1	12	11	8	70	0	chasing
AxA	8_1	12	11	8	75	0	chasing
AxA	8_1	12	11	8	80	0	chasing
AxA	8_1	12	11	8	85	0	chasing
AxA	8_1	12	11	8	90	0	chasing
AxA	8_1	12	11	8	95	0	chasing
AxA	8_1	12	11	8	100	0	chasing
AxA	8_1	12	11	8	105	0	chasing
AxA	8_1	12	11	8	110	0	chasing
AxA	8_1	12	11	8	115	0	chasing
AxA	8_1	12	11	8	120	0	chasing
AxA	8_1	12	11	8	125	0	chasing
AxA	8_1	12	11	8	130	0	chasing
AxA	8_1	12	11	8	135	0	chasing
AxA	8_1	12	11	8	140	0	chasing
AxA	8_1	12	11	8	145	0	chasing
AxA	8_1	12	11	8	150	0	chasing
AxA	8_1	12	11	8	155	0	chasing
AxA	8_1	12	11	8	160	0	chasing
AxA	8_1	12	11	8	165	0	chasing
AxA	8_1	12	11	8	170	0	chasing
AxA	8_1	12	11	8	175	0	chasing
AxA	8_1	12	11	8	180	0	chasing
AxA	8_1	12	11	8	185	0	chasing
AxA	8_1	12	11	8	190	1	chasing
AxA	8_1	12	11	8	195	0	chasing
AxA	8_1	12	11	8	200	0	chasing
AxA	8_1	12	11	8	205	0	chasing
AxA	8_1	12	11	8	210	0	chasing
AxA	8_1	12	11	8	215	0	chasing
AxA	8_1	12	11	8	220	0	chasing
AxA	8_1	12	11	8	225	0	chasing
AxA	8_1	12	11	8	230	0	chasing
AxA	8_1	12	11	8	235	0	chasing
AxA	8_1	12	11	8	240	0	chasing
AxA	8_1	12	11	8	245	0	chasing
AxA	8_1	12	11	8	250	0	chasing
AxA	8_1	12	11	8	255	0	chasing
AxA	8_1	12	11	8	260	0	chasing
AxA	8_1	12	11	8	265	0	chasing
AxA	8_1	12	11	8	270	0	chasing
AxA	8_1	12	11	8	275	0	chasing
AxA	8_1	12	11	8	280	0	chasing
AxA	8_1	12	11	8	285	0	chasing
AxA	8_1	12	11	8	290	0	chasing
AxA	8_1	12	11	8	295	0	chasing

AxA	8_1	12	11	8	300	0	chasing
AxA	9_1	9	11	9	5	0	chasing
AxA	9_1	9	11	9	10	0	chasing
AxA	9_1	9	11	9	15	0	chasing
AxA	9_1	9	11	9	20	0	chasing
AxA	9_1	9	11	9	25	0	chasing
AxA	9_1	9	11	9	30	0	chasing
AxA	9_1	9	11	9	35	0	chasing
AxA	9_1	9	11	9	40	0	chasing
AxA	9_1	9	11	9	45	1	chasing
AxA	9_1	9	11	9	50	0	chasing
AxA	9_1	9	11	9	55	0	chasing
AxA	9_1	9	11	9	60	0	chasing
AxA	9_1	9	11	9	65	0	chasing
AxA	9_1	9	11	9	70	0	chasing
AxA	9_1	9	11	9	75	0	chasing
AxA	9_1	9	11	9	80	0	chasing
AxA	9_1	9	11	9	85	0	chasing
AxA	9_1	9	11	9	90	1	chasing
AxA	9_1	9	11	9	95	0	chasing
AxA	9_1	9	11	9	100	0	chasing
AxA	9_1	9	11	9	105	0	chasing
AxA	9_1	9	11	9	110	0	chasing
AxA	9_1	9	11	9	115	0	chasing
AxA	9_1	9	11	9	120	0	chasing
AxA	9_1	9	11	9	125	0	chasing
AxA	9_1	9	11	9	130	0	chasing
AxA	9_1	9	11	9	135	0	chasing
AxA	9_1	9	11	9	140	0	chasing
AxA	9_1	9	11	9	145	0	chasing
AxA	9_1	9	11	9	150	0	chasing
AxA	9_1	9	11	9	155	0	chasing
AxA	9_1	9	11	9	160	0	chasing
AxA	9_1	9	11	9	165	0	chasing
AxA	9_1	9	11	9	170	1	chasing
AxA	9_1	9	11	9	175	0	chasing
AxA	9_1	9	11	9	180	0	chasing
AxA	9_1	9	11	9	185	0	chasing
AxA	9_1	9	11	9	190	0	chasing
AxA	9_1	9	11	9	195	0	chasing
AxA	9_1	9	11	9	200	0	chasing
AxA	9_1	9	11	9	205	0	chasing
AxA	9_1	9	11	9	210	0	chasing
AxA	9_1	9	11	9	215	0	chasing
AxA	9_1	9	11	9	220	0	chasing
AxA	9_1	9	11	9	225	0	chasing
AxA	9_1	9	11	9	230	0	chasing
AxA	9_1	9	11	9	235	0	chasing

AxA	9_1	9	11	9	240	0	chasing
AxA	9_1	9	11	9	245	0	chasing
AxA	9_1	9	11	9	250	0	chasing
AxA	9_1	9	11	9	255	0	chasing
AxA	9_1	9	11	9	260	0	chasing
AxA	9_1	9	11	9	265	0	chasing
AxA	9_1	9	11	9	270	0	chasing
AxA	9_1	9	11	9	275	0	chasing
AxA	9_1	9	11	9	280	0	chasing
AxA	9_1	9	11	9	285	0	chasing
AxA	9_1	9	11	9	290	0	chasing
AxA	9_1	9	11	9	295	0	chasing
AxA	9_1	9	11	9	300	0	chasing
AxA	10_1	13	12	10	5	0	chasing
AxA	10_1	13	12	10	10	0	chasing
AxA	10_1	13	12	10	15	0	chasing
AxA	10_1	13	12	10	20	0	chasing
AxA	10_1	13	12	10	25	0	chasing
AxA	10_1	13	12	10	30	0	chasing
AxA	10_1	13	12	10	35	0	chasing
AxA	10_1	13	12	10	40	0	chasing
AxA	10_1	13	12	10	45	0	chasing
AxA	10_1	13	12	10	50	0	chasing
AxA	10_1	13	12	10	55	0	chasing
AxA	10_1	13	12	10	60	0	chasing
AxA	10_1	13	12	10	65	0	chasing
AxA	10_1	13	12	10	70	0	chasing
AxA	10_1	13	12	10	75	0	chasing
AxA	10_1	13	12	10	80	0	chasing
AxA	10_1	13	12	10	85	0	chasing
AxA	10_1	13	12	10	90	0	chasing
AxA	10_1	13	12	10	95	0	chasing
AxA	10_1	13	12	10	100	0	chasing
AxA	10_1	13	12	10	105	0	chasing
AxA	10_1	13	12	10	110	0	chasing
AxA	10_1	13	12	10	115	0	chasing
AxA	10_1	13	12	10	120	0	chasing
AxA	10_1	13	12	10	125	0	chasing
AxA	10_1	13	12	10	130	0	chasing
AxA	10_1	13	12	10	135	0	chasing
AxA	10_1	13	12	10	140	0	chasing
AxA	10_1	13	12	10	145	0	chasing
AxA	10_1	13	12	10	150	0	chasing
AxA	10_1	13	12	10	155	0	chasing
AxA	10_1	13	12	10	160	0	chasing
AxA	10_1	13	12	10	165	0	chasing
AxA	10_1	13	12	10	170	0	chasing
AxA	10_1	13	12	10	175	0	chasing

AxA	10_1	13	12	10	180	0	chasing
AxA	10_1	13	12	10	185	0	chasing
AxA	10_1	13	12	10	190	0	chasing
AxA	10_1	13	12	10	195	0	chasing
AxA	10_1	13	12	10	200	0	chasing
AxA	10_1	13	12	10	205	0	chasing
AxA	10_1	13	12	10	210	0	chasing
AxA	10_1	13	12	10	215	0	chasing
AxA	10_1	13	12	10	220	0	chasing
AxA	10_1	13	12	10	225	0	chasing
AxA	10_1	13	12	10	230	0	chasing
AxA	10_1	13	12	10	235	0	chasing
AxA	10_1	13	12	10	240	0	chasing
AxA	10_1	13	12	10	245	0	chasing
AxA	10_1	13	12	10	250	0	chasing
AxA	10_1	13	12	10	255	0	chasing
AxA	10_1	13	12	10	260	0	chasing
AxA	10_1	13	12	10	265	0	chasing
AxA	10_1	13	12	10	270	0	chasing
AxA	10_1	13	12	10	275	0	chasing
AxA	10_1	13	12	10	280	0	chasing
AxA	10_1	13	12	10	285	0	chasing
AxA	10_1	13	12	10	290	0	chasing
AxA	10_1	13	12	10	295	0	chasing
AxA	10_1	13	12	10	300	0	chasing
AxA	11_1	9	10	11	5	0	chasing
AxA	11_1	9	10	11	10	0	chasing
AxA	11_1	9	10	11	15	1	chasing
AxA	11_1	9	10	11	20	0	chasing
AxA	11_1	9	10	11	25	0	chasing
AxA	11_1	9	10	11	30	0	chasing
AxA	11_1	9	10	11	35	0	chasing
AxA	11_1	9	10	11	40	0	chasing
AxA	11_1	9	10	11	45	0	chasing
AxA	11_1	9	10	11	50	0	chasing
AxA	11_1	9	10	11	55	0	chasing
AxA	11_1	9	10	11	60	0	chasing
AxA	11_1	9	10	11	65	0	chasing
AxA	11_1	9	10	11	70	0	chasing
AxA	11_1	9	10	11	75	0	chasing
AxA	11_1	9	10	11	80	0	chasing
AxA	11_1	9	10	11	85	0	chasing
AxA	11_1	9	10	11	90	0	chasing
AxA	11_1	9	10	11	95	0	chasing
AxA	11_1	9	10	11	100	0	chasing
AxA	11_1	9	10	11	105	0	chasing
AxA	11_1	9	10	11	110	0	chasing
AxA	11_1	9	10	11	115	0	chasing

AxA	11_1	9	10	11	120	0	chasing
AxA	11_1	9	10	11	125	0	chasing
AxA	11_1	9	10	11	130	0	chasing
AxA	11_1	9	10	11	135	0	chasing
AxA	11_1	9	10	11	140	0	chasing
AxA	11_1	9	10	11	145	0	chasing
AxA	11_1	9	10	11	150	0	chasing
AxA	11_1	9	10	11	155	0	chasing
AxA	11_1	9	10	11	160	0	chasing
AxA	11_1	9	10	11	165	0	chasing
AxA	11_1	9	10	11	170	0	chasing
AxA	11_1	9	10	11	175	0	chasing
AxA	11_1	9	10	11	180	0	chasing
AxA	11_1	9	10	11	185	0	chasing
AxA	11_1	9	10	11	190	0	chasing
AxA	11_1	9	10	11	195	0	chasing
AxA	11_1	9	10	11	200	0	chasing
AxA	11_1	9	10	11	205	0	chasing
AxA	11_1	9	10	11	210	0	chasing
AxA	11_1	9	10	11	215	0	chasing
AxA	11_1	9	10	11	220	0	chasing
AxA	11_1	9	10	11	225	0	chasing
AxA	11_1	9	10	11	230	0	chasing
AxA	11_1	9	10	11	235	0	chasing
AxA	11_1	9	10	11	240	0	chasing
AxA	11_1	9	10	11	245	0	chasing
AxA	11_1	9	10	11	250	0	chasing
AxA	11_1	9	10	11	255	0	chasing
AxA	11_1	9	10	11	260	0	chasing
AxA	11_1	9	10	11	265	0	chasing
AxA	11_1	9	10	11	270	0	chasing
AxA	11_1	9	10	11	275	0	chasing
AxA	11_1	9	10	11	280	0	chasing
AxA	11_1	9	10	11	285	0	chasing
AxA	11_1	9	10	11	290	0	chasing
AxA	11_1	9	10	11	295	0	chasing
AxA	11_1	9	10	11	300	0	chasing
AxA	12_1	13	13	12	5	0	chasing
AxA	12_1	13	13	12	10	1	chasing
AxA	12_1	13	13	12	15	0	chasing
AxA	12_1	13	13	12	20	0	chasing
AxA	12_1	13	13	12	25	0	chasing
AxA	12_1	13	13	12	30	0	chasing
AxA	12_1	13	13	12	35	0	chasing
AxA	12_1	13	13	12	40	0	chasing
AxA	12_1	13	13	12	45	0	chasing
AxA	12_1	13	13	12	50	0	chasing
AxA	12_1	13	13	12	55	0	chasing

AxA	12_1	13	13	12	60	0	chasing
AxA	12_1	13	13	12	65	0	chasing
AxA	12_1	13	13	12	70	0	chasing
AxA	12_1	13	13	12	75	0	chasing
AxA	12_1	13	13	12	80	0	chasing
AxA	12_1	13	13	12	85	0	chasing
AxA	12_1	13	13	12	90	0	chasing
AxA	12_1	13	13	12	95	0	chasing
AxA	12_1	13	13	12	100	0	chasing
AxA	12_1	13	13	12	105	0	chasing
AxA	12_1	13	13	12	110	0	chasing
AxA	12_1	13	13	12	115	0	chasing
AxA	12_1	13	13	12	120	0	chasing
AxA	12_1	13	13	12	125	0	chasing
AxA	12_1	13	13	12	130	0	chasing
AxA	12_1	13	13	12	135	0	chasing
AxA	12_1	13	13	12	140	0	chasing
AxA	12_1	13	13	12	145	0	chasing
AxA	12_1	13	13	12	150	0	chasing
AxA	12_1	13	13	12	155	0	chasing
AxA	12_1	13	13	12	160	0	chasing
AxA	12_1	13	13	12	165	0	chasing
AxA	12_1	13	13	12	170	0	chasing
AxA	12_1	13	13	12	175	0	chasing
AxA	12_1	13	13	12	180	0	chasing
AxA	12_1	13	13	12	185	0	chasing
AxA	12_1	13	13	12	190	0	chasing
AxA	12_1	13	13	12	195	0	chasing
AxA	12_1	13	13	12	200	0	chasing
AxA	12_1	13	13	12	205	0	chasing
AxA	12_1	13	13	12	210	1	chasing
AxA	12_1	13	13	12	215	0	chasing
AxA	12_1	13	13	12	220	0	chasing
AxA	12_1	13	13	12	225	0	chasing
AxA	12_1	13	13	12	230	0	chasing
AxA	12_1	13	13	12	235	0	chasing
AxA	12_1	13	13	12	240	0	chasing
AxA	12_1	13	13	12	245	0	chasing
AxA	12_1	13	13	12	250	0	chasing
AxA	12_1	13	13	12	255	0	chasing
AxA	12_1	13	13	12	260	0	chasing
AxA	12_1	13	13	12	265	0	chasing
AxA	12_1	13	13	12	270	0	chasing
AxA	12_1	13	13	12	275	0	chasing
AxA	12_1	13	13	12	280	0	chasing
AxA	12_1	13	13	12	285	0	chasing
AxA	12_1	13	13	12	290	0	chasing
AxA	12_1	13	13	12	295	0	chasing

AxA	12_1	13	13	12	300	0	chasing
AxA	13_1	15	13	13	5	0	chasing
AxA	13_1	15	13	13	10	0	chasing
AxA	13_1	15	13	13	15	0	chasing
AxA	13_1	15	13	13	20	0	chasing
AxA	13_1	15	13	13	25	0	chasing
AxA	13_1	15	13	13	30	0	chasing
AxA	13_1	15	13	13	35	0	chasing
AxA	13_1	15	13	13	40	0	chasing
AxA	13_1	15	13	13	45	0	chasing
AxA	13_1	15	13	13	50	0	chasing
AxA	13_1	15	13	13	55	0	chasing
AxA	13_1	15	13	13	60	0	chasing
AxA	13_1	15	13	13	65	0	chasing
AxA	13_1	15	13	13	70	0	chasing
AxA	13_1	15	13	13	75	0	chasing
AxA	13_1	15	13	13	80	0	chasing
AxA	13_1	15	13	13	85	0	chasing
AxA	13_1	15	13	13	90	0	chasing
AxA	13_1	15	13	13	95	0	chasing
AxA	13_1	15	13	13	100	0	chasing
AxA	13_1	15	13	13	105	0	chasing
AxA	13_1	15	13	13	110	0	chasing
AxA	13_1	15	13	13	115	0	chasing
AxA	13_1	15	13	13	120	0	chasing
AxA	13_1	15	13	13	125	0	chasing
AxA	13_1	15	13	13	130	0	chasing
AxA	13_1	15	13	13	135	0	chasing
AxA	13_1	15	13	13	140	0	chasing
AxA	13_1	15	13	13	145	0	chasing
AxA	13_1	15	13	13	150	0	chasing
AxA	13_1	15	13	13	155	0	chasing
AxA	13_1	15	13	13	160	0	chasing
AxA	13_1	15	13	13	165	0	chasing
AxA	13_1	15	13	13	170	0	chasing
AxA	13_1	15	13	13	175	0	chasing
AxA	13_1	15	13	13	180	0	chasing
AxA	13_1	15	13	13	185	0	chasing
AxA	13_1	15	13	13	190	0	chasing
AxA	13_1	15	13	13	195	0	chasing
AxA	13_1	15	13	13	200	0	chasing
AxA	13_1	15	13	13	205	0	chasing
AxA	13_1	15	13	13	210	0	chasing
AxA	13_1	15	13	13	215	0	chasing
AxA	13_1	15	13	13	220	0	chasing
AxA	13_1	15	13	13	225	0	chasing
AxA	13_1	15	13	13	230	0	chasing
AxA	13_1	15	13	13	235	0	chasing

AxA	13_1	15	13	13	240	0	chasing
AxA	13_1	15	13	13	245	0	chasing
AxA	13_1	15	13	13	250	0	chasing
AxA	13_1	15	13	13	255	0	chasing
AxA	13_1	15	13	13	260	0	chasing
AxA	13_1	15	13	13	265	0	chasing
AxA	13_1	15	13	13	270	0	chasing
AxA	13_1	15	13	13	275	0	chasing
AxA	13_1	15	13	13	280	0	chasing
AxA	13_1	15	13	13	285	0	chasing
AxA	13_1	15	13	13	290	0	chasing
AxA	13_1	15	13	13	295	0	chasing
AxA	13_1	15	13	13	300	0	chasing
AxA	14_1	10	11	14	5	0	chasing
AxA	14_1	10	11	14	10	0	chasing
AxA	14_1	10	11	14	15	0	chasing
AxA	14_1	10	11	14	20	0	chasing
AxA	14_1	10	11	14	25	0	chasing
AxA	14_1	10	11	14	30	0	chasing
AxA	14_1	10	11	14	35	0	chasing
AxA	14_1	10	11	14	40	0	chasing
AxA	14_1	10	11	14	45	0	chasing
AxA	14_1	10	11	14	50	0	chasing
AxA	14_1	10	11	14	55	0	chasing
AxA	14_1	10	11	14	60	0	chasing
AxA	14_1	10	11	14	65	0	chasing
AxA	14_1	10	11	14	70	0	chasing
AxA	14_1	10	11	14	75	0	chasing
AxA	14_1	10	11	14	80	0	chasing
AxA	14_1	10	11	14	85	0	chasing
AxA	14_1	10	11	14	90	0	chasing
AxA	14_1	10	11	14	95	0	chasing
AxA	14_1	10	11	14	100	0	chasing
AxA	14_1	10	11	14	105	0	chasing
AxA	14_1	10	11	14	110	0	chasing
AxA	14_1	10	11	14	115	0	chasing
AxA	14_1	10	11	14	120	0	chasing
AxA	14_1	10	11	14	125	0	chasing
AxA	14_1	10	11	14	130	0	chasing
AxA	14_1	10	11	14	135	0	chasing
AxA	14_1	10	11	14	140	0	chasing
AxA	14_1	10	11	14	145	0	chasing
AxA	14_1	10	11	14	150	0	chasing
AxA	14_1	10	11	14	155	0	chasing
AxA	14_1	10	11	14	160	0	chasing
AxA	14_1	10	11	14	165	0	chasing
AxA	14_1	10	11	14	170	0	chasing
AxA	14_1	10	11	14	175	0	chasing

AxA	14_1	10	11	14	180	0	chasing
AxA	14_1	10	11	14	185	0	chasing
AxA	14_1	10	11	14	190	0	chasing
AxA	14_1	10	11	14	195	0	chasing
AxA	14_1	10	11	14	200	0	chasing
AxA	14_1	10	11	14	205	0	chasing
AxA	14_1	10	11	14	210	0	chasing
AxA	14_1	10	11	14	215	0	chasing
AxA	14_1	10	11	14	220	0	chasing
AxA	14_1	10	11	14	225	0	chasing
AxA	14_1	10	11	14	230	0	chasing
AxA	14_1	10	11	14	235	0	chasing
AxA	14_1	10	11	14	240	0	chasing
AxA	14_1	10	11	14	245	0	chasing
AxA	14_1	10	11	14	250	0	chasing
AxA	14_1	10	11	14	255	0	chasing
AxA	14_1	10	11	14	260	0	chasing
AxA	14_1	10	11	14	265	0	chasing
AxA	14_1	10	11	14	270	0	chasing
AxA	14_1	10	11	14	275	0	chasing
AxA	14_1	10	11	14	280	0	chasing
AxA	14_1	10	11	14	285	0	chasing
AxA	14_1	10	11	14	290	0	chasing
AxA	14_1	10	11	14	295	0	chasing
AxA	14_1	10	11	14	300	0	chasing
AxA	15_1	13	13	15	5	0	chasing
AxA	15_1	13	13	15	10	1	chasing
AxA	15_1	13	13	15	15	0	chasing
AxA	15_1	13	13	15	20	0	chasing
AxA	15_1	13	13	15	25	1	chasing
AxA	15_1	13	13	15	30	0	chasing
AxA	15_1	13	13	15	35	0	chasing
AxA	15_1	13	13	15	40	0	chasing
AxA	15_1	13	13	15	45	0	chasing
AxA	15_1	13	13	15	50	0	chasing
AxA	15_1	13	13	15	55	0	chasing
AxA	15_1	13	13	15	60	0	chasing
AxA	15_1	13	13	15	65	0	chasing
AxA	15_1	13	13	15	70	0	chasing
AxA	15_1	13	13	15	75	0	chasing
AxA	15_1	13	13	15	80	0	chasing
AxA	15_1	13	13	15	85	0	chasing
AxA	15_1	13	13	15	90	0	chasing
AxA	15_1	13	13	15	95	0	chasing
AxA	15_1	13	13	15	100	0	chasing
AxA	15_1	13	13	15	105	0	chasing
AxA	15_1	13	13	15	110	0	chasing
AxA	15_1	13	13	15	115	0	chasing

AxA	15_1	13	13	15	120	0	chasing
AxA	15_1	13	13	15	125	0	chasing
AxA	15_1	13	13	15	130	0	chasing
AxA	15_1	13	13	15	135	0	chasing
AxA	15_1	13	13	15	140	0	chasing
AxA	15_1	13	13	15	145	0	chasing
AxA	15_1	13	13	15	150	0	chasing
AxA	15_1	13	13	15	155	0	chasing
AxA	15_1	13	13	15	160	0	chasing
AxA	15_1	13	13	15	165	0	chasing
AxA	15_1	13	13	15	170	0	chasing
AxA	15_1	13	13	15	175	0	chasing
AxA	15_1	13	13	15	180	0	chasing
AxA	15_1	13	13	15	185	0	chasing
AxA	15_1	13	13	15	190	0	chasing
AxA	15_1	13	13	15	195	0	chasing
AxA	15_1	13	13	15	200	0	chasing
AxA	15_1	13	13	15	205	0	chasing
AxA	15_1	13	13	15	210	0	chasing
AxA	15_1	13	13	15	215	0	chasing
AxA	15_1	13	13	15	220	0	chasing
AxA	15_1	13	13	15	225	0	chasing
AxA	15_1	13	13	15	230	0	chasing
AxA	15_1	13	13	15	235	0	chasing
AxA	15_1	13	13	15	240	0	chasing
AxA	15_1	13	13	15	245	0	chasing
AxA	15_1	13	13	15	250	0	chasing
AxA	15_1	13	13	15	255	0	chasing
AxA	15_1	13	13	15	260	0	chasing
AxA	15_1	13	13	15	265	0	chasing
AxA	15_1	13	13	15	270	0	chasing
AxA	15_1	13	13	15	275	0	chasing
AxA	15_1	13	13	15	280	0	chasing
AxA	15_1	13	13	15	285	0	chasing
AxA	15_1	13	13	15	290	0	chasing
AxA	15_1	13	13	15	295	0	chasing
AxA	15_1	13	13	15	300	0	chasing
AxA	16_1	14	15	16	5	0	chasing
AxA	16_1	14	15	16	10	0	chasing
AxA	16_1	14	15	16	15	0	chasing
AxA	16_1	14	15	16	20	0	chasing
AxA	16_1	14	15	16	25	0	chasing
AxA	16_1	14	15	16	30	0	chasing
AxA	16_1	14	15	16	35	0	chasing
AxA	16_1	14	15	16	40	0	chasing
AxA	16_1	14	15	16	45	0	chasing
AxA	16_1	14	15	16	50	0	chasing
AxA	16_1	14	15	16	55	0	chasing

AxA	16_1	14	15	16	60	0	chasing
AxA	16_1	14	15	16	65	0	chasing
AxA	16_1	14	15	16	70	0	chasing
AxA	16_1	14	15	16	75	0	chasing
AxA	16_1	14	15	16	80	0	chasing
AxA	16_1	14	15	16	85	0	chasing
AxA	16_1	14	15	16	90	0	chasing
AxA	16_1	14	15	16	95	0	chasing
AxA	16_1	14	15	16	100	0	chasing
AxA	16_1	14	15	16	105	0	chasing
AxA	16_1	14	15	16	110	0	chasing
AxA	16_1	14	15	16	115	0	chasing
AxA	16_1	14	15	16	120	0	chasing
AxA	16_1	14	15	16	125	0	chasing
AxA	16_1	14	15	16	130	0	chasing
AxA	16_1	14	15	16	135	0	chasing
AxA	16_1	14	15	16	140	0	chasing
AxA	16_1	14	15	16	145	0	chasing
AxA	16_1	14	15	16	150	0	chasing
AxA	16_1	14	15	16	155	0	chasing
AxA	16_1	14	15	16	160	0	chasing
AxA	16_1	14	15	16	165	0	chasing
AxA	16_1	14	15	16	170	0	chasing
AxA	16_1	14	15	16	175	0	chasing
AxA	16_1	14	15	16	180	0	chasing
AxA	16_1	14	15	16	185	0	chasing
AxA	16_1	14	15	16	190	0	chasing
AxA	16_1	14	15	16	195	0	chasing
AxA	16_1	14	15	16	200	0	chasing
AxA	16_1	14	15	16	205	0	chasing
AxA	16_1	14	15	16	210	0	chasing
AxA	16_1	14	15	16	215	0	chasing
AxA	16_1	14	15	16	220	0	chasing
AxA	16_1	14	15	16	225	0	chasing
AxA	16_1	14	15	16	230	0	chasing
AxA	16_1	14	15	16	235	0	chasing
AxA	16_1	14	15	16	240	0	chasing
AxA	16_1	14	15	16	245	0	chasing
AxA	16_1	14	15	16	250	0	chasing
AxA	16_1	14	15	16	255	0	chasing
AxA	16_1	14	15	16	260	0	chasing
AxA	16_1	14	15	16	265	0	chasing
AxA	16_1	14	15	16	270	0	chasing
AxA	16_1	14	15	16	275	0	chasing
AxA	16_1	14	15	16	280	0	chasing
AxA	16_1	14	15	16	285	0	chasing
AxA	16_1	14	15	16	290	0	chasing
AxA	16_1	14	15	16	295	0	chasing

AxA	16_1	14	15	16	300	0	chasing
AxA	17_1	7	9	17	5	0	chasing
AxA	17_1	7	9	17	10	0	chasing
AxA	17_1	7	9	17	15	0	chasing
AxA	17_1	7	9	17	20	0	chasing
AxA	17_1	7	9	17	25	0	chasing
AxA	17_1	7	9	17	30	0	chasing
AxA	17_1	7	9	17	35	0	chasing
AxA	17_1	7	9	17	40	0	chasing
AxA	17_1	7	9	17	45	0	chasing
AxA	17_1	7	9	17	50	0	chasing
AxA	17_1	7	9	17	55	0	chasing
AxA	17_1	7	9	17	60	0	chasing
AxA	17_1	7	9	17	65	0	chasing
AxA	17_1	7	9	17	70	0	chasing
AxA	17_1	7	9	17	75	0	chasing
AxA	17_1	7	9	17	80	0	chasing
AxA	17_1	7	9	17	85	0	chasing
AxA	17_1	7	9	17	90	0	chasing
AxA	17_1	7	9	17	95	0	chasing
AxA	17_1	7	9	17	100	0	chasing
AxA	17_1	7	9	17	105	1	chasing
AxA	17_1	7	9	17	110	0	chasing
AxA	17_1	7	9	17	115	0	chasing
AxA	17_1	7	9	17	120	0	chasing
AxA	17_1	7	9	17	125	0	chasing
AxA	17_1	7	9	17	130	0	chasing
AxA	17_1	7	9	17	135	0	chasing
AxA	17_1	7	9	17	140	0	chasing
AxA	17_1	7	9	17	145	0	chasing
AxA	17_1	7	9	17	150	0	chasing
AxA	17_1	7	9	17	155	0	chasing
AxA	17_1	7	9	17	160	0	chasing
AxA	17_1	7	9	17	165	0	chasing
AxA	17_1	7	9	17	170	0	chasing
AxA	17_1	7	9	17	175	0	chasing
AxA	17_1	7	9	17	180	0	chasing
AxA	17_1	7	9	17	185	0	chasing
AxA	17_1	7	9	17	190	0	chasing
AxA	17_1	7	9	17	195	0	chasing
AxA	17_1	7	9	17	200	0	chasing
AxA	17_1	7	9	17	205	0	chasing
AxA	17_1	7	9	17	210	0	chasing
AxA	17_1	7	9	17	215	0	chasing
AxA	17_1	7	9	17	220	0	chasing
AxA	17_1	7	9	17	225	0	chasing
AxA	17_1	7	9	17	230	0	chasing
AxA	17_1	7	9	17	235	0	chasing

AxA	17_1	7	9	17	240	0	chasing
AxA	17_1	7	9	17	245	0	chasing
AxA	17_1	7	9	17	250	0	chasing
AxA	17_1	7	9	17	255	0	chasing
AxA	17_1	7	9	17	260	0	chasing
AxA	17_1	7	9	17	265	0	chasing
AxA	17_1	7	9	17	270	0	chasing
AxA	17_1	7	9	17	275	0	chasing
AxA	17_1	7	9	17	280	0	chasing
AxA	17_1	7	9	17	285	0	chasing
AxA	17_1	7	9	17	290	0	chasing
AxA	17_1	7	9	17	295	0	chasing
AxA	17_1	7	9	17	300	0	chasing
AxA	18_1	12	9	18	5	0	chasing
AxA	18_1	12	9	18	10	0	chasing
AxA	18_1	12	9	18	15	0	chasing
AxA	18_1	12	9	18	20	0	chasing
AxA	18_1	12	9	18	25	0	chasing
AxA	18_1	12	9	18	30	0	chasing
AxA	18_1	12	9	18	35	0	chasing
AxA	18_1	12	9	18	40	0	chasing
AxA	18_1	12	9	18	45	0	chasing
AxA	18_1	12	9	18	50	0	chasing
AxA	18_1	12	9	18	55	0	chasing
AxA	18_1	12	9	18	60	0	chasing
AxA	18_1	12	9	18	65	0	chasing
AxA	18_1	12	9	18	70	0	chasing
AxA	18_1	12	9	18	75	0	chasing
AxA	18_1	12	9	18	80	0	chasing
AxA	18_1	12	9	18	85	0	chasing
AxA	18_1	12	9	18	90	0	chasing
AxA	18_1	12	9	18	95	0	chasing
AxA	18_1	12	9	18	100	0	chasing
AxA	18_1	12	9	18	105	0	chasing
AxA	18_1	12	9	18	110	0	chasing
AxA	18_1	12	9	18	115	0	chasing
AxA	18_1	12	9	18	120	0	chasing
AxA	18_1	12	9	18	125	0	chasing
AxA	18_1	12	9	18	130	0	chasing
AxA	18_1	12	9	18	135	0	chasing
AxA	18_1	12	9	18	140	0	chasing
AxA	18_1	12	9	18	145	0	chasing
AxA	18_1	12	9	18	150	0	chasing
AxA	18_1	12	9	18	155	0	chasing
AxA	18_1	12	9	18	160	0	chasing
AxA	18_1	12	9	18	165	0	chasing
AxA	18_1	12	9	18	170	0	chasing
AxA	18_1	12	9	18	175	0	chasing

AxA	18_1	12	9	18	180	0	chasing
AxA	18_1	12	9	18	185	0	chasing
AxA	18_1	12	9	18	190	1	chasing
AxA	18_1	12	9	18	195	0	chasing
AxA	18_1	12	9	18	200	0	chasing
AxA	18_1	12	9	18	205	0	chasing
AxA	18_1	12	9	18	210	0	chasing
AxA	18_1	12	9	18	215	0	chasing
AxA	18_1	12	9	18	220	0	chasing
AxA	18_1	12	9	18	225	0	chasing
AxA	18_1	12	9	18	230	0	chasing
AxA	18_1	12	9	18	235	0	chasing
AxA	18_1	12	9	18	240	0	chasing
AxA	18_1	12	9	18	245	0	chasing
AxA	18_1	12	9	18	250	0	chasing
AxA	18_1	12	9	18	255	0	chasing
AxA	18_1	12	9	18	260	0	chasing
AxA	18_1	12	9	18	265	1	chasing
AxA	18_1	12	9	18	270	0	chasing
AxA	18_1	12	9	18	275	0	chasing
AxA	18_1	12	9	18	280	0	chasing
AxA	18_1	12	9	18	285	0	chasing
AxA	18_1	12	9	18	290	1	chasing
AxA	18_1	12	9	18	295	0	chasing
AxA	18_1	12	9	18	300	0	chasing
AxA	19_1	8	13	19	5	0	chasing
AxA	19_1	8	13	19	10	0	chasing
AxA	19_1	8	13	19	15	0	chasing
AxA	19_1	8	13	19	20	0	chasing
AxA	19_1	8	13	19	25	0	chasing
AxA	19_1	8	13	19	30	0	chasing
AxA	19_1	8	13	19	35	0	chasing
AxA	19_1	8	13	19	40	0	chasing
AxA	19_1	8	13	19	45	0	chasing
AxA	19_1	8	13	19	50	0	chasing
AxA	19_1	8	13	19	55	0	chasing
AxA	19_1	8	13	19	60	0	chasing
AxA	19_1	8	13	19	65	0	chasing
AxA	19_1	8	13	19	70	0	chasing
AxA	19_1	8	13	19	75	0	chasing
AxA	19_1	8	13	19	80	0	chasing
AxA	19_1	8	13	19	85	0	chasing
AxA	19_1	8	13	19	90	0	chasing
AxA	19_1	8	13	19	95	0	chasing
AxA	19_1	8	13	19	100	0	chasing
AxA	19_1	8	13	19	105	0	chasing
AxA	19_1	8	13	19	110	0	chasing
AxA	19_1	8	13	19	115	0	chasing

AxA	19_1	8	13	19	120	0	chasing
AxA	19_1	8	13	19	125	0	chasing
AxA	19_1	8	13	19	130	0	chasing
AxA	19_1	8	13	19	135	0	chasing
AxA	19_1	8	13	19	140	0	chasing
AxA	19_1	8	13	19	145	0	chasing
AxA	19_1	8	13	19	150	0	chasing
AxA	19_1	8	13	19	155	0	chasing
AxA	19_1	8	13	19	160	0	chasing
AxA	19_1	8	13	19	165	0	chasing
AxA	19_1	8	13	19	170	0	chasing
AxA	19_1	8	13	19	175	0	chasing
AxA	19_1	8	13	19	180	0	chasing
AxA	19_1	8	13	19	185	0	chasing
AxA	19_1	8	13	19	190	0	chasing
AxA	19_1	8	13	19	195	0	chasing
AxA	19_1	8	13	19	200	0	chasing
AxA	19_1	8	13	19	205	0	chasing
AxA	19_1	8	13	19	210	0	chasing
AxA	19_1	8	13	19	215	0	chasing
AxA	19_1	8	13	19	220	0	chasing
AxA	19_1	8	13	19	225	0	chasing
AxA	19_1	8	13	19	230	0	chasing
AxA	19_1	8	13	19	235	0	chasing
AxA	19_1	8	13	19	240	0	chasing
AxA	19_1	8	13	19	245	0	chasing
AxA	19_1	8	13	19	250	0	chasing
AxA	19_1	8	13	19	255	0	chasing
AxA	19_1	8	13	19	260	0	chasing
AxA	19_1	8	13	19	265	0	chasing
AxA	19_1	8	13	19	270	0	chasing
AxA	19_1	8	13	19	275	0	chasing
AxA	19_1	8	13	19	280	0	chasing
AxA	19_1	8	13	19	285	0	chasing
AxA	19_1	8	13	19	290	0	chasing
AxA	19_1	8	13	19	295	0	chasing
AxA	19_1	8	13	19	300	0	chasing
AxA	20_1	15	12	20	5	0	chasing
AxA	20_1	15	12	20	10	0	chasing
AxA	20_1	15	12	20	15	0	chasing
AxA	20_1	15	12	20	20	0	chasing
AxA	20_1	15	12	20	25	0	chasing
AxA	20_1	15	12	20	30	0	chasing
AxA	20_1	15	12	20	35	0	chasing
AxA	20_1	15	12	20	40	0	chasing
AxA	20_1	15	12	20	45	0	chasing
AxA	20_1	15	12	20	50	0	chasing
AxA	20_1	15	12	20	55	0	chasing

AxA	20_1	15	12	20	60	0	chasing
AxA	20_1	15	12	20	65	0	chasing
AxA	20_1	15	12	20	70	0	chasing
AxA	20_1	15	12	20	75	0	chasing
AxA	20_1	15	12	20	80	0	chasing
AxA	20_1	15	12	20	85	0	chasing
AxA	20_1	15	12	20	90	0	chasing
AxA	20_1	15	12	20	95	0	chasing
AxA	20_1	15	12	20	100	0	chasing
AxA	20_1	15	12	20	105	0	chasing
AxA	20_1	15	12	20	110	0	chasing
AxA	20_1	15	12	20	115	0	chasing
AxA	20_1	15	12	20	120	0	chasing
AxA	20_1	15	12	20	125	0	chasing
AxA	20_1	15	12	20	130	0	chasing
AxA	20_1	15	12	20	135	0	chasing
AxA	20_1	15	12	20	140	0	chasing
AxA	20_1	15	12	20	145	0	chasing
AxA	20_1	15	12	20	150	0	chasing
AxA	20_1	15	12	20	155	0	chasing
AxA	20_1	15	12	20	160	0	chasing
AxA	20_1	15	12	20	165	0	chasing
AxA	20_1	15	12	20	170	0	chasing
AxA	20_1	15	12	20	175	0	chasing
AxA	20_1	15	12	20	180	0	chasing
AxA	20_1	15	12	20	185	0	chasing
AxA	20_1	15	12	20	190	0	chasing
AxA	20_1	15	12	20	195	0	chasing
AxA	20_1	15	12	20	200	0	chasing
AxA	20_1	15	12	20	205	0	chasing
AxA	20_1	15	12	20	210	0	chasing
AxA	20_1	15	12	20	215	0	chasing
AxA	20_1	15	12	20	220	0	chasing
AxA	20_1	15	12	20	225	0	chasing
AxA	20_1	15	12	20	230	0	chasing
AxA	20_1	15	12	20	235	0	chasing
AxA	20_1	15	12	20	240	0	chasing
AxA	20_1	15	12	20	245	0	chasing
AxA	20_1	15	12	20	250	0	chasing
AxA	20_1	15	12	20	255	0	chasing
AxA	20_1	15	12	20	260	0	chasing
AxA	20_1	15	12	20	265	0	chasing
AxA	20_1	15	12	20	270	0	chasing
AxA	20_1	15	12	20	275	0	chasing
AxA	20_1	15	12	20	280	0	chasing
AxA	20_1	15	12	20	285	0	chasing
AxA	20_1	15	12	20	290	0	chasing
AxA	20_1	15	12	20	295	0	chasing

AxA	20_1	15	12	20	300	0	chasing
CxC	1_1	10	7	39	5	0	chasing
CxC	1_1	10	7	39	10	0	chasing
CxC	1_1	10	7	39	15	0	chasing
CxC	1_1	10	7	39	20	0	chasing
CxC	1_1	10	7	39	25	0	chasing
CxC	1_1	10	7	39	30	0	chasing
CxC	1_1	10	7	39	35	1	chasing
CxC	1_1	10	7	39	40	0	chasing
CxC	1_1	10	7	39	45	0	chasing
CxC	1_1	10	7	39	50	0	chasing
CxC	1_1	10	7	39	55	0	chasing
CxC	1_1	10	7	39	60	0	chasing
CxC	1_1	10	7	39	65	0	chasing
CxC	1_1	10	7	39	70	0	chasing
CxC	1_1	10	7	39	75	0	chasing
CxC	1_1	10	7	39	80	0	chasing
CxC	1_1	10	7	39	85	0	chasing
CxC	1_1	10	7	39	90	0	chasing
CxC	1_1	10	7	39	95	0	chasing
CxC	1_1	10	7	39	100	0	chasing
CxC	1_1	10	7	39	105	0	chasing
CxC	1_1	10	7	39	110	0	chasing
CxC	1_1	10	7	39	115	0	chasing
CxC	1_1	10	7	39	120	0	chasing
CxC	1_1	10	7	39	125	0	chasing
CxC	1_1	10	7	39	130	0	chasing
CxC	1_1	10	7	39	135	0	chasing
CxC	1_1	10	7	39	140	0	chasing
CxC	1_1	10	7	39	145	0	chasing
CxC	1_1	10	7	39	150	0	chasing
CxC	1_1	10	7	39	155	0	chasing
CxC	1_1	10	7	39	160	0	chasing
CxC	1_1	10	7	39	165	0	chasing
CxC	1_1	10	7	39	170	0	chasing
CxC	1_1	10	7	39	175	0	chasing
CxC	1_1	10	7	39	180	0	chasing
CxC	1_1	10	7	39	185	0	chasing
CxC	1_1	10	7	39	190	0	chasing
CxC	1_1	10	7	39	195	0	chasing
CxC	1_1	10	7	39	200	1	chasing
CxC	1_1	10	7	39	205	0	chasing
CxC	1_1	10	7	39	210	1	chasing
CxC	1_1	10	7	39	215	0	chasing
CxC	1_1	10	7	39	220	0	chasing
CxC	1_1	10	7	39	225	0	chasing
CxC	1_1	10	7	39	230	0	chasing
CxC	1_1	10	7	39	235	0	chasing

CxC	1_1	10	7	39	240	0	chasing
CxC	1_1	10	7	39	245	0	chasing
CxC	1_1	10	7	39	250	0	chasing
CxC	1_1	10	7	39	255	0	chasing
CxC	1_1	10	7	39	260	0	chasing
CxC	1_1	10	7	39	265	0	chasing
CxC	1_1	10	7	39	270	0	chasing
CxC	1_1	10	7	39	275	0	chasing
CxC	1_1	10	7	39	280	0	chasing
CxC	1_1	10	7	39	285	0	chasing
CxC	1_1	10	7	39	290	0	chasing
CxC	1_1	10	7	39	295	0	chasing
CxC	1_1	10	7	39	300	0	chasing
CxC	2_1	9	10	40	5	0	chasing
CxC	2_1	9	10	40	10	0	chasing
CxC	2_1	9	10	40	15	0	chasing
CxC	2_1	9	10	40	20	0	chasing
CxC	2_1	9	10	40	25	0	chasing
CxC	2_1	9	10	40	30	0	chasing
CxC	2_1	9	10	40	35	0	chasing
CxC	2_1	9	10	40	40	0	chasing
CxC	2_1	9	10	40	45	0	chasing
CxC	2_1	9	10	40	50	0	chasing
CxC	2_1	9	10	40	55	0	chasing
CxC	2_1	9	10	40	60	0	chasing
CxC	2_1	9	10	40	65	1	chasing
CxC	2_1	9	10	40	70	0	chasing
CxC	2_1	9	10	40	75	0	chasing
CxC	2_1	9	10	40	80	0	chasing
CxC	2_1	9	10	40	85	0	chasing
CxC	2_1	9	10	40	90	0	chasing
CxC	2_1	9	10	40	95	0	chasing
CxC	2_1	9	10	40	100	0	chasing
CxC	2_1	9	10	40	105	0	chasing
CxC	2_1	9	10	40	110	0	chasing
CxC	2_1	9	10	40	115	0	chasing
CxC	2_1	9	10	40	120	0	chasing
CxC	2_1	9	10	40	125	0	chasing
CxC	2_1	9	10	40	130	0	chasing
CxC	2_1	9	10	40	135	0	chasing
CxC	2_1	9	10	40	140	0	chasing
CxC	2_1	9	10	40	145	0	chasing
CxC	2_1	9	10	40	150	0	chasing
CxC	2_1	9	10	40	155	0	chasing
CxC	2_1	9	10	40	160	0	chasing
CxC	2_1	9	10	40	165	0	chasing
CxC	2_1	9	10	40	170	0	chasing
CxC	2_1	9	10	40	175	0	chasing

CxC	2_1	9	10	40	180	0	chasing
CxC	2_1	9	10	40	185	0	chasing
CxC	2_1	9	10	40	190	0	chasing
CxC	2_1	9	10	40	195	0	chasing
CxC	2_1	9	10	40	200	0	chasing
CxC	2_1	9	10	40	205	1	chasing
CxC	2_1	9	10	40	210	0	chasing
CxC	2_1	9	10	40	215	0	chasing
CxC	2_1	9	10	40	220	0	chasing
CxC	2_1	9	10	40	225	0	chasing
CxC	2_1	9	10	40	230	0	chasing
CxC	2_1	9	10	40	235	0	chasing
CxC	2_1	9	10	40	240	0	chasing
CxC	2_1	9	10	40	245	0	chasing
CxC	2_1	9	10	40	250	0	chasing
CxC	2_1	9	10	40	255	0	chasing
CxC	2_1	9	10	40	260	0	chasing
CxC	2_1	9	10	40	265	0	chasing
CxC	2_1	9	10	40	270	0	chasing
CxC	2_1	9	10	40	275	0	chasing
CxC	2_1	9	10	40	280	0	chasing
CxC	2_1	9	10	40	285	0	chasing
CxC	2_1	9	10	40	290	0	chasing
CxC	2_1	9	10	40	295	1	chasing
CxC	2_1	9	10	40	300	1	chasing
CxC	3_1	7	8	41	5	0	chasing
CxC	3_1	7	8	41	10	0	chasing
CxC	3_1	7	8	41	15	0	chasing
CxC	3_1	7	8	41	20	1	chasing
CxC	3_1	7	8	41	25	1	chasing
CxC	3_1	7	8	41	30	1	chasing
CxC	3_1	7	8	41	35	0	chasing
CxC	3_1	7	8	41	40	0	chasing
CxC	3_1	7	8	41	45	0	chasing
CxC	3_1	7	8	41	50	0	chasing
CxC	3_1	7	8	41	55	0	chasing
CxC	3_1	7	8	41	60	0	chasing
CxC	3_1	7	8	41	65	0	chasing
CxC	3_1	7	8	41	70	0	chasing
CxC	3_1	7	8	41	75	0	chasing
CxC	3_1	7	8	41	80	0	chasing
CxC	3_1	7	8	41	85	0	chasing
CxC	3_1	7	8	41	90	0	chasing
CxC	3_1	7	8	41	95	0	chasing
CxC	3_1	7	8	41	100	0	chasing
CxC	3_1	7	8	41	105	1	chasing
CxC	3_1	7	8	41	110	0	chasing
CxC	3_1	7	8	41	115	0	chasing

CxC	3_1	7	8	41	120	0	chasing
CxC	3_1	7	8	41	125	0	chasing
CxC	3_1	7	8	41	130	0	chasing
CxC	3_1	7	8	41	135	0	chasing
CxC	3_1	7	8	41	140	0	chasing
CxC	3_1	7	8	41	145	0	chasing
CxC	3_1	7	8	41	150	0	chasing
CxC	3_1	7	8	41	155	0	chasing
CxC	3_1	7	8	41	160	1	chasing
CxC	3_1	7	8	41	165	0	chasing
CxC	3_1	7	8	41	170	0	chasing
CxC	3_1	7	8	41	175	0	chasing
CxC	3_1	7	8	41	180	0	chasing
CxC	3_1	7	8	41	185	0	chasing
CxC	3_1	7	8	41	190	0	chasing
CxC	3_1	7	8	41	195	0	chasing
CxC	3_1	7	8	41	200	0	chasing
CxC	3_1	7	8	41	205	0	chasing
CxC	3_1	7	8	41	210	0	chasing
CxC	3_1	7	8	41	215	0	chasing
CxC	3_1	7	8	41	220	0	chasing
CxC	3_1	7	8	41	225	0	chasing
CxC	3_1	7	8	41	230	0	chasing
CxC	3_1	7	8	41	235	0	chasing
CxC	3_1	7	8	41	240	0	chasing
CxC	3_1	7	8	41	245	0	chasing
CxC	3_1	7	8	41	250	1	chasing
CxC	3_1	7	8	41	255	0	chasing
CxC	3_1	7	8	41	260	0	chasing
CxC	3_1	7	8	41	265	0	chasing
CxC	3_1	7	8	41	270	0	chasing
CxC	3_1	7	8	41	275	0	chasing
CxC	3_1	7	8	41	280	0	chasing
CxC	3_1	7	8	41	285	0	chasing
CxC	3_1	7	8	41	290	0	chasing
CxC	3_1	7	8	41	295	0	chasing
CxC	3_1	7	8	41	300	0	chasing
CxC	4_1	9	8	42	5	0	chasing
CxC	4_1	9	8	42	10	0	chasing
CxC	4_1	9	8	42	15	0	chasing
CxC	4_1	9	8	42	20	0	chasing
CxC	4_1	9	8	42	25	0	chasing
CxC	4_1	9	8	42	30	0	chasing
CxC	4_1	9	8	42	35	0	chasing
CxC	4_1	9	8	42	40	0	chasing
CxC	4_1	9	8	42	45	0	chasing
CxC	4_1	9	8	42	50	1	chasing
CxC	4_1	9	8	42	55	0	chasing

CxC	4_1	9	8	42	60	0	chasing
CxC	4_1	9	8	42	65	0	chasing
CxC	4_1	9	8	42	70	0	chasing
CxC	4_1	9	8	42	75	0	chasing
CxC	4_1	9	8	42	80	0	chasing
CxC	4_1	9	8	42	85	0	chasing
CxC	4_1	9	8	42	90	0	chasing
CxC	4_1	9	8	42	95	0	chasing
CxC	4_1	9	8	42	100	0	chasing
CxC	4_1	9	8	42	105	0	chasing
CxC	4_1	9	8	42	110	0	chasing
CxC	4_1	9	8	42	115	0	chasing
CxC	4_1	9	8	42	120	0	chasing
CxC	4_1	9	8	42	125	0	chasing
CxC	4_1	9	8	42	130	0	chasing
CxC	4_1	9	8	42	135	0	chasing
CxC	4_1	9	8	42	140	0	chasing
CxC	4_1	9	8	42	145	1	chasing
CxC	4_1	9	8	42	150	0	chasing
CxC	4_1	9	8	42	155	0	chasing
CxC	4_1	9	8	42	160	0	chasing
CxC	4_1	9	8	42	165	0	chasing
CxC	4_1	9	8	42	170	0	chasing
CxC	4_1	9	8	42	175	0	chasing
CxC	4_1	9	8	42	180	0	chasing
CxC	4_1	9	8	42	185	0	chasing
CxC	4_1	9	8	42	190	0	chasing
CxC	4_1	9	8	42	195	0	chasing
CxC	4_1	9	8	42	200	0	chasing
CxC	4_1	9	8	42	205	0	chasing
CxC	4_1	9	8	42	210	0	chasing
CxC	4_1	9	8	42	215	0	chasing
CxC	4_1	9	8	42	220	0	chasing
CxC	4_1	9	8	42	225	0	chasing
CxC	4_1	9	8	42	230	0	chasing
CxC	4_1	9	8	42	235	0	chasing
CxC	4_1	9	8	42	240	0	chasing
CxC	4_1	9	8	42	245	0	chasing
CxC	4_1	9	8	42	250	0	chasing
CxC	4_1	9	8	42	255	0	chasing
CxC	4_1	9	8	42	260	0	chasing
CxC	4_1	9	8	42	265	0	chasing
CxC	4_1	9	8	42	270	0	chasing
CxC	4_1	9	8	42	275	0	chasing
CxC	4_1	9	8	42	280	0	chasing
CxC	4_1	9	8	42	285	0	chasing
CxC	4_1	9	8	42	290	0	chasing
CxC	4_1	9	8	42	295	0	chasing

CxC	4_1	9	8	42	300	0	chasing
CxC	5_1	8	8	43	5	0	chasing
CxC	5_1	8	8	43	10	0	chasing
CxC	5_1	8	8	43	15	0	chasing
CxC	5_1	8	8	43	20	0	chasing
CxC	5_1	8	8	43	25	0	chasing
CxC	5_1	8	8	43	30	0	chasing
CxC	5_1	8	8	43	35	0	chasing
CxC	5_1	8	8	43	40	0	chasing
CxC	5_1	8	8	43	45	0	chasing
CxC	5_1	8	8	43	50	0	chasing
CxC	5_1	8	8	43	55	0	chasing
CxC	5_1	8	8	43	60	0	chasing
CxC	5_1	8	8	43	65	0	chasing
CxC	5_1	8	8	43	70	1	chasing
CxC	5_1	8	8	43	75	0	chasing
CxC	5_1	8	8	43	80	0	chasing
CxC	5_1	8	8	43	85	0	chasing
CxC	5_1	8	8	43	90	0	chasing
CxC	5_1	8	8	43	95	0	chasing
CxC	5_1	8	8	43	100	0	chasing
CxC	5_1	8	8	43	105	0	chasing
CxC	5_1	8	8	43	110	0	chasing
CxC	5_1	8	8	43	115	0	chasing
CxC	5_1	8	8	43	120	0	chasing
CxC	5_1	8	8	43	125	0	chasing
CxC	5_1	8	8	43	130	0	chasing
CxC	5_1	8	8	43	135	0	chasing
CxC	5_1	8	8	43	140	0	chasing
CxC	5_1	8	8	43	145	0	chasing
CxC	5_1	8	8	43	150	0	chasing
CxC	5_1	8	8	43	155	0	chasing
CxC	5_1	8	8	43	160	0	chasing
CxC	5_1	8	8	43	165	0	chasing
CxC	5_1	8	8	43	170	0	chasing
CxC	5_1	8	8	43	175	0	chasing
CxC	5_1	8	8	43	180	0	chasing
CxC	5_1	8	8	43	185	0	chasing
CxC	5_1	8	8	43	190	0	chasing
CxC	5_1	8	8	43	195	0	chasing
CxC	5_1	8	8	43	200	0	chasing
CxC	5_1	8	8	43	205	0	chasing
CxC	5_1	8	8	43	210	1	chasing
CxC	5_1	8	8	43	215	0	chasing
CxC	5_1	8	8	43	220	0	chasing
CxC	5_1	8	8	43	225	0	chasing
CxC	5_1	8	8	43	230	0	chasing
CxC	5_1	8	8	43	235	0	chasing

CxC	5_1	8	8	43	240	0	chasing
CxC	5_1	8	8	43	245	0	chasing
CxC	5_1	8	8	43	250	0	chasing
CxC	5_1	8	8	43	255	0	chasing
CxC	5_1	8	8	43	260	0	chasing
CxC	5_1	8	8	43	265	0	chasing
CxC	5_1	8	8	43	270	0	chasing
CxC	5_1	8	8	43	275	0	chasing
CxC	5_1	8	8	43	280	0	chasing
CxC	5_1	8	8	43	285	0	chasing
CxC	5_1	8	8	43	290	0	chasing
CxC	5_1	8	8	43	295	0	chasing
CxC	5_1	8	8	43	300	0	chasing
CxC	6_1	8	9	44	5	0	chasing
CxC	6_1	8	9	44	10	0	chasing
CxC	6_1	8	9	44	15	0	chasing
CxC	6_1	8	9	44	20	0	chasing
CxC	6_1	8	9	44	25	0	chasing
CxC	6_1	8	9	44	30	0	chasing
CxC	6_1	8	9	44	35	0	chasing
CxC	6_1	8	9	44	40	0	chasing
CxC	6_1	8	9	44	45	0	chasing
CxC	6_1	8	9	44	50	0	chasing
CxC	6_1	8	9	44	55	0	chasing
CxC	6_1	8	9	44	60	0	chasing
CxC	6_1	8	9	44	65	0	chasing
CxC	6_1	8	9	44	70	0	chasing
CxC	6_1	8	9	44	75	0	chasing
CxC	6_1	8	9	44	80	0	chasing
CxC	6_1	8	9	44	85	0	chasing
CxC	6_1	8	9	44	90	0	chasing
CxC	6_1	8	9	44	95	0	chasing
CxC	6_1	8	9	44	100	0	chasing
CxC	6_1	8	9	44	105	0	chasing
CxC	6_1	8	9	44	110	0	chasing
CxC	6_1	8	9	44	115	0	chasing
CxC	6_1	8	9	44	120	1	chasing
CxC	6_1	8	9	44	125	0	chasing
CxC	6_1	8	9	44	130	0	chasing
CxC	6_1	8	9	44	135	0	chasing
CxC	6_1	8	9	44	140	0	chasing
CxC	6_1	8	9	44	145	0	chasing
CxC	6_1	8	9	44	150	0	chasing
CxC	6_1	8	9	44	155	0	chasing
CxC	6_1	8	9	44	160	0	chasing
CxC	6_1	8	9	44	165	0	chasing
CxC	6_1	8	9	44	170	0	chasing
CxC	6_1	8	9	44	175	0	chasing

CxC	6_1	8	9	44	180	0	chasing
CxC	6_1	8	9	44	185	0	chasing
CxC	6_1	8	9	44	190	0	chasing
CxC	6_1	8	9	44	195	0	chasing
CxC	6_1	8	9	44	200	0	chasing
CxC	6_1	8	9	44	205	0	chasing
CxC	6_1	8	9	44	210	0	chasing
CxC	6_1	8	9	44	215	0	chasing
CxC	6_1	8	9	44	220	0	chasing
CxC	6_1	8	9	44	225	0	chasing
CxC	6_1	8	9	44	230	0	chasing
CxC	6_1	8	9	44	235	0	chasing
CxC	6_1	8	9	44	240	0	chasing
CxC	6_1	8	9	44	245	0	chasing
CxC	6_1	8	9	44	250	0	chasing
CxC	6_1	8	9	44	255	0	chasing
CxC	6_1	8	9	44	260	0	chasing
CxC	6_1	8	9	44	265	0	chasing
CxC	6_1	8	9	44	270	0	chasing
CxC	6_1	8	9	44	275	0	chasing
CxC	6_1	8	9	44	280	0	chasing
CxC	6_1	8	9	44	285	0	chasing
CxC	6_1	8	9	44	290	0	chasing
CxC	6_1	8	9	44	295	0	chasing
CxC	6_1	8	9	44	300	0	chasing
CxC	7_1	7	6	45	5	0	chasing
CxC	7_1	7	6	45	10	0	chasing
CxC	7_1	7	6	45	15	0	chasing
CxC	7_1	7	6	45	20	0	chasing
CxC	7_1	7	6	45	25	0	chasing
CxC	7_1	7	6	45	30	0	chasing
CxC	7_1	7	6	45	35	0	chasing
CxC	7_1	7	6	45	40	0	chasing
CxC	7_1	7	6	45	45	0	chasing
CxC	7_1	7	6	45	50	0	chasing
CxC	7_1	7	6	45	55	0	chasing
CxC	7_1	7	6	45	60	0	chasing
CxC	7_1	7	6	45	65	0	chasing
CxC	7_1	7	6	45	70	0	chasing
CxC	7_1	7	6	45	75	0	chasing
CxC	7_1	7	6	45	80	0	chasing
CxC	7_1	7	6	45	85	1	chasing
CxC	7_1	7	6	45	90	0	chasing
CxC	7_1	7	6	45	95	0	chasing
CxC	7_1	7	6	45	100	0	chasing
CxC	7_1	7	6	45	105	1	chasing
CxC	7_1	7	6	45	110	0	chasing
CxC	7_1	7	6	45	115	0	chasing

CxC	7_1	7	6	45	120	0	chasing
CxC	7_1	7	6	45	125	0	chasing
CxC	7_1	7	6	45	130	0	chasing
CxC	7_1	7	6	45	135	0	chasing
CxC	7_1	7	6	45	140	0	chasing
CxC	7_1	7	6	45	145	0	chasing
CxC	7_1	7	6	45	150	0	chasing
CxC	7_1	7	6	45	155	0	chasing
CxC	7_1	7	6	45	160	0	chasing
CxC	7_1	7	6	45	165	0	chasing
CxC	7_1	7	6	45	170	0	chasing
CxC	7_1	7	6	45	175	0	chasing
CxC	7_1	7	6	45	180	0	chasing
CxC	7_1	7	6	45	185	0	chasing
CxC	7_1	7	6	45	190	0	chasing
CxC	7_1	7	6	45	195	0	chasing
CxC	7_1	7	6	45	200	0	chasing
CxC	7_1	7	6	45	205	0	chasing
CxC	7_1	7	6	45	210	0	chasing
CxC	7_1	7	6	45	215	0	chasing
CxC	7_1	7	6	45	220	0	chasing
CxC	7_1	7	6	45	225	0	chasing
CxC	7_1	7	6	45	230	0	chasing
CxC	7_1	7	6	45	235	0	chasing
CxC	7_1	7	6	45	240	0	chasing
CxC	7_1	7	6	45	245	0	chasing
CxC	7_1	7	6	45	250	0	chasing
CxC	7_1	7	6	45	255	0	chasing
CxC	7_1	7	6	45	260	1	chasing
CxC	7_1	7	6	45	265	0	chasing
CxC	7_1	7	6	45	270	0	chasing
CxC	7_1	7	6	45	275	0	chasing
CxC	7_1	7	6	45	280	0	chasing
CxC	7_1	7	6	45	285	0	chasing
CxC	7_1	7	6	45	290	0	chasing
CxC	7_1	7	6	45	295	0	chasing
CxC	7_1	7	6	45	300	0	chasing
CxC	8_1	7	8	46	5	0	chasing
CxC	8_1	7	8	46	10	0	chasing
CxC	8_1	7	8	46	15	0	chasing
CxC	8_1	7	8	46	20	1	chasing
CxC	8_1	7	8	46	25	0	chasing
CxC	8_1	7	8	46	30	0	chasing
CxC	8_1	7	8	46	35	0	chasing
CxC	8_1	7	8	46	40	0	chasing
CxC	8_1	7	8	46	45	0	chasing
CxC	8_1	7	8	46	50	1	chasing
CxC	8_1	7	8	46	55	0	chasing

CxC	8_1	7	8	46	60	0	chasing
CxC	8_1	7	8	46	65	0	chasing
CxC	8_1	7	8	46	70	0	chasing
CxC	8_1	7	8	46	75	0	chasing
CxC	8_1	7	8	46	80	0	chasing
CxC	8_1	7	8	46	85	0	chasing
CxC	8_1	7	8	46	90	0	chasing
CxC	8_1	7	8	46	95	0	chasing
CxC	8_1	7	8	46	100	0	chasing
CxC	8_1	7	8	46	105	0	chasing
CxC	8_1	7	8	46	110	0	chasing
CxC	8_1	7	8	46	115	0	chasing
CxC	8_1	7	8	46	120	0	chasing
CxC	8_1	7	8	46	125	0	chasing
CxC	8_1	7	8	46	130	0	chasing
CxC	8_1	7	8	46	135	0	chasing
CxC	8_1	7	8	46	140	0	chasing
CxC	8_1	7	8	46	145	0	chasing
CxC	8_1	7	8	46	150	0	chasing
CxC	8_1	7	8	46	155	0	chasing
CxC	8_1	7	8	46	160	0	chasing
CxC	8_1	7	8	46	165	0	chasing
CxC	8_1	7	8	46	170	0	chasing
CxC	8_1	7	8	46	175	0	chasing
CxC	8_1	7	8	46	180	0	chasing
CxC	8_1	7	8	46	185	0	chasing
CxC	8_1	7	8	46	190	0	chasing
CxC	8_1	7	8	46	195	0	chasing
CxC	8_1	7	8	46	200	0	chasing
CxC	8_1	7	8	46	205	0	chasing
CxC	8_1	7	8	46	210	0	chasing
CxC	8_1	7	8	46	215	0	chasing
CxC	8_1	7	8	46	220	0	chasing
CxC	8_1	7	8	46	225	0	chasing
CxC	8_1	7	8	46	230	0	chasing
CxC	8_1	7	8	46	235	0	chasing
CxC	8_1	7	8	46	240	0	chasing
CxC	8_1	7	8	46	245	0	chasing
CxC	8_1	7	8	46	250	0	chasing
CxC	8_1	7	8	46	255	0	chasing
CxC	8_1	7	8	46	260	0	chasing
CxC	8_1	7	8	46	265	0	chasing
CxC	8_1	7	8	46	270	0	chasing
CxC	8_1	7	8	46	275	0	chasing
CxC	8_1	7	8	46	280	0	chasing
CxC	8_1	7	8	46	285	1	chasing
CxC	8_1	7	8	46	290	0	chasing
CxC	8_1	7	8	46	295	0	chasing

CxC	8_1	7	8	46	300	0	chasing
CxC	9_1	9	7	47	5	0	chasing
CxC	9_1	9	7	47	10	0	chasing
CxC	9_1	9	7	47	15	0	chasing
CxC	9_1	9	7	47	20	0	chasing
CxC	9_1	9	7	47	25	0	chasing
CxC	9_1	9	7	47	30	0	chasing
CxC	9_1	9	7	47	35	0	chasing
CxC	9_1	9	7	47	40	0	chasing
CxC	9_1	9	7	47	45	0	chasing
CxC	9_1	9	7	47	50	0	chasing
CxC	9_1	9	7	47	55	0	chasing
CxC	9_1	9	7	47	60	0	chasing
CxC	9_1	9	7	47	65	1	chasing
CxC	9_1	9	7	47	70	0	chasing
CxC	9_1	9	7	47	75	0	chasing
CxC	9_1	9	7	47	80	0	chasing
CxC	9_1	9	7	47	85	0	chasing
CxC	9_1	9	7	47	90	1	chasing
CxC	9_1	9	7	47	95	1	chasing
CxC	9_1	9	7	47	100	0	chasing
CxC	9_1	9	7	47	105	0	chasing
CxC	9_1	9	7	47	110	0	chasing
CxC	9_1	9	7	47	115	0	chasing
CxC	9_1	9	7	47	120	0	chasing
CxC	9_1	9	7	47	125	0	chasing
CxC	9_1	9	7	47	130	0	chasing
CxC	9_1	9	7	47	135	0	chasing
CxC	9_1	9	7	47	140	0	chasing
CxC	9_1	9	7	47	145	0	chasing
CxC	9_1	9	7	47	150	0	chasing
CxC	9_1	9	7	47	155	0	chasing
CxC	9_1	9	7	47	160	0	chasing
CxC	9_1	9	7	47	165	0	chasing
CxC	9_1	9	7	47	170	0	chasing
CxC	9_1	9	7	47	175	0	chasing
CxC	9_1	9	7	47	180	0	chasing
CxC	9_1	9	7	47	185	0	chasing
CxC	9_1	9	7	47	190	0	chasing
CxC	9_1	9	7	47	195	0	chasing
CxC	9_1	9	7	47	200	0	chasing
CxC	9_1	9	7	47	205	0	chasing
CxC	9_1	9	7	47	210	0	chasing
CxC	9_1	9	7	47	215	0	chasing
CxC	9_1	9	7	47	220	0	chasing
CxC	9_1	9	7	47	225	0	chasing
CxC	9_1	9	7	47	230	0	chasing
CxC	9_1	9	7	47	235	0	chasing

CxC	9_1	9	7	47	240	0	chasing
CxC	9_1	9	7	47	245	0	chasing
CxC	9_1	9	7	47	250	0	chasing
CxC	9_1	9	7	47	255	0	chasing
CxC	9_1	9	7	47	260	0	chasing
CxC	9_1	9	7	47	265	0	chasing
CxC	9_1	9	7	47	270	0	chasing
CxC	9_1	9	7	47	275	0	chasing
CxC	9_1	9	7	47	280	0	chasing
CxC	9_1	9	7	47	285	0	chasing
CxC	9_1	9	7	47	290	0	chasing
CxC	9_1	9	7	47	295	0	chasing
CxC	9_1	9	7	47	300	0	chasing
CxC	10_1	8	8	48	5	0	chasing
CxC	10_1	8	8	48	10	0	chasing
CxC	10_1	8	8	48	15	0	chasing
CxC	10_1	8	8	48	20	1	chasing
CxC	10_1	8	8	48	25	0	chasing
CxC	10_1	8	8	48	30	0	chasing
CxC	10_1	8	8	48	35	0	chasing
CxC	10_1	8	8	48	40	0	chasing
CxC	10_1	8	8	48	45	0	chasing
CxC	10_1	8	8	48	50	0	chasing
CxC	10_1	8	8	48	55	0	chasing
CxC	10_1	8	8	48	60	0	chasing
CxC	10_1	8	8	48	65	0	chasing
CxC	10_1	8	8	48	70	0	chasing
CxC	10_1	8	8	48	75	0	chasing
CxC	10_1	8	8	48	80	0	chasing
CxC	10_1	8	8	48	85	0	chasing
CxC	10_1	8	8	48	90	0	chasing
CxC	10_1	8	8	48	95	0	chasing
CxC	10_1	8	8	48	100	0	chasing
CxC	10_1	8	8	48	105	0	chasing
CxC	10_1	8	8	48	110	0	chasing
CxC	10_1	8	8	48	115	0	chasing
CxC	10_1	8	8	48	120	0	chasing
CxC	10_1	8	8	48	125	0	chasing
CxC	10_1	8	8	48	130	0	chasing
CxC	10_1	8	8	48	135	0	chasing
CxC	10_1	8	8	48	140	0	chasing
CxC	10_1	8	8	48	145	0	chasing
CxC	10_1	8	8	48	150	0	chasing
CxC	10_1	8	8	48	155	0	chasing
CxC	10_1	8	8	48	160	0	chasing
CxC	10_1	8	8	48	165	0	chasing
CxC	10_1	8	8	48	170	0	chasing
CxC	10_1	8	8	48	175	0	chasing

CxC	10_1	8	8	48	180	0	chasing
CxC	10_1	8	8	48	185	0	chasing
CxC	10_1	8	8	48	190	0	chasing
CxC	10_1	8	8	48	195	0	chasing
CxC	10_1	8	8	48	200	0	chasing
CxC	10_1	8	8	48	205	0	chasing
CxC	10_1	8	8	48	210	0	chasing
CxC	10_1	8	8	48	215	0	chasing
CxC	10_1	8	8	48	220	0	chasing
CxC	10_1	8	8	48	225	0	chasing
CxC	10_1	8	8	48	230	0	chasing
CxC	10_1	8	8	48	235	0	chasing
CxC	10_1	8	8	48	240	0	chasing
CxC	10_1	8	8	48	245	0	chasing
CxC	10_1	8	8	48	250	0	chasing
CxC	10_1	8	8	48	255	0	chasing
CxC	10_1	8	8	48	260	0	chasing
CxC	10_1	8	8	48	265	0	chasing
CxC	10_1	8	8	48	270	0	chasing
CxC	10_1	8	8	48	275	0	chasing
CxC	10_1	8	8	48	280	0	chasing
CxC	10_1	8	8	48	285	0	chasing
CxC	10_1	8	8	48	290	0	chasing
CxC	10_1	8	8	48	295	0	chasing
CxC	10_1	8	8	48	300	0	chasing
CxC	11_1	6	6	49	5	0	chasing
CxC	11_1	6	6	49	10	0	chasing
CxC	11_1	6	6	49	15	0	chasing
CxC	11_1	6	6	49	20	0	chasing
CxC	11_1	6	6	49	25	0	chasing
CxC	11_1	6	6	49	30	0	chasing
CxC	11_1	6	6	49	35	0	chasing
CxC	11_1	6	6	49	40	0	chasing
CxC	11_1	6	6	49	45	0	chasing
CxC	11_1	6	6	49	50	0	chasing
CxC	11_1	6	6	49	55	0	chasing
CxC	11_1	6	6	49	60	0	chasing
CxC	11_1	6	6	49	65	0	chasing
CxC	11_1	6	6	49	70	0	chasing
CxC	11_1	6	6	49	75	0	chasing
CxC	11_1	6	6	49	80	0	chasing
CxC	11_1	6	6	49	85	0	chasing
CxC	11_1	6	6	49	90	0	chasing
CxC	11_1	6	6	49	95	0	chasing
CxC	11_1	6	6	49	100	0	chasing
CxC	11_1	6	6	49	105	0	chasing
CxC	11_1	6	6	49	110	0	chasing
CxC	11_1	6	6	49	115	0	chasing

CxC	11_1	6	6	49	120	0	chasing
CxC	11_1	6	6	49	125	0	chasing
CxC	11_1	6	6	49	130	0	chasing
CxC	11_1	6	6	49	135	0	chasing
CxC	11_1	6	6	49	140	0	chasing
CxC	11_1	6	6	49	145	0	chasing
CxC	11_1	6	6	49	150	0	chasing
CxC	11_1	6	6	49	155	0	chasing
CxC	11_1	6	6	49	160	0	chasing
CxC	11_1	6	6	49	165	0	chasing
CxC	11_1	6	6	49	170	0	chasing
CxC	11_1	6	6	49	175	0	chasing
CxC	11_1	6	6	49	180	0	chasing
CxC	11_1	6	6	49	185	0	chasing
CxC	11_1	6	6	49	190	0	chasing
CxC	11_1	6	6	49	195	0	chasing
CxC	11_1	6	6	49	200	0	chasing
CxC	11_1	6	6	49	205	0	chasing
CxC	11_1	6	6	49	210	0	chasing
CxC	11_1	6	6	49	215	0	chasing
CxC	11_1	6	6	49	220	0	chasing
CxC	11_1	6	6	49	225	0	chasing
CxC	11_1	6	6	49	230	0	chasing
CxC	11_1	6	6	49	235	0	chasing
CxC	11_1	6	6	49	240	0	chasing
CxC	11_1	6	6	49	245	0	chasing
CxC	11_1	6	6	49	250	0	chasing
CxC	11_1	6	6	49	255	0	chasing
CxC	11_1	6	6	49	260	0	chasing
CxC	11_1	6	6	49	265	0	chasing
CxC	11_1	6	6	49	270	0	chasing
CxC	11_1	6	6	49	275	0	chasing
CxC	11_1	6	6	49	280	0	chasing
CxC	11_1	6	6	49	285	0	chasing
CxC	11_1	6	6	49	290	0	chasing
CxC	11_1	6	6	49	295	0	chasing
CxC	11_1	6	6	49	300	0	chasing
CxC	12_1	7	7	50	5	0	chasing
CxC	12_1	7	7	50	10	0	chasing
CxC	12_1	7	7	50	15	0	chasing
CxC	12_1	7	7	50	20	0	chasing
CxC	12_1	7	7	50	25	0	chasing
CxC	12_1	7	7	50	30	0	chasing
CxC	12_1	7	7	50	35	1	chasing
CxC	12_1	7	7	50	40	0	chasing
CxC	12_1	7	7	50	45	0	chasing
CxC	12_1	7	7	50	50	0	chasing
CxC	12_1	7	7	50	55	0	chasing

CxC	12_1	7	7	50	60	0	chasing
CxC	12_1	7	7	50	65	0	chasing
CxC	12_1	7	7	50	70	0	chasing
CxC	12_1	7	7	50	75	0	chasing
CxC	12_1	7	7	50	80	0	chasing
CxC	12_1	7	7	50	85	0	chasing
CxC	12_1	7	7	50	90	0	chasing
CxC	12_1	7	7	50	95	0	chasing
CxC	12_1	7	7	50	100	0	chasing
CxC	12_1	7	7	50	105	0	chasing
CxC	12_1	7	7	50	110	0	chasing
CxC	12_1	7	7	50	115	0	chasing
CxC	12_1	7	7	50	120	1	chasing
CxC	12_1	7	7	50	125	0	chasing
CxC	12_1	7	7	50	130	0	chasing
CxC	12_1	7	7	50	135	0	chasing
CxC	12_1	7	7	50	140	0	chasing
CxC	12_1	7	7	50	145	0	chasing
CxC	12_1	7	7	50	150	0	chasing
CxC	12_1	7	7	50	155	0	chasing
CxC	12_1	7	7	50	160	0	chasing
CxC	12_1	7	7	50	165	0	chasing
CxC	12_1	7	7	50	170	0	chasing
CxC	12_1	7	7	50	175	0	chasing
CxC	12_1	7	7	50	180	0	chasing
CxC	12_1	7	7	50	185	0	chasing
CxC	12_1	7	7	50	190	0	chasing
CxC	12_1	7	7	50	195	0	chasing
CxC	12_1	7	7	50	200	0	chasing
CxC	12_1	7	7	50	205	0	chasing
CxC	12_1	7	7	50	210	0	chasing
CxC	12_1	7	7	50	215	0	chasing
CxC	12_1	7	7	50	220	0	chasing
CxC	12_1	7	7	50	225	0	chasing
CxC	12_1	7	7	50	230	0	chasing
CxC	12_1	7	7	50	235	0	chasing
CxC	12_1	7	7	50	240	0	chasing
CxC	12_1	7	7	50	245	0	chasing
CxC	12_1	7	7	50	250	0	chasing
CxC	12_1	7	7	50	255	0	chasing
CxC	12_1	7	7	50	260	0	chasing
CxC	12_1	7	7	50	265	0	chasing
CxC	12_1	7	7	50	270	0	chasing
CxC	12_1	7	7	50	275	0	chasing
CxC	12_1	7	7	50	280	0	chasing
CxC	12_1	7	7	50	285	0	chasing
CxC	12_1	7	7	50	290	0	chasing
CxC	12_1	7	7	50	295	0	chasing

CxC	12_1	7	7	50	300	0	chasing
CxC	13_1	11	16	51	5	1	chasing
CxC	13_1	11	16	51	10	0	chasing
CxC	13_1	11	16	51	15	0	chasing
CxC	13_1	11	16	51	20	0	chasing
CxC	13_1	11	16	51	25	0	chasing
CxC	13_1	11	16	51	30	0	chasing
CxC	13_1	11	16	51	35	0	chasing
CxC	13_1	11	16	51	40	0	chasing
CxC	13_1	11	16	51	45	0	chasing
CxC	13_1	11	16	51	50	0	chasing
CxC	13_1	11	16	51	55	0	chasing
CxC	13_1	11	16	51	60	0	chasing
CxC	13_1	11	16	51	65	0	chasing
CxC	13_1	11	16	51	70	0	chasing
CxC	13_1	11	16	51	75	0	chasing
CxC	13_1	11	16	51	80	0	chasing
CxC	13_1	11	16	51	85	0	chasing
CxC	13_1	11	16	51	90	0	chasing
CxC	13_1	11	16	51	95	0	chasing
CxC	13_1	11	16	51	100	0	chasing
CxC	13_1	11	16	51	105	0	chasing
CxC	13_1	11	16	51	110	0	chasing
CxC	13_1	11	16	51	115	0	chasing
CxC	13_1	11	16	51	120	0	chasing
CxC	13_1	11	16	51	125	0	chasing
CxC	13_1	11	16	51	130	0	chasing
CxC	13_1	11	16	51	135	0	chasing
CxC	13_1	11	16	51	140	0	chasing
CxC	13_1	11	16	51	145	0	chasing
CxC	13_1	11	16	51	150	0	chasing
CxC	13_1	11	16	51	155	0	chasing
CxC	13_1	11	16	51	160	0	chasing
CxC	13_1	11	16	51	165	0	chasing
CxC	13_1	11	16	51	170	0	chasing
CxC	13_1	11	16	51	175	0	chasing
CxC	13_1	11	16	51	180	0	chasing
CxC	13_1	11	16	51	185	0	chasing
CxC	13_1	11	16	51	190	0	chasing
CxC	13_1	11	16	51	195	0	chasing
CxC	13_1	11	16	51	200	0	chasing
CxC	13_1	11	16	51	205	0	chasing
CxC	13_1	11	16	51	210	0	chasing
CxC	13_1	11	16	51	215	0	chasing
CxC	13_1	11	16	51	220	0	chasing
CxC	13_1	11	16	51	225	0	chasing
CxC	13_1	11	16	51	230	0	chasing
CxC	13_1	11	16	51	235	0	chasing

CxC	13_1	11	16	51	240	0	chasing
CxC	13_1	11	16	51	245	0	chasing
CxC	13_1	11	16	51	250	0	chasing
CxC	13_1	11	16	51	255	0	chasing
CxC	13_1	11	16	51	260	0	chasing
CxC	13_1	11	16	51	265	0	chasing
CxC	13_1	11	16	51	270	0	chasing
CxC	13_1	11	16	51	275	0	chasing
CxC	13_1	11	16	51	280	0	chasing
CxC	13_1	11	16	51	285	0	chasing
CxC	13_1	11	16	51	290	0	chasing
CxC	13_1	11	16	51	295	0	chasing
CxC	13_1	11	16	51	300	0	chasing
CxC	14_1	11	15	52	5	0	chasing
CxC	14_1	11	15	52	10	0	chasing
CxC	14_1	11	15	52	15	0	chasing
CxC	14_1	11	15	52	20	0	chasing
CxC	14_1	11	15	52	25	0	chasing
CxC	14_1	11	15	52	30	0	chasing
CxC	14_1	11	15	52	35	0	chasing
CxC	14_1	11	15	52	40	1	chasing
CxC	14_1	11	15	52	45	0	chasing
CxC	14_1	11	15	52	50	0	chasing
CxC	14_1	11	15	52	55	0	chasing
CxC	14_1	11	15	52	60	0	chasing
CxC	14_1	11	15	52	65	0	chasing
CxC	14_1	11	15	52	70	0	chasing
CxC	14_1	11	15	52	75	0	chasing
CxC	14_1	11	15	52	80	0	chasing
CxC	14_1	11	15	52	85	0	chasing
CxC	14_1	11	15	52	90	1	chasing
CxC	14_1	11	15	52	95	1	chasing
CxC	14_1	11	15	52	100	0	chasing
CxC	14_1	11	15	52	105	0	chasing
CxC	14_1	11	15	52	110	0	chasing
CxC	14_1	11	15	52	115	1	chasing
CxC	14_1	11	15	52	120	0	chasing
CxC	14_1	11	15	52	125	1	chasing
CxC	14_1	11	15	52	130	0	chasing
CxC	14_1	11	15	52	135	0	chasing
CxC	14_1	11	15	52	140	0	chasing
CxC	14_1	11	15	52	145	1	chasing
CxC	14_1	11	15	52	150	0	chasing
CxC	14_1	11	15	52	155	0	chasing
CxC	14_1	11	15	52	160	0	chasing
CxC	14_1	11	15	52	165	1	chasing
CxC	14_1	11	15	52	170	0	chasing
CxC	14_1	11	15	52	175	0	chasing

CxC	14_1	11	15	52	180	0	chasing
CxC	14_1	11	15	52	185	0	chasing
CxC	14_1	11	15	52	190	0	chasing
CxC	14_1	11	15	52	195	0	chasing
CxC	14_1	11	15	52	200	0	chasing
CxC	14_1	11	15	52	205	0	chasing
CxC	14_1	11	15	52	210	0	chasing
CxC	14_1	11	15	52	215	0	chasing
CxC	14_1	11	15	52	220	0	chasing
CxC	14_1	11	15	52	225	0	chasing
CxC	14_1	11	15	52	230	0	chasing
CxC	14_1	11	15	52	235	0	chasing
CxC	14_1	11	15	52	240	0	chasing
CxC	14_1	11	15	52	245	0	chasing
CxC	14_1	11	15	52	250	0	chasing
CxC	14_1	11	15	52	255	0	chasing
CxC	14_1	11	15	52	260	0	chasing
CxC	14_1	11	15	52	265	0	chasing
CxC	14_1	11	15	52	270	0	chasing
CxC	14_1	11	15	52	275	0	chasing
CxC	14_1	11	15	52	280	0	chasing
CxC	14_1	11	15	52	285	0	chasing
CxC	14_1	11	15	52	290	0	chasing
CxC	14_1	11	15	52	295	0	chasing
CxC	14_1	11	15	52	300	0	chasing
CxC	15_1	9	13	53	5	0	chasing
CxC	15_1	9	13	53	10	0	chasing
CxC	15_1	9	13	53	15	0	chasing
CxC	15_1	9	13	53	20	0	chasing
CxC	15_1	9	13	53	25	0	chasing
CxC	15_1	9	13	53	30	0	chasing
CxC	15_1	9	13	53	35	0	chasing
CxC	15_1	9	13	53	40	0	chasing
CxC	15_1	9	13	53	45	0	chasing
CxC	15_1	9	13	53	50	0	chasing
CxC	15_1	9	13	53	55	0	chasing
CxC	15_1	9	13	53	60	0	chasing
CxC	15_1	9	13	53	65	0	chasing
CxC	15_1	9	13	53	70	0	chasing
CxC	15_1	9	13	53	75	0	chasing
CxC	15_1	9	13	53	80	0	chasing
CxC	15_1	9	13	53	85	0	chasing
CxC	15_1	9	13	53	90	0	chasing
CxC	15_1	9	13	53	95	0	chasing
CxC	15_1	9	13	53	100	0	chasing
CxC	15_1	9	13	53	105	0	chasing
CxC	15_1	9	13	53	110	0	chasing
CxC	15_1	9	13	53	115	0	chasing

CxC	15_1	9	13	53	120	0	chasing
CxC	15_1	9	13	53	125	0	chasing
CxC	15_1	9	13	53	130	0	chasing
CxC	15_1	9	13	53	135	0	chasing
CxC	15_1	9	13	53	140	0	chasing
CxC	15_1	9	13	53	145	0	chasing
CxC	15_1	9	13	53	150	0	chasing
CxC	15_1	9	13	53	155	0	chasing
CxC	15_1	9	13	53	160	0	chasing
CxC	15_1	9	13	53	165	0	chasing
CxC	15_1	9	13	53	170	0	chasing
CxC	15_1	9	13	53	175	0	chasing
CxC	15_1	9	13	53	180	0	chasing
CxC	15_1	9	13	53	185	0	chasing
CxC	15_1	9	13	53	190	0	chasing
CxC	15_1	9	13	53	195	0	chasing
CxC	15_1	9	13	53	200	0	chasing
CxC	15_1	9	13	53	205	0	chasing
CxC	15_1	9	13	53	210	0	chasing
CxC	15_1	9	13	53	215	0	chasing
CxC	15_1	9	13	53	220	0	chasing
CxC	15_1	9	13	53	225	0	chasing
CxC	15_1	9	13	53	230	0	chasing
CxC	15_1	9	13	53	235	0	chasing
CxC	15_1	9	13	53	240	0	chasing
CxC	15_1	9	13	53	245	0	chasing
CxC	15_1	9	13	53	250	0	chasing
CxC	15_1	9	13	53	255	0	chasing
CxC	15_1	9	13	53	260	0	chasing
CxC	15_1	9	13	53	265	0	chasing
CxC	15_1	9	13	53	270	0	chasing
CxC	15_1	9	13	53	275	0	chasing
CxC	15_1	9	13	53	280	1	chasing
CxC	15_1	9	13	53	285	0	chasing
CxC	15_1	9	13	53	290	0	chasing
CxC	15_1	9	13	53	295	0	chasing
CxC	15_1	9	13	53	300	0	chasing
CxC	16_1	8	11	54	5	0	chasing
CxC	16_1	8	11	54	10	0	chasing
CxC	16_1	8	11	54	15	0	chasing
CxC	16_1	8	11	54	20	0	chasing
CxC	16_1	8	11	54	25	0	chasing
CxC	16_1	8	11	54	30	0	chasing
CxC	16_1	8	11	54	35	0	chasing
CxC	16_1	8	11	54	40	0	chasing
CxC	16_1	8	11	54	45	0	chasing
CxC	16_1	8	11	54	50	0	chasing
CxC	16_1	8	11	54	55	0	chasing

CxC	16_1	8	11	54	60	0	chasing
CxC	16_1	8	11	54	65	0	chasing
CxC	16_1	8	11	54	70	0	chasing
CxC	16_1	8	11	54	75	0	chasing
CxC	16_1	8	11	54	80	0	chasing
CxC	16_1	8	11	54	85	0	chasing
CxC	16_1	8	11	54	90	0	chasing
CxC	16_1	8	11	54	95	0	chasing
CxC	16_1	8	11	54	100	0	chasing
CxC	16_1	8	11	54	105	0	chasing
CxC	16_1	8	11	54	110	0	chasing
CxC	16_1	8	11	54	115	1	chasing
CxC	16_1	8	11	54	120	0	chasing
CxC	16_1	8	11	54	125	0	chasing
CxC	16_1	8	11	54	130	0	chasing
CxC	16_1	8	11	54	135	0	chasing
CxC	16_1	8	11	54	140	0	chasing
CxC	16_1	8	11	54	145	0	chasing
CxC	16_1	8	11	54	150	0	chasing
CxC	16_1	8	11	54	155	0	chasing
CxC	16_1	8	11	54	160	0	chasing
CxC	16_1	8	11	54	165	0	chasing
CxC	16_1	8	11	54	170	0	chasing
CxC	16_1	8	11	54	175	0	chasing
CxC	16_1	8	11	54	180	0	chasing
CxC	16_1	8	11	54	185	0	chasing
CxC	16_1	8	11	54	190	0	chasing
CxC	16_1	8	11	54	195	0	chasing
CxC	16_1	8	11	54	200	0	chasing
CxC	16_1	8	11	54	205	0	chasing
CxC	16_1	8	11	54	210	0	chasing
CxC	16_1	8	11	54	215	0	chasing
CxC	16_1	8	11	54	220	0	chasing
CxC	16_1	8	11	54	225	0	chasing
CxC	16_1	8	11	54	230	0	chasing
CxC	16_1	8	11	54	235	0	chasing
CxC	16_1	8	11	54	240	0	chasing
CxC	16_1	8	11	54	245	0	chasing
CxC	16_1	8	11	54	250	0	chasing
CxC	16_1	8	11	54	255	0	chasing
CxC	16_1	8	11	54	260	0	chasing
CxC	16_1	8	11	54	265	0	chasing
CxC	16_1	8	11	54	270	0	chasing
CxC	16_1	8	11	54	275	0	chasing
CxC	16_1	8	11	54	280	0	chasing
CxC	16_1	8	11	54	285	0	chasing
CxC	16_1	8	11	54	290	0	chasing
CxC	16_1	8	11	54	295	0	chasing

CxC	16_1	8	11	54	300	0	chasing
CxC	17_1	12	10	55	5	0	chasing
CxC	17_1	12	10	55	10	0	chasing
CxC	17_1	12	10	55	15	0	chasing
CxC	17_1	12	10	55	20	0	chasing
CxC	17_1	12	10	55	25	0	chasing
CxC	17_1	12	10	55	30	0	chasing
CxC	17_1	12	10	55	35	0	chasing
CxC	17_1	12	10	55	40	0	chasing
CxC	17_1	12	10	55	45	0	chasing
CxC	17_1	12	10	55	50	0	chasing
CxC	17_1	12	10	55	55	0	chasing
CxC	17_1	12	10	55	60	0	chasing
CxC	17_1	12	10	55	65	0	chasing
CxC	17_1	12	10	55	70	0	chasing
CxC	17_1	12	10	55	75	0	chasing
CxC	17_1	12	10	55	80	0	chasing
CxC	17_1	12	10	55	85	0	chasing
CxC	17_1	12	10	55	90	0	chasing
CxC	17_1	12	10	55	95	0	chasing
CxC	17_1	12	10	55	100	0	chasing
CxC	17_1	12	10	55	105	0	chasing
CxC	17_1	12	10	55	110	0	chasing
CxC	17_1	12	10	55	115	0	chasing
CxC	17_1	12	10	55	120	0	chasing
CxC	17_1	12	10	55	125	0	chasing
CxC	17_1	12	10	55	130	0	chasing
CxC	17_1	12	10	55	135	0	chasing
CxC	17_1	12	10	55	140	0	chasing
CxC	17_1	12	10	55	145	0	chasing
CxC	17_1	12	10	55	150	0	chasing
CxC	17_1	12	10	55	155	0	chasing
CxC	17_1	12	10	55	160	0	chasing
CxC	17_1	12	10	55	165	0	chasing
CxC	17_1	12	10	55	170	0	chasing
CxC	17_1	12	10	55	175	0	chasing
CxC	17_1	12	10	55	180	0	chasing
CxC	17_1	12	10	55	185	0	chasing
CxC	17_1	12	10	55	190	0	chasing
CxC	17_1	12	10	55	195	0	chasing
CxC	17_1	12	10	55	200	0	chasing
CxC	17_1	12	10	55	205	0	chasing
CxC	17_1	12	10	55	210	0	chasing
CxC	17_1	12	10	55	215	0	chasing
CxC	17_1	12	10	55	220	0	chasing
CxC	17_1	12	10	55	225	0	chasing
CxC	17_1	12	10	55	230	0	chasing
CxC	17_1	12	10	55	235	0	chasing

CxC	17_1	12	10	55	240	0	chasing
CxC	17_1	12	10	55	245	0	chasing
CxC	17_1	12	10	55	250	0	chasing
CxC	17_1	12	10	55	255	0	chasing
CxC	17_1	12	10	55	260	0	chasing
CxC	17_1	12	10	55	265	0	chasing
CxC	17_1	12	10	55	270	0	chasing
CxC	17_1	12	10	55	275	0	chasing
CxC	17_1	12	10	55	280	0	chasing
CxC	17_1	12	10	55	285	0	chasing
CxC	17_1	12	10	55	290	0	chasing
CxC	17_1	12	10	55	295	0	chasing
CxC	17_1	12	10	55	300	0	chasing
CxC	18_1	7	9	56	5	0	chasing
CxC	18_1	7	9	56	10	0	chasing
CxC	18_1	7	9	56	15	0	chasing
CxC	18_1	7	9	56	20	0	chasing
CxC	18_1	7	9	56	25	0	chasing
CxC	18_1	7	9	56	30	0	chasing
CxC	18_1	7	9	56	35	0	chasing
CxC	18_1	7	9	56	40	0	chasing
CxC	18_1	7	9	56	45	0	chasing
CxC	18_1	7	9	56	50	0	chasing
CxC	18_1	7	9	56	55	0	chasing
CxC	18_1	7	9	56	60	0	chasing
CxC	18_1	7	9	56	65	0	chasing
CxC	18_1	7	9	56	70	0	chasing
CxC	18_1	7	9	56	75	0	chasing
CxC	18_1	7	9	56	80	0	chasing
CxC	18_1	7	9	56	85	0	chasing
CxC	18_1	7	9	56	90	0	chasing
CxC	18_1	7	9	56	95	0	chasing
CxC	18_1	7	9	56	100	0	chasing
CxC	18_1	7	9	56	105	0	chasing
CxC	18_1	7	9	56	110	0	chasing
CxC	18_1	7	9	56	115	0	chasing
CxC	18_1	7	9	56	120	0	chasing
CxC	18_1	7	9	56	125	0	chasing
CxC	18_1	7	9	56	130	0	chasing
CxC	18_1	7	9	56	135	0	chasing
CxC	18_1	7	9	56	140	0	chasing
CxC	18_1	7	9	56	145	0	chasing
CxC	18_1	7	9	56	150	0	chasing
CxC	18_1	7	9	56	155	0	chasing
CxC	18_1	7	9	56	160	0	chasing
CxC	18_1	7	9	56	165	0	chasing
CxC	18_1	7	9	56	170	0	chasing
CxC	18_1	7	9	56	175	0	chasing

CxC	18_1	7	9	56	180	0	chasing
CxC	18_1	7	9	56	185	0	chasing
CxC	18_1	7	9	56	190	0	chasing
CxC	18_1	7	9	56	195	0	chasing
CxC	18_1	7	9	56	200	0	chasing
CxC	18_1	7	9	56	205	0	chasing
CxC	18_1	7	9	56	210	0	chasing
CxC	18_1	7	9	56	215	0	chasing
CxC	18_1	7	9	56	220	0	chasing
CxC	18_1	7	9	56	225	0	chasing
CxC	18_1	7	9	56	230	0	chasing
CxC	18_1	7	9	56	235	0	chasing
CxC	18_1	7	9	56	240	0	chasing
CxC	18_1	7	9	56	245	0	chasing
CxC	18_1	7	9	56	250	0	chasing
CxC	18_1	7	9	56	255	0	chasing
CxC	18_1	7	9	56	260	0	chasing
CxC	18_1	7	9	56	265	0	chasing
CxC	18_1	7	9	56	270	0	chasing
CxC	18_1	7	9	56	275	0	chasing
CxC	18_1	7	9	56	280	0	chasing
CxC	18_1	7	9	56	285	0	chasing
CxC	18_1	7	9	56	290	0	chasing
CxC	18_1	7	9	56	295	0	chasing
CxC	18_1	7	9	56	300	0	chasing
CxC	19_1	11	8	57	5	0	chasing
CxC	19_1	11	8	57	10	0	chasing
CxC	19_1	11	8	57	15	0	chasing
CxC	19_1	11	8	57	20	0	chasing
CxC	19_1	11	8	57	25	0	chasing
CxC	19_1	11	8	57	30	0	chasing
CxC	19_1	11	8	57	35	0	chasing
CxC	19_1	11	8	57	40	0	chasing
CxC	19_1	11	8	57	45	0	chasing
CxC	19_1	11	8	57	50	0	chasing
CxC	19_1	11	8	57	55	0	chasing
CxC	19_1	11	8	57	60	0	chasing
CxC	19_1	11	8	57	65	0	chasing
CxC	19_1	11	8	57	70	0	chasing
CxC	19_1	11	8	57	75	0	chasing
CxC	19_1	11	8	57	80	0	chasing
CxC	19_1	11	8	57	85	0	chasing
CxC	19_1	11	8	57	90	0	chasing
CxC	19_1	11	8	57	95	0	chasing
CxC	19_1	11	8	57	100	0	chasing
CxC	19_1	11	8	57	105	0	chasing
CxC	19_1	11	8	57	110	0	chasing
CxC	19_1	11	8	57	115	0	chasing

CxC	19_1	11	8	57	120	0	chasing
CxC	19_1	11	8	57	125	0	chasing
CxC	19_1	11	8	57	130	0	chasing
CxC	19_1	11	8	57	135	0	chasing
CxC	19_1	11	8	57	140	0	chasing
CxC	19_1	11	8	57	145	0	chasing
CxC	19_1	11	8	57	150	0	chasing
CxC	19_1	11	8	57	155	0	chasing
CxC	19_1	11	8	57	160	0	chasing
CxC	19_1	11	8	57	165	0	chasing
CxC	19_1	11	8	57	170	0	chasing
CxC	19_1	11	8	57	175	0	chasing
CxC	19_1	11	8	57	180	0	chasing
CxC	19_1	11	8	57	185	0	chasing
CxC	19_1	11	8	57	190	0	chasing
CxC	19_1	11	8	57	195	0	chasing
CxC	19_1	11	8	57	200	0	chasing
CxC	19_1	11	8	57	205	0	chasing
CxC	19_1	11	8	57	210	0	chasing
CxC	19_1	11	8	57	215	0	chasing
CxC	19_1	11	8	57	220	0	chasing
CxC	19_1	11	8	57	225	0	chasing
CxC	19_1	11	8	57	230	0	chasing
CxC	19_1	11	8	57	235	0	chasing
CxC	19_1	11	8	57	240	0	chasing
CxC	19_1	11	8	57	245	0	chasing
CxC	19_1	11	8	57	250	0	chasing
CxC	19_1	11	8	57	255	0	chasing
CxC	19_1	11	8	57	260	0	chasing
CxC	19_1	11	8	57	265	0	chasing
CxC	19_1	11	8	57	270	0	chasing
CxC	19_1	11	8	57	275	0	chasing
CxC	19_1	11	8	57	280	0	chasing
CxC	19_1	11	8	57	285	0	chasing
CxC	19_1	11	8	57	290	0	chasing
CxC	19_1	11	8	57	295	0	chasing
CxC	19_1	11	8	57	300	0	chasing
CxC	20_1	13	7	58	5	0	chasing
CxC	20_1	13	7	58	10	0	chasing
CxC	20_1	13	7	58	15	0	chasing
CxC	20_1	13	7	58	20	0	chasing
CxC	20_1	13	7	58	25	0	chasing
CxC	20_1	13	7	58	30	0	chasing
CxC	20_1	13	7	58	35	0	chasing
CxC	20_1	13	7	58	40	0	chasing
CxC	20_1	13	7	58	45	0	chasing
CxC	20_1	13	7	58	50	0	chasing
CxC	20_1	13	7	58	55	0	chasing

CxC	20_1	13	7	58	60	0	chasing
CxC	20_1	13	7	58	65	0	chasing
CxC	20_1	13	7	58	70	0	chasing
CxC	20_1	13	7	58	75	0	chasing
CxC	20_1	13	7	58	80	0	chasing
CxC	20_1	13	7	58	85	0	chasing
CxC	20_1	13	7	58	90	0	chasing
CxC	20_1	13	7	58	95	0	chasing
CxC	20_1	13	7	58	100	0	chasing
CxC	20_1	13	7	58	105	0	chasing
CxC	20_1	13	7	58	110	0	chasing
CxC	20_1	13	7	58	115	0	chasing
CxC	20_1	13	7	58	120	0	chasing
CxC	20_1	13	7	58	125	0	chasing
CxC	20_1	13	7	58	130	0	chasing
CxC	20_1	13	7	58	135	0	chasing
CxC	20_1	13	7	58	140	0	chasing
CxC	20_1	13	7	58	145	0	chasing
CxC	20_1	13	7	58	150	0	chasing
CxC	20_1	13	7	58	155	0	chasing
CxC	20_1	13	7	58	160	0	chasing
CxC	20_1	13	7	58	165	0	chasing
CxC	20_1	13	7	58	170	0	chasing
CxC	20_1	13	7	58	175	0	chasing
CxC	20_1	13	7	58	180	0	chasing
CxC	20_1	13	7	58	185	0	chasing
CxC	20_1	13	7	58	190	0	chasing
CxC	20_1	13	7	58	195	0	chasing
CxC	20_1	13	7	58	200	0	chasing
CxC	20_1	13	7	58	205	0	chasing
CxC	20_1	13	7	58	210	0	chasing
CxC	20_1	13	7	58	215	0	chasing
CxC	20_1	13	7	58	220	0	chasing
CxC	20_1	13	7	58	225	0	chasing
CxC	20_1	13	7	58	230	0	chasing
CxC	20_1	13	7	58	235	0	chasing
CxC	20_1	13	7	58	240	0	chasing
CxC	20_1	13	7	58	245	0	chasing
CxC	20_1	13	7	58	250	0	chasing
CxC	20_1	13	7	58	255	0	chasing
CxC	20_1	13	7	58	260	0	chasing
CxC	20_1	13	7	58	265	0	chasing
CxC	20_1	13	7	58	270	0	chasing
CxC	20_1	13	7	58	275	0	chasing
CxC	20_1	13	7	58	280	0	chasing
CxC	20_1	13	7	58	285	0	chasing
CxC	20_1	13	7	58	290	0	chasing
CxC	20_1	13	7	58	295	0	chasing

CxC	20_1	13	7	58	300	0	chasing

weight_diff
2
2
2
2
2
2
2
2
2
2
2
2
2
2
2
2
2
2
2
2
2
2
2
2
2
2
2
2
2
2
2
2
2
2
2
2
2
2
2
2
2
2
2
2
2
2
2

	2
	2
	2
	2
	2
	2
	2
	2
	2
	2
	2
	2
	2
	2
	2
	2
	2
	2
	2
	2
	2
	2
	2
	2
	2
	2
	2
	2
	2
	2
	2
	2
	2
	2
	2
	2
	2
	2
	2
	2
	2
	2
	2
	2
	2
	2
	2
	2

2
2
2
2
2
2
2
2
2
2
2
2
2
2
2
2
2
2
2
2
2
2
2
2
2
5
5
5
5
5
5
5
5
5
5
5
5
5
5
5
5
5
5
5
5
5
5
5

5
5
5
5
5
5
5
5
5
5
5
5
5
5
5
5
5
5
5
5
5
5
5
5
5
5
5
5
5
5
5
5
5
5
5
5
5
1
1
1
1
1
1
1
1
1
1
1

1
1
1
1
1
1
1
1
1
1
1
1
1
1
1
1
1
1
1
1
1
1
1
1
1
1
1
1
1
1
1
1
1
1
1
1
1
1
1
1
1
1
1
1
1
1
1
1

1
1
1
1
1
1
1
1
1
1
1
1
1
1
1
1
1
1
1
1
1
1
1
1
1
1
1
1
1
1
1
1
1
1
1
1
1
1
1
1
1
1
1
1
1
1
1
1

1
1
1
1
1
1
1
1
1
1
1
1
1
2
2
2
2
2
2
2
2
2
2
2
2
2
2
2
2
2
2
2
2
2
2
2
2
2
2
2
2
2
2
2
2
2

2
2
2
2
2
2
2
2
2
2
2
2
2
2
2
2
2
2
2
2
2
2
2
2
2
2
2
2
2
2
2
2
2
2
2
2
2
2
2
2
2
2
2
2
2
2
2
2

2
2
2
2
2
2
2
2
2
2
2
2
2
2
2
2
2
2
2
2
2
2
2
2
2
2
2
2
2
2
2
2
2
2
2
2
2
1
1
1
1
1
1
1
1
1
1
1

1
1
1
1
1
1
1
1
1
1
1
1
1
1
1
1
1
1
1
1
1
1
1
1
1
1
1
1
1
1
1
1
1
1
1
1
1
1
1
1
1
1
1
1
1
1
1
1

1
2
2
2
2
2
2
2
2
2
2
2
2
2
2
2
2
2
2
2
2
2
2
2
2
2
2
2
2
2
2
2
2
2
2
2
2
2
2
2
2
2
2
2
2
2
2
2

2
2
2
2
2
2
2
2
2
2
2
2
2
1
1
1
1
1
1
1
1
1
1
1
1
1
1
1
1
1
1
1
1
1
1
1
1
1
1
1
1
1
1
1
1
1
1
1

1
1
1
1
1
1
1
1
1
1
1
1
1
1
1
1
1
1
1
1
1
1
1
1
1
1
1
1
1
1
1
1
1
1
1
1
1
1
1
1
1
1
1
1
1
1
1
1

1
1
1
1
1
1
1
1
1
1
1
1
1
1
1
1
1
1
1
1
1
1
1
1
1
1
1
1
1
1
1
1
1
1
1
1
1
0
0
0
0
0
0
0
0
0
0
0

	0
	0
	0
	0
	0
	0
	0
	0
	0
	0
	0
	0
	0
	0
	0
	0
	0
	0
	0
	0
	0
	0
	0
	0
	0
	0
	0
	0
	0
	0
	0
	0
	0
	0
	0
	0
	0
	0
	0
	0
	0
	0
	0
	0
	0
	0
	0
	0

0
2
2
2
2
2
2
2
2
2
2
2
2
2
2
2
2
2
2
2
2
2
2
2
2
2
2
2
2
2
2
2
2
2
2
2
2
2
2
2
2
2
2
2
2
2
2
2

2
2
2
2
2
2
2
2
2
2
2
2
2
1
1
1
1
1
1
1
1
1
1
1
1
1
1
1
1
1
1
1
1
1
1
1
1
1
1
1
1
1
1
1
1
1

1
1
1
1
1
1
1
1
1
1
1
1
1
1
1
1
1
1
1
1
1
1
1
1
1
0
0
0
0
0
0
0
0
0
0
0
0
0
0
0
0
0
0
0
0
0
0
0

0
0
0
0
0
0
0
0
0
0
0
0
0
0
0
0
0
0
0
0
0
0
0
0
0
0
0
0
0
0
0
0
0
0
0
0
0
1
1
1
1
1
1
1
1
1
1
1

1
1
1
1
1
1
1
1
1
1
1
1
1
1
1
1
1
1
1
1
1
1
1
1
1
1
1
1
1
1
1
1
1
1
1
1
1
1
1
1
1
1
1
1
1
1
1
1

1
2
2
2
2
2
2
2
2
2
2
2
2
2
2
2
2
2
2
2
2
2
2
2
2
2
2
2
2
2
2
2
2
2
2
2
2
2
2
2
2
2
2
2
2
2
2
2

2
2
2
2
2
2
2
2
2
2
2
2
2
3
3
3
3
3
3
3
3
3
3
3
3
3
3
3
3
3
3
3
3
3
3
3
3
3
3
3
3
3
3
3
3
3

3
3
3
3
3
3
3
3
3
3
3
3
3
3
3
3
3
3
3
3
3
3
3
3
3
5
5
5
5
5
5
5
5
5
5
5
5
5
5
5
5
5
5
5
5
5
5
5

5
5
5
5
5
5
5
5
5
5
5
5
5
5
5
5
5
5
5
5
5
5
5
5
5
5
5
5
5
5
5
5
5
5
5
5
5
3
3
3
3
3
3
3
3
3
3
3

3
3
3
3
3
3
3
3
3
3
3
3
3
3
3
3
3
3
3
3
3
3
3
3
3
3
3
3
3
3
3
3
3
3
3
3
3
3
3
3
3
3
3
3
3
3
3
3

3
3
3
3
3
3
3
3
3
3
3
3
3
3
3
3
3
3
3
3
3
3
3
3
3
3
3
3
3
3
3
3
3
3
3
3
3
3
3
3
3
3
3
3
3
3
3
3

3
3
3
3
3
3
3
3
3
3
3
3
3
1
1
1
1
1
1
1
1
1
1
1
1
1
1
1
1
1
1
1
1
1
1
1
1
1
1
1
1
1
1
1
1
1
1

1
1
1
1
1
1
1
1
1
1
1
1
1
1
1
1
1
1
1
1
1
1
1
1
1
1
1
1
1
1
1
1
1
1
1
1
1
1
1
1
1
1
1
1
1
1
1
1

1
1
1
1
1
1
1
1
1
1
1
1
1
1
1
1
1
1
1
1
1
1
1
1
1
1
1
1
1
1
1
1
1
1
1
1
1
1
1
1
1
1
1
1
1
1
1
1

1
1
1
1
1
1
1
1
1
1
1
1
1
1
1
1
1
1
1
1
1
1
1
1
1
1
1
1
1
1
1
1
1
1
1
1
1
1
1
1
1
1
1
1
1
1
1
1

1
0
0
0
0
0
0
0
0
0
0
0
0
0
0
0
0
0
0
0
0
0
0
0
0
0
0
0
0
0
0
0
0
0
0
0
0
0
0
0
0
0
0
0
0
0
0
0

0
0
0
0
0
0
0
0
0
0
0
0
0
1
1
1
1
1
1
1
1
1
1
1
1
1
1
1
1
1
1
1
1
1
1
1
1
1
1
1
1
1
1
1
1
1

1
1
1
1
1
1
1
1
1
1
1
1
1
1
1
1
1
1
1
1
1
1
1
1
1
1
1
1
1
1
1
1
1
1
1
1
1
1
1
1
1
1
1
1
1
1
1
1

1
1
1
1
1
1
1
1
1
1
1
1
1
1
1
1
1
1
1
1
1
1
1
1
1
1
1
1
1
1
1
1
1
1
1
1
1
1
1
1
1
1
1
1
1
1
1
1

1
1
1
1
1
1
1
1
1
1
1
1
1
1
1
1
1
1
1
1
1
1
1
1
1
1
1
1
1
1
1
1
1
1
1
1
1
1
1
1
1
1
1
1
1
1
1
1

1
2
2
2
2
2
2
2
2
2
2
2
2
2
2
2
2
2
2
2
2
2
2
2
2
2
2
2
2
2
2
2
2
2
2
2
2
2
2
2
2
2
2
2
2
2
2
2

2
2
2
2
2
2
2
2
2
2
2
2
2
0
0
0
0
0
0
0
0
0
0
0
0
0
0
0
0
0
0
0
0
0
0
0
0
0
0
0
0
0
0
0
0
0

0
0
0
0
0
0
0
0
0
0
0
0
0
0
0
0
0
0
0
0
0
0
0
0
0
0
0
0
0
0
0
0
0
0
0
0
0
0
0
0
0
0
0
0
0
0
0
0

0
0
0
0
0
0
0
0
0
0
0
0
0
0
0
0
0
0
0
0
0
0
0
0
0
0
0
0
0
0
0
0
0
0
0
0
0
0
0
0
0
0
0
0
0
0
0
0

0
0
0
0
0
0
0
0
0
0
0
0
0
0
0
0
0
0
0
0
0
0
0
0
0
0
0
0
0
0
0
0
0
0
0
0
0
0
0
0
0
0
0
0
0
0
0
0

0
5
5
5
5
5
5
5
5
5
5
5
5
5
5
5
5
5
5
5
5
5
5
5
5
5
5
5
5
5
5
5
5
5
5
5
5
5
5
5
5
5
5
5
5
5
5
5

5
5
5
5
5
5
5
5
5
5
5
5
5
4
4
4
4
4
4
4
4
4
4
4
4
4
4
4
4
4
4
4
4
4
4
4
4
4
4
4
4
4
4
4
4
4

4
4
4
4
4
4
4
4
4
4
4
4
4
4
4
4
4
4
4
4
4
4
4
4
4
4
4
4
4
4
4
4
4
4
4
4
4
4
4
4
4
4
4
4
4
4
4
4

4
4
4
4
4
4
4
4
4
4
4
4
4
4
4
4
4
4
4
4
4
4
4
4
4
4
4
4
4
4
4
4
4
4
4
4
4
3
3
3
3
3
3
3
3
3
3
3

3
3
3
3
3
3
3
3
3
3
3
3
3
3
3
3
3
3
3
3
3
3
3
3
3
3
3
3
3
3
3
3
3
3
3
3
3
3
3
3
3
3
3
3
3
3
3
3

3
2
2
2
2
2
2
2
2
2
2
2
2
2
2
2
2
2
2
2
2
2
2
2
2
2
2
2
2
2
2
2
2
2
2
2
2
2
2
2
2
2
2
2
2
2
2
2

2
2
2
2
2
2
2
2
2
2
2
2
2
2
2
2
2
2
2
2
2
2
2
2
2
2
2
2
2
2
2
2
2
2
2
2
2
2
2
2
2
2
2
2
2
2
2
2

2
2
2
2
2
2
2
2
2
2
2
2
2
2
2
2
2
2
2
2
2
2
2
2
2
3
3
3
3
3
3
3
3
3
3
3
3
3
3
3
3
3
3
3
3
3
3
3

3
3
3
3
3
3
3
3
3
3
3
3
3
3
3
3
3
3
3
3
3
3
3
3
3
3
3
3
3
3
3
3
3
3
3
3
3
6
6
6
6
6
6
6
6
6
6
6

6
6
6
6
6
6
6
6
6
6
6
6
6
6
6
6
6
6
6
6
6
6
6
6
6
6
6
6
6
6
6
6
6
6
6
6
6
6
6
6
6
6
6
6
6
6
6
6

6
2
2
2
2
2
2
2
2
2
2
2
2
2
2
2
2
2
2
2
2
2
2
2
2
2
2
2
2
2
2
2
2
2
2
2
2
2
2
2
2
2
2
2
2
2
2
2

2
2
2
2
2
2
2
2
2
2
2
2
2
2
2
2
2
2
2
2
2
2
2
2
2
2
2
2
2
2
2
2
2
2
2
2
2
2
2
2
2
2
2
2
2
2
2
2

2
2
2
2
2
2
2
2
2
2
2
2
2
2
2
2
2
2
2
2
2
2
2
2
2
5
5
5
5
5
5
5
5
5
5
5
5
5
5
5
5
5
5
5
5
5
5
5

5
5
5
5
5
5
5
5
5
5
5
5
5
5
5
5
5
5
5
5
5
5
5
5
5
5
5
5
5
5
5
5
5
5
5
5
5
1
1
1
1
1
1
1
1
1
1
1

1
1
1
1
1
1
1
1
1
1
1
1
1
1
1
1
1
1
1
1
1
1
1
1
1
1
1
1
1
1
1
1
1
1
1
1
1
1
1
1
1
1
1
1
1
1
1
1

1
1
1
1
1
1
1
1
1
1
1
1
1
1
1
1
1
1
1
1
1
1
1
1
1
1
1
1
1
1
1
1
1
1
1
1
1
1
1
1
1
1
1
1
1
1
1
1

1
1
1
1
1
1
1
1
1
1
1
1
1
2
2
2
2
2
2
2
2
2
2
2
2
2
2
2
2
2
2
2
2
2
2
2
2
2
2
2
2
2
2
2
2
2
2
2

2
2
2
2
2
2
2
2
2
2
2
2
2
2
2
2
2
2
2
2
2
2
2
2
2
2
2
2
2
2
2
2
2
2
2
2
2
2
2
2
2
2
2
2
2
2
2
2

2
2
2
2
2
2
2
2
2
2
2
2
2
2
2
2
2
2
2
2
2
2
2
2
2
2
2
2
2
2
2
2
2
2
2
2
2
1
1
1
1
1
1
1
1
1
1
1

1
1
1
1
1
1
1
1
1
1
1
1
1
1
1
1
1
1
1
1
1
1
1
1
1
1
1
1
1
1
1
1
1
1
1
1
1
1
1
1
1
1
1
1
1
1
1
1

1
2
2
2
2
2
2
2
2
2
2
2
2
2
2
2
2
2
2
2
2
2
2
2
2
2
2
2
2
2
2
2
2
2
2
2
2
2
2
2
2
2
2
2
2
2
2
2

2
2
2
2
2
2
2
2
2
2
2
2
2
1
1
1
1
1
1
1
1
1
1
1
1
1
1
1
1
1
1
1
1
1
1
1
1
1
1
1
1
1
1
1
1
1

1
1
1
1
1
1
1
1
1
1
1
1
1
1
1
1
1
1
1
1
1
1
1
1
1
1
1
1
1
1
1
1
1
1
1
1
1
1
1
1
1
1
1
1
1
1
1
1

1
1
1
1
1
1
1
1
1
1
1
1
1
1
1
1
1
1
1
1
1
1
1
1
1
1
1
1
1
1
1
1
1
1
1
1
1
0
0
0
0
0
0
0
0
0
0
0

0
0
0
0
0
0
0
0
0
0
0
0
0
0
0
0
0
0
0
0
0
0
0
0
0
0
0
0
0
0
0
0
0
0
0
0
0
0
0
0
0
0
0
0
0
0
0
0

0
2
2
2
2
2
2
2
2
2
2
2
2
2
2
2
2
2
2
2
2
2
2
2
2
2
2
2
2
2
2
2
2
2
2
2
2
2
2
2
2
2
2
2
2
2
2
2

2
2
2
2
2
2
2
2
2
2
2
2
2
1
1
1
1
1
1
1
1
1
1
1
1
1
1
1
1
1
1
1
1
1
1
1
1
1
1
1
1
1
1
1
1
1
1
1

1
1
1
1
1
1
1
1
1
1
1
1
1
1
1
1
1
1
1
1
1
1
1
1
1
0
0
0
0
0
0
0
0
0
0
0
0
0
0
0
0
0
0
0
0
0
0
0

0
0
0
0
0
0
0
0
0
0
0
0
0
0
0
0
0
0
0
0
0
0
0
0
0
0
0
0
0
0
0
0
0
0
0
0
0
1
1
1
1
1
1
1
1
1
1
1

1
1
1
1
1
1
1
1
1
1
1
1
1
1
1
1
1
1
1
1
1
1
1
1
1
1
1
1
1
1
1
1
1
1
1
1
1
1
1
1
1
1
1
1
1
1
1
1

1
2
2
2
2
2
2
2
2
2
2
2
2
2
2
2
2
2
2
2
2
2
2
2
2
2
2
2
2
2
2
2
2
2
2
2
2
2
2
2
2
2
2
2
2
2
2
2

2
2
2
2
2
2
2
2
2
2
2
2
2
3
3
3
3
3
3
3
3
3
3
3
3
3
3
3
3
3
3
3
3
3
3
3
3
3
3
3
3
3
3
3
3
3
3
3

3
3
3
3
3
3
3
3
3
3
3
3
3
3
3
3
3
3
3
3
3
3
3
3
3
5
5
5
5
5
5
5
5
5
5
5
5
5
5
5
5
5
5
5
5
5
5
5

5
5
5
5
5
5
5
5
5
5
5
5
5
5
5
5
5
5
5
5
5
5
5
5
5
5
5
5
5
5
5
5
5
5
5
5
5
3
3
3
3
3
3
3
3
3
3
3

3
3
3
3
3
3
3
3
3
3
3
3
3
3
3
3
3
3
3
3
3
3
3
3
3
3
3
3
3
3
3
3
3
3
3
3
3
3
3
3
3
3
3
3
3
3
3
3

3
3
3
3
3
3
3
3
3
3
3
3
3
3
3
3
3
3
3
3
3
3
3
3
3
3
3
3
3
3
3
3
3
3
3
3
3
3
3
3
3
3
3
3
3
3
3
3

3
3
3
3
3
3
3
3
3
3
3
3
3
1
1
1
1
1
1
1
1
1
1
1
1
1
1
1
1
1
1
1
1
1
1
1
1
1
1
1
1
1
1
1
1
1

1
1
1
1
1
1
1
1
1
1
1
1
1
1
1
1
1
1
1
1
1
1
1
1
1
1
1
1
1
1
1
1
1
1
1
1
1
1
1
1
1
1
1
1
1
1
1
1

1
1
1
1
1
1
1
1
1
1
1
1
1
1
1
1
1
1
1
1
1
1
1
1
1
1
1
1
1
1
1
1
1
1
1
1
1
1
1
1
1
1
1
1
1
1
1
1

1
1
1
1
1
1
1
1
1
1
1
1
1
1
1
1
1
1
1
1
1
1
1
1
1
1
1
1
1
1
1
1
1
1
1
1
1
1
1
1
1
1
1
1
1
1
1
1

1
0
0
0
0
0
0
0
0
0
0
0
0
0
0
0
0
0
0
0
0
0
0
0
0
0
0
0
0
0
0
0
0
0
0
0
0
0
0
0
0
0
0
0
0
0
0
0

0
0
0
0
0
0
0
0
0
0
0
0
0
1
1
1
1
1
1
1
1
1
1
1
1
1
1
1
1
1
1
1
1
1
1
1
1
1
1
1
1
1
1
1
1
1

1
1
1
1
1
1
1
1
1
1
1
1
1
1
1
1
1
1
1
1
1
1
1
1
1
1
1
1
1
1
1
1
1
1
1
1
1
1
1
1
1
1
1
1
1
1
1
1

1
1
1
1
1
1
1
1
1
1
1
1
1
1
1
1
1
1
1
1
1
1
1
1
1
1
1
1
1
1
1
1
1
1
1
1
1
1
1
1
1
1
1
1
1
1
1
1

1
1
1
1
1
1
1
1
1
1
1
1
1
1
1
1
1
1
1
1
1
1
1
1
1
1
1
1
1
1
1
1
1
1
1
1
1
1
1
1
1
1
1
1
1
1
1
1

1
2
2
2
2
2
2
2
2
2
2
2
2
2
2
2
2
2
2
2
2
2
2
2
2
2
2
2
2
2
2
2
2
2
2
2
2
2
2
2
2
2
2
2
2
2
2
2

2
2
2
2
2
2
2
2
2
2
2
2
2
0
0
0
0
0
0
0
0
0
0
0
0
0
0
0
0
0
0
0
0
0
0
0
0
0
0
0
0
0
0
0
0
0
0
0

0
0
0
0
0
0
0
0
0
0
0
0
0
0
0
0
0
0
0
0
0
0
0
0
0
0
0
0
0
0
0
0
0
0
0
0
0
0
0
0
0
0
0
0
0
0
0
0

0
0
0
0
0
0
0
0
0
0
0
0
0
0
0
0
0
0
0
0
0
0
0
0
0
0
0
0
0
0
0
0
0
0
0
0
0
0
0
0
0
0
0
0
0
0
0
0

0
0
0
0
0
0
0
0
0
0
0
0
0
0
0
0
0
0
0
0
0
0
0
0
0
0
0
0
0
0
0
0
0
0
0
0
0
0
0
0
0
0
0
0
0
0
0
0

0
5
5
5
5
5
5
5
5
5
5
5
5
5
5
5
5
5
5
5
5
5
5
5
5
5
5
5
5
5
5
5
5
5
5
5
5
5
5
5
5
5
5
5
5
5
5
5

5
5
5
5
5
5
5
5
5
5
5
5
5
4
4
4
4
4
4
4
4
4
4
4
4
4
4
4
4
4
4
4
4
4
4
4
4
4
4
4
4
4
4
4
4
4
4

4
4
4
4
4
4
4
4
4
4
4
4
4
4
4
4
4
4
4
4
4
4
4
4
4
4
4
4
4
4
4
4
4
4
4
4
4
4
4
4
4
4
4
4
4
4
4
4

4
4
4
4
4
4
4
4
4
4
4
4
4
4
4
4
4
4
4
4
4
4
4
4
4
4
4
4
4
4
4
4
4
4
4
4
4
3
3
3
3
3
3
3
3
3
3
3

3
3
3
3
3
3
3
3
3
3
3
3
3
3
3
3
3
3
3
3
3
3
3
3
3
3
3
3
3
3
3
3
3
3
3
3
3
3
3
3
3
3
3
3
3
3
3
3

3
2
2
2
2
2
2
2
2
2
2
2
2
2
2
2
2
2
2
2
2
2
2
2
2
2
2
2
2
2
2
2
2
2
2
2
2
2
2
2
2
2
2
2
2
2
2
2

2
2
2
2
2
2
2
2
2
2
2
2
2
2
2
2
2
2
2
2
2
2
2
2
2
2
2
2
2
2
2
2
2
2
2
2
2
2
2
2
2
2
2
2
2
2
2
2

2
2
2
2
2
2
2
2
2
2
2
2
2
2
2
2
2
2
2
2
2
2
2
2
2
3
3
3
3
3
3
3
3
3
3
3
3
3
3
3
3
3
3
3
3
3
3
3

3
3
3
3
3
3
3
3
3
3
3
3
3
3
3
3
3
3
3
3
3
3
3
3
3
3
3
3
3
3
3
3
3
3
3
3
3
6
6
6
6
6
6
6
6
6
6
6

6
6
6
6
6
6
6
6
6
6
6
6
6
6
6
6
6
6
6
6
6
6
6
6
6
6
6
6
6
6
6
6
6
6
6
6
6
6
6
6
6
6
6
6
6
6
6
6
