# Supplementary material for: How does agonistic behaviour differ in albino and pigmented fish?
Source: PeerJ. 2016 Apr 18;4:e1937. doi: 10.7717/peerj.1937 (PMC4841223; doi:10.7717/peerj.1937)
Supplement: Supplemental Information 2 [file peerj-04-1937-s002.pdf]

treatment	trial	weight1	weight2	category_no	beh_CATEGORY	N_IE
AxA	1	8	10	4	frontdisplay	1
AxA	1	8	10	5	latdisplay	16
AxA	1	8	10	6	bitting	3
AxA	1	8	10	7	chasing	6
AxA	2	9	7	4	frontdisplay	0
AxA	2	9	7	5	latdisplay	5
AxA	2	9	7	6	bitting	0
AxA	2	9	7	7	chasing	7
AxA	3	8	13	4	frontdisplay	7
AxA	3	8	13	5	latdisplay	17
AxA	3	8	13	6	bitting	2
AxA	3	8	13	7	chasing	3
AxA	4	11	10	4	frontdisplay	8
AxA	4	11	10	5	latdisplay	21
AxA	4	11	10	6	bitting	1
AxA	4	11	10	7	chasing	1
AxA	5	11	12	4	frontdisplay	5
AxA	5	11	12	5	latdisplay	10
AxA	5	11	12	6	bitting	1
AxA	5	11	12	7	chasing	3
AxA	6	13	11	4	frontdisplay	3
AxA	6	13	11	5	latdisplay	8
AxA	6	13	11	6	bitting	2
AxA	6	13	11	7	chasing	2
AxA	7	10	12	4	frontdisplay	1
AxA	7	10	12	5	latdisplay	5
AxA	7	10	12	6	bitting	1
AxA	7	10	12	7	chasing	2
AxA	8	12	11	4	frontdisplay	0
AxA	8	12	11	5	latdisplay	3
AxA	8	12	11	6	bitting	0
AxA	8	12	11	7	chasing	1
AxA	9	9	11	4	frontdisplay	3
AxA	9	9	11	5	latdisplay	4
AxA	9	9	11	6	bitting	0
AxA	9	9	11	7	chasing	4
AxA	10	13	12	4	frontdisplay	5
AxA	10	13	12	5	latdisplay	23
AxA	10	13	12	6	bitting	0
AxA	10	13	12	7	chasing	0
AxA	11	9	10	4	frontdisplay	6
AxA	11	9	10	5	latdisplay	11
AxA	11	9	10	6	bitting	0
AxA	11	9	10	7	chasing	1
AxA	12	13	13	4	frontdisplay	1
AxA	12	13	13	5	latdisplay	5
AxA	12	13	13	6	bitting	0

AxA	12	13	13	7	chasing	2
AxA	13	13	13	4	frontdisplay	1
AxA	13	13	13	5	latdisplay	0
AxA	13	13	13	6	bitting	0
AxA	13	13	13	7	chasing	0
AxA	14	10	11	4	frontdisplay	1
AxA	14	10	11	5	latdisplay	0
AxA	14	10	11	6	bitting	0
AxA	14	10	11	7	chasing	0
AxA	15	13	13	4	frontdisplay	2
AxA	15	13	13	5	latdisplay	1
AxA	15	13	13	6	bitting	0
AxA	15	13	13	7	chasing	2
AxA	16	14	15	4	frontdisplay	6
AxA	16	14	15	5	latdisplay	7
AxA	16	14	15	6	bitting	0
AxA	16	14	15	7	chasing	0
AxA	17	7	9	4	frontdisplay	0
AxA	17	7	9	5	latdisplay	0
AxA	17	7	9	6	bitting	0
AxA	17	7	9	7	chasing	1
AxA	18	12	9	4	frontdisplay	3
AxA	18	12	9	5	latdisplay	5
AxA	18	12	9	6	bitting	0
AxA	18	12	9	7	chasing	3
AxA	19	8	13	4	frontdisplay	5
AxA	19	8	13	5	latdisplay	10
AxA	19	8	13	6	bitting	0
AxA	19	8	13	7	chasing	0
AxA	20	15	12	4	frontdisplay	2
AxA	20	15	12	5	latdisplay	7
AxA	20	15	12	6	bitting	0
AxA	20	15	12	7	chasing	0
CxC	1	10	7	4	frontdisplay	0
CxC	1	10	7	5	latdisplay	9
CxC	1	10	7	6	bitting	0
CxC	1	10	7	7	chasing	3
CxC	2	9	10	4	frontdisplay	2
CxC	2	9	10	5	latdisplay	19
CxC	2	9	10	6	bitting	4
CxC	2	9	10	7	chasing	4
CxC	3	7	8	4	frontdisplay	4
CxC	3	7	8	5	latdisplay	8
CxC	3	7	8	6	bitting	1
CxC	3	7	8	7	chasing	7
CxC	4	9	8	4	frontdisplay	12
CxC	4	9	8	5	latdisplay	26
CxC	4	9	8	6	bitting	3

CxC	4	9	8	7	chasing	2
CxC	5	8	8	4	frontdisplay	6
CxC	5	8	8	5	latdisplay	26
CxC	5	8	8	6	bitting	1
CxC	5	8	8	7	chasing	2
CxC	6	8	9	4	frontdisplay	1
CxC	6	8	9	5	latdisplay	9
CxC	6	8	9	6	bitting	0
CxC	6	8	9	7	chasing	1
CxC	7	7	6	4	frontdisplay	3
CxC	7	7	6	5	latdisplay	16
CxC	7	7	6	6	bitting	2
CxC	7	7	6	7	chasing	3
CxC	8	7	8	4	frontdisplay	4
CxC	8	7	8	5	latdisplay	20
CxC	8	7	8	6	bitting	1
CxC	8	7	8	7	chasing	3
CxC	9	9	7	4	frontdisplay	1
CxC	9	9	7	5	latdisplay	1
CxC	9	9	7	6	bitting	0
CxC	9	9	7	7	chasing	3
CxC	10	8	8	4	frontdisplay	2
CxC	10	8	8	5	latdisplay	1
CxC	10	8	8	6	bitting	0
CxC	10	8	8	7	chasing	1
CxC	11	6	6	4	frontdisplay	1
CxC	11	6	6	5	latdisplay	7
CxC	11	6	6	6	bitting	0
CxC	11	6	6	7	chasing	0
CxC	12	7	7	4	frontdisplay	4
CxC	12	7	7	5	latdisplay	13
CxC	12	7	7	6	bitting	0
CxC	12	7	7	7	chasing	2
CxC	13	11	16	4	frontdisplay	0
CxC	13	11	16	5	latdisplay	0
CxC	13	11	16	6	bitting	0
CxC	13	11	16	7	chasing	0
CxC	14	11	15	4	frontdisplay	0
CxC	14	11	15	5	latdisplay	3
CxC	14	11	15	6	bitting	0
CxC	14	11	15	7	chasing	7
CxC	15	9	13	4	frontdisplay	1
CxC	15	9	13	5	latdisplay	10
CxC	15	9	13	6	bitting	0
CxC	15	9	13	7	chasing	1
CxC	16	8	11	4	frontdisplay	2
CxC	16	8	11	5	latdisplay	11
CxC	16	8	11	6	bitting	2

CxC	16	8	11	7	chasing	1
CxC	17	12	10	4	frontdisplay	0
CxC	17	12	10	5	latdisplay	14
CxC	17	12	10	6	bitting	1
CxC	17	12	10	7	chasing	0
CxC	18	7	9	4	frontdisplay	1
CxC	18	7	9	5	latdisplay	12
CxC	18	7	9	6	bitting	0
CxC	18	7	9	7	chasing	0
CxC	19	11	8	4	frontdisplay	9
CxC	19	11	8	5	latdisplay	21
CxC	19	11	8	6	bitting	0
CxC	19	11	8	7	chasing	0
CxC	20	13	7	4	frontdisplay	1
CxC	20	13	7	5	latdisplay	21
CxC	20	13	7	6	bitting	1
CxC	20	13	7	7	chasing	0

weight_diff
2
2
2
2
2
2
2
2
5
5
5
5
1
1
1
1
1
1
1
1
2
2
2
2
2
2
2
2
1
1
1
1
2
2
2
2
1
1
1
1
1
1
1
1
0
0
0

0
0
0
0
0
1
1
1
1
0
0
0
0
1
1
1
1
2
2
2
2
3
3
3
3
5
5
5
5
3
3
3
3
3
3
3
3
1
1
1
1
1
1
1
1
1
1
1

1
0
0
0
0
1
1
1
1
1
1
1
1
1
1
1
1
2
2
2
2
0
0
0
0
0
0
0
0
0
0
0
0
5
5
5
5
4
4
4
4
4
4
4
4
3
3
3

3
2
2
2
2
2
2
2
2
3
3
3
3
6
6
6
6
