# Supplementary material for: How does agonistic behaviour differ in albino and pigmented fish?
Source: PeerJ. 2016 Apr 18;4:e1937. doi: 10.7717/peerj.1937 (PMC4841223; doi:10.7717/peerj.1937)
Supplement: Supplemental Information 3 [file peerj-04-1937-s003.pdf]

treatment	trial	weight1	weight2	distance_CATEGORY	DUR_SE	N_SE	W_diff
AxA	1	8	10	distant	62.8	12	2
AxA	1	8	10	next to	98.98	36	2
AxA	1	8	10	same part	136.73	23	2
AxA	2	9	7	distant	44.14	8	2
AxA	2	9	7	next to	151.26	25	2
AxA	2	9	7	same part	103.17	17	2
AxA	3	8	13	distant	39.44	14	5
AxA	3	8	13	next to	89.08	28	5
AxA	3	8	13	same part	170.23	15	5
AxA	4	11	10	distant	44.01	14	1
AxA	4	11	10	next to	92.16	31	1
AxA	4	11	10	same part	162.55	16	1
AxA	5	11	12	distant	54.03	12	1
AxA	5	11	12	next to	105.78	24	1
AxA	5	11	12	same part	137.73	13	1
AxA	6	13	11	distant	63.82	14	2
AxA	6	13	11	next to	103.69	31	2
AxA	6	13	11	same part	130.4	17	2
AxA	7	10	12	distant	48.05	13	2
AxA	7	10	12	next to	121.25	37	2
AxA	7	10	12	same part	129	24	2
AxA	8	12	11	distant	62.28	8	1
AxA	8	12	11	next to	179.03	19	1
AxA	8	12	11	same part	56.61	11	1
AxA	9	9	11	distant	57.13	14	2
AxA	9	9	11	next to	109.47	38	2
AxA	9	9	11	same part	130.89	24	2
AxA	10	13	12	distant	44.13	7	1
AxA	10	13	12	next to	55.16	20	1
AxA	10	13	12	same part	199.28	14	1
AxA	11	9	10	distant	30.11	10	1
AxA	11	9	10	next to	96.77	24	1
AxA	11	9	10	same part	171.37	15	1
AxA	12	13	13	distant	95.89	15	0
AxA	12	13	13	next to	90.74	38	0
AxA	12	13	13	same part	111.89	23	0
AxA	13	13	13	distant	10.39	5	0
AxA	13	13	13	next to	227.12	15	0
AxA	13	13	13	same part	61.17	9	0
AxA	14	10	11	distant	14.26	5	1
AxA	14	10	11	next to	225.04	15	1
AxA	14	10	11	same part	59.32	10	1
AxA	15	13	13	distant	77.88	27	0
AxA	15	13	13	next to	130.45	50	0
AxA	15	13	13	same part	90.5	23	0
AxA	16	14	15	distant	15.41	8	1

AxA	16	14	15	next to	46.19	21	1
AxA	16	14	15	same part	237.53	13	1
AxA	17	7	9	distant	56.13	9	2
AxA	17	7	9	next to	191.31	20	2
AxA	17	7	9	same part	50.81	10	2
AxA	18	12	9	distant	42.53	3	3
AxA	18	12	9	next to	91.3	14	3
AxA	18	12	9	same part	164.09	11	3
AxA	19	8	13	distant	66.89	8	5
AxA	19	8	13	next to	69.3	25	5
AxA	19	8	13	same part	162.19	17	5
AxA	20	15	12	distant	27.78	6	3
AxA	20	15	12	next to	83.2	24	3
AxA	20	15	12	same part	187.62	18	3
CxC	1	10	7	distant	56	14	3
CxC	1	10	7	next to	128.09	31	3
CxC	1	10	7	same part	114.16	18	3
CxC	2	9	10	distant	14.62	6	1
CxC	2	9	10	next to	76.64	21	1
CxC	2	9	10	same part	207.25	16	1
CxC	3	7	8	distant	58.69	14	1
CxC	3	7	8	next to	79.34	31	1
CxC	3	7	8	same part	160.67	17	1
CxC	4	9	8	distant	20.51	7	1
CxC	4	9	8	next to	57.37	24	1
CxC	4	9	8	same part	220.36	17	1
CxC	5	8	8	distant	18.31	5	0
CxC	5	8	8	next to	36.7	19	0
CxC	5	8	8	same part	243.11	13	0
CxC	6	8	9	distant	122	16	1
CxC	6	8	9	next to	90.34	32	1
CxC	6	8	9	same part	86	17	1
CxC	7	7	6	distant	73.9	10	1
CxC	7	7	6	next to	65.63	19	1
CxC	7	7	6	same part	158.94	9	1
CxC	8	7	8	distant	58.1	10	1
CxC	8	7	8	next to	49.17	27	1
CxC	8	7	8	same part	190.78	17	1
CxC	9	9	7	distant	28.5	8	2
CxC	9	9	7	next to	61.6	15	2
CxC	9	9	7	same part	208.5	7	2
CxC	10	8	8	distant	45.86	15	0
CxC	10	8	8	next to	161.93	34	0
CxC	10	8	8	same part	90.86	19	0
CxC	11	6	6	distant	13	2	0
CxC	11	6	6	next to	75.3	19	0
CxC	11	6	6	same part	210.08	17	0
CxC	12	7	7	distant	19.76	4	0

CxC	12	7	7	next to	49.82	14	0
CxC	12	7	7	same part	229.1	10	0
CxC	13	11	16	distant	0	0	5
CxC	13	11	16	next to	0	0	5
CxC	13	11	16	same part	0	0	5
CxC	14	11	15	distant	27.86	11	4
CxC	14	11	15	next to	87.38	31	4
CxC	14	11	15	same part	183.65	20	4
CxC	15	9	13	distant	25.3	6	4
CxC	15	9	13	next to	118.85	22	4
CxC	15	9	13	same part	154.4	16	4
CxC	16	8	11	distant	45.5	8	3
CxC	16	8	11	next to	128.64	21	3
CxC	16	8	11	same part	124.67	13	3
CxC	17	12	10	distant	91.98	12	2
CxC	17	12	10	next to	83.53	31	2
CxC	17	12	10	same part	122.89	18	2
CxC	18	7	9	distant	0	0	2
CxC	18	7	9	next to	20.74	5	2
CxC	18	7	9	same part	277.56	5	2
CxC	19	11	8	distant	34.93	3	3
CxC	19	11	8	next to	32.77	11	3
CxC	19	11	8	same part	230.2	9	3
CxC	20	13	7	distant	24.5	4	6
CxC	20	13	7	next to	73.62	13	6
CxC	20	13	7	same part	200.87	10	6
